# Effects of mindful breathing meditation on stereotype expression in two randomized controlled double-blinded trials

**DOI:** 10.1371/journal.pone.0347871

**Published:** 2026-04-30

**Authors:** Elena Vieth, Lisa von Stockhausen

**Affiliations:** Department of Psychology, University of Duisburg-Essen, Universitätsstraße, Essen, Germany; Isha Yoga Centre, INDIA

## Abstract

Models of stereotypes and discrimination propose the involvement of attentional control and executive functioning in successfully suppressing discriminatory behavior. A practice that has been shown to improve cognitive control, potentially even after short training durations, is mindfulness meditation, and beneficial effects on stereotype activation and the expression of discriminatory behavior have been reported. In two randomized controlled double-blinded trials, the effects of short mindful breathing meditation trainings (45 minutes in total in Experiment 1, 80 minutes in Experiment 2) on the expression of stereotype-biased behavior were contrasted with relaxation trainings of equal length (progressive muscle relaxation; active control) and listening to podcasts (passive control). Stereotype expression was assessed with the Shooter Task in Experiment 1 and with the Avoidance Task in Experiment 2. Joined analyses of response latencies and accuracy by drift diffusion modeling showed that breathing meditation increased the effect of stereotype bias on decision-making in both experiments by heightening response conflict in stereotype-incongruent trials. At the same time, relaxation reduced biased decision-making in stereotype-incongruent trials. Contrary to our hypothesis, the findings suggested that short trainings in mindful breathing meditation were not beneficial for reducing the effect of stereotype-bias on decision making and that mechanisms outside of cognitive control affected response behavior following the meditation as well as relaxation training. Implications for research in the field are discussed.

## Introduction

Stereotypes are conceptualized as associations and beliefs about traits and qualities of members of a social group that influence thought and behavior towards the group [1,2]. Research has shown that stereotypes already influence early perceptual processes – for example, the social category of race is processed within 200 ms of stimulus presentation [3] – producing a readiness to perceive stereotype-consistent behaviors or characteristics [4]. Such preferred processing can subsequently bias decision-making and behavior. While stereotypes are part of basic categorization processes in the human mind that allow for efficient reduction of informational input from the environment, in the social domain, errors in this process in terms of overgeneralization and prejudice are very costly for stereotyped groups, resulting in, for example, poorer mental and physical health [e.g., 5] or economic inequalities [e.g., 6]. Therefore, the process of stereotyping and how to reduce its harmful effects has received much attention in research.

Cognitive models of stereotyping distinguish between automatic and controlled processes [e.g., 7, 8, 9]. Automatic processes are defined as the unintentional or spontaneous activation of well-established associations or the pre-activation of motor-responses with no deliberate effort. For example, research has shown that behavior displayed by a Black person is more readily associated with threat than identical behavior displayed by a White person [10] and that stereotypes facilitate the perception of anger in Black compared to White faces [11]. In contrast, controlled processes are intentional and more flexible, demand attentional resources, and are limited by cognitive capacity. Cognitive depletion, for example, can reduce controlled but not automatic processes in stereotype tasks, increasing the likelihood of a stereotype-based response [12]. Cognitive models of stereotyping therefore propose that the difference between a biased and an unbiased response is not contingent on stereotypes being activated or not but rather depends on an individual’s willingness and ability to control automatically activated associations, the latter involving attentional [13,14] and executive control processes [15–17]. Accordingly, research on cognitive control in stereotype bias has shown that conflict monitoring and detection [18–20] as well as common executive function ability (i.e., the interplay of inhibition, working memory/updating and task switching) [21–24], are associated with more controlled response behavior on tasks measuring stereotype-biased behavior. Taken together, cognitive models and studies described above highlight the role of attentional control and executive functioning in stereotype expression.

A practice that has been proposed to train cognitive control is meditation within the mindfulness framework [e.g., 25–28]. Mindfulness can be described as a state of paying attention to and being fully aware of the present moment in a non-judgmental way [29]. In focused-attention practices such as the breathing meditation, practitioners focus their attention on the breath. In case of internal or external distractions, they are to let go of the distraction in an accepting, non-judgmental manner and to return their attention to their breath. Because this practice involves constantly observing one’s cognitive processes (e.g., thoughts, feelings, or emotions) to detect distractions from the current attentional focus (e.g., observing the breath), it has been proposed that meditation trains monitoring cognition as well as re-allocating attentional resources in adherence with the current goal set [e.g., 30]. Accordingly, intervention and cross-sectional studies have found enhanced conflict monitoring and resolution following meditation trainings and in expert meditators compared to non-meditators [for reviews, see 31, 32]. Regarding the involvement of cognitive control in stereotype suppression described above, if meditation training improves the ability to detect a conflict between automatically activated stereotypes and the current goal-set, this should increase the individual’s potential to inhibit a prepotent, biased response [17].

Accordingly, meditation training has been found to reduce expression of stereotypical bias. Lueke and Gibson [33] utilized Implicit Association Tests (IATs) to examine automatic associations between a social category (in this study, race and age) and attributes of positive and negative valence, presented in stereotype-congruent (e.g., Black face, tragic) or -incongruent combinations (e.g., Black face, attractive). Following 10 minutes of a focused-attention meditation, participants exhibited reduced automatic activation in racial and age bias compared to a passive control condition, with no differences between conditions in controlling biased responses. Moreover, employing a trust game with simulated interactions during which participants had to decide how much they would trust either a Black or a White partner to make a reciprocally beneficial money transaction, Lueke and Gibson [34] showed that a 10-minute meditation resulted in less differential treatment of Black compared to White partners compared to control conditions. Contrasting these findings is a study by Hunsinger, Christopher [35], who investigated the effectiveness of an eight-week mindfulness-based resilience training for law enforcement officers (including various meditational practices as well as psycho-educational aspects concerning mindfulness, stress and resilience) on performance in the Shooter Task. During the Shooter Task, participants are presented with Black or White targets holding either a gun or a non-harmful object. While the target’s race is irrelevant to the participants’ task of identifying the object as either a weapon or non-harmful, race has been shown to affect response behavior based on stereotypical associations of threat and Black people. Compared to a non-intervention control condition, the authors found no effects of mindfulness training on response latencies and behavior. However, the authors noted a lack of stereotype bias in response behavior at baseline, indicating that there might have been no stereotype bias in their sample that could have been affected by mindfulness training. Thus, while results by Lueke and Gibson [33], Lueke and Gibson [34] suggest that mindfulness may modulate stereotype activation and bias expression, the results by Hunsinger, Christopher [35] are less clear.

Therefore, the current study aimed to investigate the effects of enhanced cognitive control on stereotype bias expression via mindfulness in the form of brief breathing meditation trainings in two randomized controlled double-blinded trials. As outlined above, we propose that the underlying mechanism for an improvement in stereotype suppression would be enhanced conflict monitoring and detection as a result of mindful breathing meditation. We addressed challenges of research in the field in the following ways: In Experiment 1, the Shooter Task [36] was utilized to assess the effects of breathing meditation on stereotype-biased response behavior. In this task, participants are asked to quickly make a shooting decision whenever a target (Black or White men) holding an object (gun or non-harmful object) appears. Responses typically reveal a shooter bias, meaning that participants are faster and/or more accurate in their shooting decisions for armed Black compared to White targets and, conversely, are faster and/or more accurate in their decisions to not shoot unarmed White compared to Black targets [e.g.,  37–40]. Modeling response behavior during the Shooter Task by means of drift diffusion modeling [DDM; 41, 42] furthermore allowed us to investigate the influence of racial stereotypes on the process of decision-making, taking both response speed and accuracy into account. So far, studies utilizing DDM to investigate decision-making during the Shooter Task have found greater evidence accumulation towards a correct response in stereotype-congruent versus -incongruent trials and that participants exhibit greater response control (i.e., trading speed for accuracy) for Black compared to White targets in an effort to control stereotype-biased behavior [43–46]. These findings suggest that stereotypes directly influence the efficiency of information processing, as well as the amount of evidence required to make a decision, rather than facilitating motor-response preparation [e.g., 46, but see 47], or eliciting an a-priori decision bias before information relevant for responding is processed [e.g., 46, 48]. We propose that training cognitive control abilities through mindful breathing meditation could beneficially influence information processing during the Shooter Task by improving attentional monitoring and (re-) allocation of attentional resources to task-relevant information (i.e., identification of the object) as well as by subsequently improving inhibition of processing task-irrelevant information about target race. Such improvements are postulated to reduce or diminish the effects of stereotypes on response behavior. In Experiment 2, we utilized the Avoidance Task by Essien, Stelter [49] to investigate whether the postulated effects translate to different discriminatory behavior. During the Avoidance Task, participants are asked to avoid (rather than shoot) targets (Turkish or German) with knives, while targets holding non-harmful objects are to be approached. In line with the described shooter bias, Essien et al. found increased response speed in stereotype-congruent (e.g., Turkish armed) compared to incongruent trials (e.g., German armed). In our study, the Avoidance Task was employed to increase the ecological validity of the scenario in a German sample by utilizing German and Turkish targets [the latter being a minority group in Germany that is stereotypically associated with threat or danger, [50, 51]. Again, drift diffusion modeling was used to investigate the underlying processes of decision-making. We assessed positive and negative affect with the Positive and Negative Affect Schedule [PANAS; 52] to control for possible influences of mood on attentional and executive control [53]. As this study took place during the COVID-19 pandemic, subjectively perceived pandemic impact and strain were assessed by self-report to control for possible effects on intervention effectivity [54,55]. In Experiment 2, we additionally assessed participants’ motivation to control prejudiced behavior, which previously has been identified as affecting the willingness to exert cognitive control over activated stereotypes [e.g., 23].

In both experiments, we utilized brief breathing meditation trainings, which effectively isolate the direct effects of state mindfulness without confounding factors (e.g., longer training also encompasses psycho-educational and motivational aspects). Since the dose-response relationship between mindfulness meditation and enhanced cognitive control is still up for debate [e.g., 56, 57, 58], we increased training frequency and total training duration from Experiment 1 (three practice sessions of 10, 15 and 20 minutes) to Experiment 2 (four practice sessions of 20 minutes each). We implemented a pre-post experimental design to assess the effects of short breathing meditation trainings in contrast to an active (induction of relaxation) and passive (podcast listening) control group. Since the experiment was conducted during the COVID-19 pandemic, meditation/relaxation practice or podcast listening and testing was delivered online. It was hypothesized that if improving conflict monitoring and detection through breathing meditation is effective in reducing the expression of stereotypes, such improvements may be reflected in decision-making processes during both the Shooter and the Avoidance Task in the following ways: Enhanced conflict monitoring and successful (re-) allocation of attentional resources to task-relevant information should decrease the impact of task-irrelevant information (i.e., target race or ethnicity) on information processing. Furthermore, increased controlled responding (speed-accuracy tradeoffs) could indicate participants’ effort to exert cognitive control over biased information processing. How these improvements would be reflected in the parameters of the DDM will be outlined below.

## Methods

### Sample Size Justification

As outlined by [59], estimating power in hierarchical drift-diffusion models typically requires simulation-based approaches that depend heavily on assumptions about parameter distributions, model structure, and prior specifications. Given the novelty of our study design and the absence of precedent for hierarchical drift-diffusion models in stereotype research with meditation interventions, such simulations were not feasible. However, hierarchical drift diffusion modeling is explicitly recommended in the literature for two-choice response time tasks involving moderate sample sizes, as the Bayesian structure supports more accurate estimation of group- and subject-level effects by pooling information across individuals [46,48,60,61].

We followed established practices in the field to ensure model convergence and parameter stability through diagnostics [62–65]. Separate models were estimated for each condition, simplifying the model structure and aligning with the simulation-based guidance by Wiecki and colleagues [59], who report robust parameter recovery of drift-rate effects with 20–30 participants and approximately 80 trials per condition for medium to large effect sizes. Based on previous findings showing medium effect sizes in bias reduction following short mindfulness inductions [33], we also expected to find medium effects. Moreover, the Shooter and Avoidance Tasks are known to elicit strong stereotype-related processing, and prior drift-diffusion model research using the Shooter Bias paradigm has demonstrated medium to large effects in drift parameters [43].

### Participants

The samples consisted of 96 participants in Experiment 1 (74 female, 1 non-binary, M_age_ = 23.5, SD_age_ = 5.43) and 69 participants in Experiment 2 (57 female, 1 non-binary, M_age_ = 22.0, SD_age_ = 5.62). In Experiment 1, 87.9% of participants indicated a German origin, and 12.1% reported other backgrounds, including countries of Eastern European, Middle Eastern, and Southern European origin. In Experiment 2, 89% of participants reported to be of German origin, and 11% reported other backgrounds, following a similar distribution as in Experiment 1. Recruitment was conducted through social and university internal message boards for research participation. Participants meeting the inclusion criteria of being at least 18 years old and not having practiced mindfulness or any other form of meditation regularly during the last three months were contacted by e-mail to set up dates for online participation. Participants received access to the online research environment via a link sent by e-mail on the date they had chosen for their first measurement point. The e-mail also included further information about participation in online research (appropriate environment for participation from home and technical requirements). The link directed participants to a page with written information about the methods and tests included in the experiment, data protection standards and research ethics. Participants could begin with the pre-measurement after providing written informed consent and agreeing to data storage. The current standards of the General Data Protection Regulation of the European Union (GDPR 2016/679) were met for data collection, storage and anonymization. The ethical commission of the Department of Psychology at the University of Duisburg-Essen approved the experiment. As experiments were conducted one year apart from each other, it was possible for participants to take part in both studies. Based on participation codes and demographics, we identified 11 participants who took part in both experiments. As the effects of short meditation interventions are not considered stable, and participants were screened for whether they meditated on a regular basis, double participation should not have affected the results of the present study. Nevertheless, in response to reviewer concerns, we conducted a sensitivity analysis excluding these participants and re-estimating the hierarchical drift diffusion models based on the reduced sample. Results remained largely robust, particularly for the key drift rate parameters. Detailed results of the sensitivity analysis are available in the data repository.

### Assessment of stereotype activation and expression

Reaction time tasks were programmed with OpenSesame [66; version 3.3.11] and presented using a JATOS interface [67; version 03.06.2001]. Participants were instructed to wear headphones for noise reduction and informed that the reaction time tasks could be accessed from desktop computers only (this includes participation from laptop computers provided they were stationed on tables or used as desktops), as the responses were recorded via participants’ keyboards.

#### Shooter Task.

**Fig 1:**
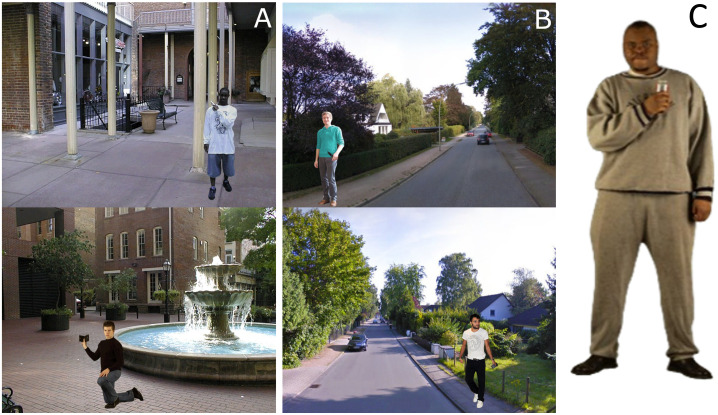
Task Stimuli. Example stimuli from the Shooter Task (Column A, C) and the Avoidance Task (Column **B)**. Background images in (A) are used with permission from Dr. Joshua Correll. The depicted targets in **(A)**, along with images in (B) and **(C)**, were generated by the authors using AI text-to-image software (for details see S3 Appendix).

The Shooter Task was developed by Correll, Park [36] to investigate the effect of race on shooting decisions. During the task, participants are presented with images of Black or White men who are either holding a gun or a non-threatening object such as a cell phone or a wallet (see Fig 1A & 1C). Each time the participant is presented with a target on the computer screen, they must quickly decide whether the person is holding a gun or a non-threatening object and indicate their decision by pressing a button. The stimulus material and design were adopted from Correll, Park [36]. While in the original task, participants are asked to indicate a ‘shooting decision’ by button press, we decided to instruct participants to simply indicate the presence or absence of a gun by pressing a respective key. This was done to obtain a higher ecological validity in a German sample. Compared to the United States of America, Germany has stricter gun laws regarding the process of obtaining a mandatory gun ownership license and with citizens allowed to carry concealed guns only under very specific circumstances. Therefore, most citizens are unlikely to ever come into contact with a gun, let alone have to decide whether or not to shoot another human being. For instance, in 2022, the German Federal Criminal Police Office reported that only 0.64% of violent crimes involved firearms [68] while the figure was 28.4% in the United States [69]. Each trial began with a fixation point, followed by a random number of background images (ranging between one and four, showing landscapes of North American cities) with random duration (500–1000ms). Afterwards, the target was superimposed on another randomly selected background image. Targets were presented in various positions (standing or crouching). Response behavior was rewarded or punished following a pay-off matrix. A hit (correctly indicating a target with a gun) earned 10 points, and a correct rejection (indicating “no gun” for a target holding a non-threatening object) earned 5 points. A false alarm (falsely indicating the presence of a gun for an unarmed target) led to a deduction of 20 points, and a miss (falsely indicating “no gun” for an armed target) resulted in a loss of 40 points. The payoff matrix was used to emphasize a possible threat, with the strongest punishment (−40 points) in case of missing an armed target. Furthermore, a timeout penalty (subtracting 10 points if participants failed to respond within 850 ms after target presentation) reinforced timely responding. Participants were instructed to respond as accurately and as quickly as possible.

Participants were instructed to place their left and right index fingers on the ‘A’ and ‘L’ keys and indicate target identification of either a non-threatening object or a gun, respectively, for each trial. A session consisted of three practice trials and a single block of 80 trials. Each combination of target race (Black, White) and object condition (gun or no gun) was represented with 20 trials. Participants were instructed to fixate on the fixation cross at the beginning of each trial and were informed that they would receive a score based on their performance. The total task duration was approximately 10 minutes.

#### Avoidance Task.

The Avoidance Task by Essien, Stelter [49] is an adaptation of the shooter bias paradigm to investigate the effect of target ethnicity on approach-avoidance behavior. During the task, participants are presented with images of German or Turkish men holding a silver or black knife or a non-threatening object (silver digital camera, aluminum thermos, black wallet, or black cell phone; examples are presented in Fig 1B). Stimulus material and design were adopted from Essien, Stelter [49]. The background images depict German cities and show empty streets with sidewalks on both sides of the street. Half of the targets are superimposed on the left and half on the right sidewalk, with target position counterbalanced for object type and target ethnicity. The general task procedure, trial sequence and pay-off matrix were adopted from the Shooter Task as described above; however, the response indication for the Avoidance Task differs. Participants are instructed to place their left and right index fingers on the ‘A’ and ‘L’ keys, respectively, and are told that their own position in a trial (i.e., left or right sidewalk) is always identical to the position of the target. Participants are instructed to quickly decide whether the person is holding a knife or a non-threatening object and indicate their decision to either avoid the target by crossing to the other side of the street (i.e., pressing the opposite-side key) or to approach the target by staying on the same side of the street (i.e., pressing the same-side key). For example, if an armed target appeared on the left side of the street, participants would correctly indicate to avoid the target by pressing the right (‘L’) key.

A session consisted of 20 practice trials and a single block of 80 trials. Each combination of target ethnicity and target object was represented with 20 trials. Participants were instructed to fixate on a fixation cross in the center of the screen at the beginning of trials and were informed that they would receive a score based on their performance. The total task duration was approximately 10 minutes.

### Questionnaires

The Positive and Negative Affect Schedule [52, German version: 70] was used to assess possible influences of mood [53] on attentional and executive control. The PANAS measures positive and negative affective states on two scales with ten items each. Participants are to indicate how well each affective state (e.g., “scared”) applies to them currently on a 5-point Likert-type scale.

Experiment 2 additionally included a scale measuring motivation to act without prejudice [Motivation zu vorurteilsfreiem Verhalten; [71]. The one-factorial scale measures aspects of the motivation to control prejudiced behavior with 16 items. Participants have to indicate how well a statement applies to them on a 5-point Likert-type scale (e.g*., “If I have thoughts or emotions that discriminate against others, I keep them to myself*.”).

Sociodemographic questions included participants’ age, gender, marital status, education, employment, and number of people in the household. Subjectively perceived impairment due to the COVID-19 pandemic (work/personal) and subjectively perceived strain due to the COVID-19 pandemic (work/personal) were assessed to control for possible effects on training effectiveness.

Additional questionnaires included in the procedure but not reported here were the Five-Facet Mindfulness Questionnaire [FFMQ; 72, German Version: 73], the Smith Relaxation States Inventory 3s [SRSI3s; 74, German version: 75], the state anxiety scale of the State-Trait-Anxiety Inventory [STAI; 76, German Version: 77] and the HEXACO scales emotionality, openness to experience and agreeableness from the short version of the HEXACO Personality Inventory [HEXACO-60; [78], German Version: [79]. Furthermore, both experiments included assessment of the executive functions of inhibition [Continuous Performance Task-II; [80, 81], updating [N-Back Task; 82] and task switching [Number-Letter Task; [16, 83], results of which are reported elsewhere [58]. While only participants who had not engaged in meditation and relaxation practices regularly within the past three months were included in the study, prior experience beyond this timeframe was recorded for all included participants. Analyses reported elsewhere [58] indicate that such prior experience did not affect outcomes on cognitive tasks.

### Sound and fidelity checks for online research

The quality of the audio recordings was ensured by producing them under controlled conditions, matching volume levels across files, and testing playback fidelity on multiple devices and across different browsers. Before training or podcast deliverance, participants completed a sound check (in which they had to identify an animal noise correctly) to ensure that they could hear instructions correctly and adjust the audio volume before audio delivery. Participation continued only after a successful sound check; otherwise, participants were instructed to contact the experimenter to resolve technical issues. Participants were further instructed to conduct the experiment in a quiet and disturbance-free environment and received information about appropriate sitting posture and the usage of headphones for noise reduction. After participants agreed that they had read and would adhere to these instructions, they could start the training (breathing meditation or PMR) or podcast listening by pressing a play button.

Participants’ compliance was monitored by recording whether all audio files were played to completion. Participants received full compensation only if all audio instructions, tasks, and questionnaires were completed; otherwise, partial credit was granted. Participants who did not receive the total training dosage were excluded from the final sample. At the end of each practice session and measurement point, participants reported their ability to follow the audio instructions and the occurrence of disturbances during participation (e.g., noise, interruptions, issues with internet connection) to ensure the quality of data collection. If disturbances occurred, participants were asked to rate the intensity on a 4-point Likert-type scale (weak to strong). To ensure that participants would execute time-sensitive measures correctly, they were given written guidance prior to every RT task, asked to follow all task-specific instructions with regard to taking breaks and received reminders between tasks on appropriate testing conditions (i.e., posture, screen position, distraction-free environment and use of headphones).

### Research design

Both experiments employed 3 (breathing meditation, PMR or podcast listening) x 2 (measurement point) experimental designs. Mindful breathing meditation was the experimental condition, PMR was the active control condition and listening to podcasts was the passive control condition. PMR was selected as a mind-body practice that could plausibly be administered at the same length and frequency as the breathing meditation, but that should not improve cognitive control. Podcast listening was selected as a passive control condition to give participants something to do that did not require any practice, therefore controlling for overall engagement and effects of repeated testing. All participants were given the same information about participating in a concentration training and were unaware of the presence of different experimental conditions. Participants were randomly assigned to an experimental condition within the online experimental environment. Accordingly, both experiments qualify as randomized controlled double-blinded research designs. Deliverance of training (or podcast listening) was three times during Experiment 1 (after pre-measurement, between pre- and post-measurement, and before post-measurement) and four times during Experiment 2 (one additional session between pre- and post-measurement compared to Experiment 1).

### Experimental conditions

In both experiments, the trainings were delivered repeatedly so that participants could become familiar with the respective practice and so that the postulated short-term effects of the trainings would surface at post-measurement. Delivery took the form of pre-recorded audio files. During Experiment 1, three doses of breathing meditation, PMR or podcast listening were delivered on different days over a period of five days. Trainings and podcasts were delivered with increasing length (10, 15 and 20 minutes). Variations in training length were achieved by shortening a 20-minute recording to 10- and 15-minute versions, keeping trainings as standardized as possible, and avoiding variations in speaker voice or speaking rate. During Experiment 2, four doses of equal length (20 minutes) were delivered within five days, thereby achieving a longer total training duration while keeping the delivery timeframe constant. These adjustments in training frequency and duration were implemented to investigate under which conditions, and for which cognitive processes, short-term meditation trainings produce measurable effects [58]. Specifically, the study aimed to investigate whether short-term meditation interventions can modulate automatic and controlled cognitive processes depending on the intensity of training exposure.

#### Audio instructions.

In line with the framework by Kabat-Zinn [84], the breathing meditation instructions focused on being aware of the present moment with an accepting and non-judgmental attitude and observing the in- and outflow of the breath. Instructions included sentences such as: “*Now focus your attention on your breath. Breathe in and breathe out and be aware of this process of breathing*.”. Furthermore, instructions included noticing and letting go of thoughts and emotions that may occur during the practice, for example: “*Thoughts of all kinds may come to your mind. This is totally fine. Once you’ve taken note of this, simply direct your attention back to your breathing*” (more detailed information on both the breathing meditation and PMR instructions can be found in S1 Appendix). Meditation instructions were recorded by speakers (both male and female) trained by the authors to deliver meditation instructions with a calm voice and appropriate intonation.

During PMR [85], practitioners were instructed to tense a muscle group (e.g., the upper thighs) for five to ten seconds with the inhale and to release the tension with the exhale. Instructions include phrasing such as: “*Now clench your left hand into a fist. Hold the tension for five seconds, paying attention to the feeling of tension, while the rest of the body is relaxed.*” A PMR session usually starts with the lower extremities and gradually progresses upwards through the body, leaving ten to twenty seconds to focus on the changes in physiological experience between muscle groups. PMR recordings were provided by two experienced trainers [male and female; 86, 87]. To ensure a clear distinction between the breathing meditation and relaxation condition, the meditation instructions excluded phrases implying or directly instructing relaxation. Likewise, PMR recordings were adjusted so they did not include mindfulness-related phrasings (e.g., instructions to accept the present experience).

Four podcasts concerning historical sites and unique landscapes were utilized in both experiments. To ensure the podcasts’ adequacy for use in a passive control condition, they were pretested to ensure that they did not elicit strong positive or negative emotional reactions (levels of arousal and valence were assessed) and evoked medium levels of interest and engagement. In both experiments, the podcasts were presented randomly with the constraint that participants would not be presented with the same podcast twice.

### Procedure

The general procedure was similar in both experiments, with differences noted where relevant. On their first participation day, participants were greeted in writing and provided with the written informed consent form. After giving their consent, the first experimental session started with questionnaires and reaction time tasks presented in the following order: sociodemographic questions, SRSI3s, CPT-II, STAI, PANAS, N-back Task, FFMQ-D, Number-Letter Task, HEXACO-60 subscales, Shooter Task (Experiment 1) or Avoidance Task (Experiment 2). Manipulation checks and assessments of compliance and fidelity were placed throughout the experiment. The sequence was the same across experiments, measurement points and participants. Participants were allowed to take self-paced breaks between questionnaires and tasks. After completing the pre-measurement, participants performed the sound check and received the first audio session (breathing meditation PMR, or podcast). The second session (and third session in Experiment 2) took place within the same week and only consisted of the second meditation/PMR practice or podcast listening. The last session began with breathing meditation or PMR practice or podcast listening and ended with the post-assessment of all tasks and questionnaires (completing the tasks and questionnaires took about 90 minutes). After completion, participants were debriefed, thanked, and received course credit.

## Statistical analyses

**Fig 2:**
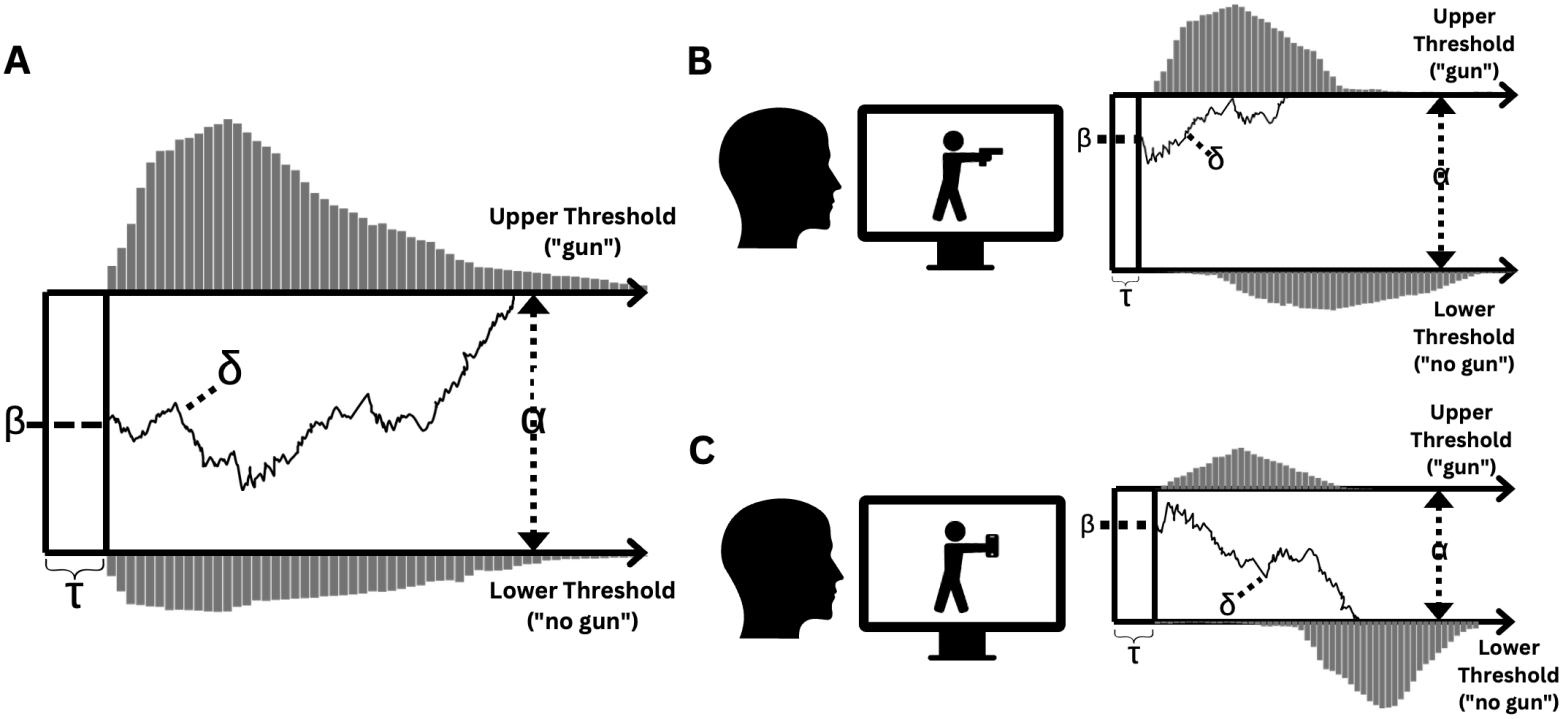
Drift Diffusion Model. General representation of the Drift Diffusion Model **(A)**, of a correct decision to indicate a gun (B) and a correct decision to indicate “no gun” **(C)**.

Drift diffusion models [41,42] describe binary decision-making during a speeded task as a process of continuous evidence accumulation (see Fig 2A): For each trial of a given task, information from the stimulus is extracted and evidence for a decision accumulates (delta, drift rate) until a threshold (alpha) for one of two decisions is met. Additionally, the DDM accounts for an individual’s initial bias towards one of the two decisions at the beginning of evidence accumulation (beta; start point) and for reaction time components unrelated to decision-making (e.g., motor components or basal stimulus processing; non-decision time, tau). We used Bayesian hierarchical DDMs, which estimate posterior distributions using Markov Chain Monte Carlo (MCMC) simulations [88].

Drift diffusion models assume constant drift rates within trials, fixed decision boundaries, and stationarity of parameters across trials within conditions. While these assumptions simplify the decision-making process, they are well-supported in two-choice reaction time tasks like those employed in this study [41]. Our models were adopted from previous work in which drift diffusion models for the shooter bias paradigm were utilized [46,48], drawing parameters from distributions on individual and condition levels. Priors for subjects and trial conditions were truncated normal distributions, with the truncation ensuring that parameters fall within theoretically plausible values (see Table 1). Priors for the group-level distribution were uniform distributions for the mean (aligned with the central tendencies of the respective parameters’ normal distribution) and non-informative gamma distributions with both shape and rate parameters set to 0.001 for the precision. Separate models were run for conditions at pre- and post-measurement. Further specifications of priors, JAGS and R Code for model estimation are available at the Open Science Framework [OSF; 89]: https://osf.io/nwfyt/overview?view_only=5ba3e160eb5a4bef9c9c568e353c9f93. Detailed model diagnostic plots can be found in the supplementary material (S4 Appendix).

**Table 1:** Parameters of the Drift Diffusion Model.

Parameter	Specification
Threshold separation (α)	Separation of decision thresholds, with 0 < α. Larger values reflect more accurate but slower decisions. Modeled separately for target race.
Relative start point (β)	Location of starting point for evidence accumulation relative to thresholds, with 0 < β < 1. Values of β > .5 indicate a bias to indicate the presence of a gun and vice versa. Modeled separately for target race.
Drift rate (δ)	Quality of extracted evidence in favor of a response, with −∞ < δ < ∞. Negative values reflect stronger evidence in favor of “no gun” and vice versa. Higher absolute values reflect more efficient processing. Modeled separately for target race x object type.
Non-decision time (τ)	Includes the time spent encoding the stimulus, executing a response, and any other contaminant pre- and post-decisional processes, with 0 < τ. Modeled separately for target race x object type.

MCMC chain convergence was assessed by the potential scale reduction factor (PSRF), chain resolution by effective sample sizes (ESS) and Monte Carlo standard errors. All point estimates of the PSRF for condition and individual level parameters = 1, the multivariate PSRF for each model was < 1.2, indicating full convergence for all parameters. Most ESS values were ≥ 10.000, indicating accurate and stable estimates of the posterior distribution. Monte Carlo standard errors were ≤ 0.01, indicating stable posterior mean estimations for all parameters. Individual parameters were extracted for further analysis through repeated-measures analyses of variance (ANOVA) with an added error term to account for subject-dependent variance. The Kenward-Roger approximation was utilized to account for heightened levels of kurtosis in the distributions of the dependent measures [90,91]. Planned comparisons between conditions were done by estimated marginal mean (EMM) contrasts. For p-value adjustment, the Tukey method was selected.

Data were modeled in R (R Core Team, 2020; version 4.0.2). EMMs were calculated with the emmeans package [92]; version 1.4.8.], and results figures were obtained using the ggplot2 package [93; 3.3.6]. HDDMs were estimated using MCMC sampling via the rjags [94; version 4.10] and runjags packages [95; version 2.0.4–6] with the Wiener module extension [96].

A specification of the estimated parameters and their interpretation can be found in Table 1. How stereotype bias affects decision-making during the Shooter Task will be outlined in the following.

Higher delta values reflect stronger evidence for the respective decision, while values closer to zero reflect ambiguous evidence interpretation or guessing. Accordingly, delta may be interpreted as a measure of perceptual sensitivity or task difficulty and has been shown to be affected by stimulus discriminability, with lower values of delta for stimuli that are harder to discriminate [46,97,98]. Thus, higher absolute levels of delta generally reflect more efficient information processing for a correct decision. Stereotype bias has been shown to facilitate information processing in that evidence for the correct decision accrues more efficiently in stereotype-congruent trials [43–46]. Suppose a Black target is depicted with a gun in Fig 2B (stereotype congruent) and a phone in Fig 2C (stereotype incongruent). Evidence accumulation for the correct “gun” decision in 2B is facilitated by stereotype bias (i.e., delta is steeper). In contrast, evidence accumulation for the correct decision do indicate “no gun” in 2C is not (i.e., greater fluctuation in delta between the thresholds in either direction). On the level of response behavior, this would be characterized by shorter response time and greater accuracy in stereotype-congruent compared to incongruent trials. Since target race represents a task-irrelevant category during the Shooter Task, improved conflict monitoring and resolution through meditation training should enhance attention allocation to task-relevant information and diminish the effect of task-irrelevant information and thus reduce the effect of stereotype bias on information processing towards a correct response. Delta was modeled separately for all combinations of target race and object type.

The larger the threshold separation of the responses, the more evidence is required on a given trial to reach a decision. Alpha thus reflects response caution or a speed-accuracy trade-off; for example, shifting participants’ motivation to respond more accurately can lead to increased threshold separation [97,98]. Conversely, restricting the response window during the Shooter Task has been shown to motivate fast responding, resulting in lower alpha values [46]. Furthermore, studies have reported larger alpha for Black compared to White targets [44,46,47], interpreted as heightened response caution (i.e., more controlled responding) for Black targets, reflecting an attempt to counteract racial bias during the Shooter Task. As depicted in Fig 2B, heightened threshold separation increases the distance between the starting point and the threshold for either decision. Accordingly, larger levels of evidence need to be accumulated for either decision, reflected in slower but more accurate responses. In Fig 2C, lower threshold separation decreases the distance between the decision boundaries; accordingly, less evidence for either decision must be accrued, reflected in faster but more error-prone responses. Regarding possible effects of meditation training on threshold separation, we propose that heightened conflict detection may influence decision-making in that participants exert heightened control in response behavior. This means that participants would trade response speed for accuracy and that more information would be accumulated before a decision threshold is reached (i.e., heightened threshold separation). Alpha was modeled for Black and White targets separately.

Values of beta greater than 0.5 shift the start point of evidence accumulation closer to the threshold for a “gun” response and thus reflect an initial bias to detect a weapon, irrespective of the presented target information. Reversely, beta values smaller than 0.5 reflect an initial bias for “no gun”, as evidence accumulation starts closer to the “no gun” threshold. As during the Shooter Task, a pay-off matrix with a relatively greater reward for correctly detecting a gun and relatively greater punishment for not detecting a gun is used, an initial “shooting bias” has been found [44, 46; but see 43]. Thus, as depicted in Fig 2B and 2C, an initial bias toward gun detection will bias the subsequent evidence accumulation process in that the threshold for making a “gun” decision is reached faster and more frequently, resulting in facilitation of a correct (gun trials) or incorrect response (non-gun trials), respectively. Beta was estimated for Black and White targets separately. Since beta reflects a response bias prior to the accumulation of evidence for either decision (i.e., prior to information processing), target race, or stereotype bias for that matter, is unlikely to affect initial bias [e.g., 46]. Accordingly, no hypothesis was formulated for beta.

The parameter tau provides an estimation of the duration of processes not related to decision-making during each trial (e.g., stimulus encoding and motor processes related to response execution). A robust finding for the Shooter Task is that non-decision time is shorter for gun compared to non-gun trials [43,44,46], which has been attributed to a faster encoding of the relatively uniform category of gun stimuli (as in Fig 2B) compared to the broader category of non-gun stimuli (e.g., phones, soda cans, wallets; as depicted in Fig 2C) presented in the task. Since tau encompasses several processes, an interpretation of underlying causes for changes in tau can only be speculative and no hypothesis was made for effects of meditation training. Tau was modeled separately for all combinations of target race and object type.

Modeling decision-making during the Avoidance Task was based on the same DDM approach described above.

### Results Shooter Task

The results section will focus on analyses relevant to our hypotheses (i.e., effects of measurement point, which reflect effects of practice and repeated testing, and interactions between measurement point and condition, which reflect differential effects of training). As we hypothesized that effects of breathing meditation on stereotype bias in decision-making would affect either drift rate (i.e., quality of information processing) or threshold separation (i.e., amount of evidence required for decision-making), difference scores were calculated across respective target conditions for the parameters delta and alpha to assess a possible influence of stereotype bias or target race. Furthermore, separate analyses for target conditions will be provided to further understanding of possible changes in difference scores. For initial bias (starting point; beta) and non-decision time (all processes unrelated to the decision process; tau), previous studies have provided no evidence for an effect of stereotype during the Shooter Task [43–46], and accordingly, the present study did not formulate hypotheses regarding these parameters. Supplementary analyses for beta (initial bias) and tau (non-decision time) can be found in the supplements (S2 Appendix); in summary, the results indicated no effect of target race on initial bias or non-decision time in the Shooter Task.

To investigate stereotype bias in drift rate (i.e., stereotypic drift rate, Delta_stereotypic_), we applied the method for calculating difference scores from Correll, Wittenbrink [43]: Delta_stereotypic_ = [Delta_Black armed_ – Delta_White armed_] – [Delta_Black unarmed_ – Delta_White unarmed_]). Positive values indicate a positive effect of stereotype congruency on evidence accumulation (i.e., more efficient decision-making for congruent compared to incongruent trials). For threshold separation (alpha), difference scores (Alpha_Black_ – Alpha_White_) were calculated to assess differences in the processes by target race, with positive values reflecting greater threshold separation for Black compared to White targets. Prior to modeling, reaction times smaller than 100 ms were removed. No participants needed to be excluded. This resulted in a data loss of 0.97%. Only significant effects with p ≤ 0.05 are reported.

### Positive and negative affect

To assess possible influences of mood on cognitive control, the PANAS positive and negative affect scores were examined with analyses of variances (measurement point x condition). Results showed no effect of measurement time by condition (see Table 2). Thus, PANAS scores were not included as covariates in the analyses reported below.

**Table 2:** Shooter Task: Significance Testing for Fixed Effects for the PANAS Scales utilizing Analysis of Variance with Kenward-Rogers correction.

	Sum of Squares	df	Mean Square	*F*	*p*
**PANAS Positive Affect**					
Measurement Point	0.13	1, 93	0.13	0.62	0.435
Condition	0.48	2, 93	0.24	1.16	0.319
Measurement Point x Condition	0.64	2, 93	0.32	1.56	0.215
**PANAS Negative Affect**					
Measurement Point	0.25	1, 93	0.25	1.25	0.267
Condition	0.96	2, 93	0.48	2.35	0.101
Measurement Point x Condition	0.19	2, 93	0.10	0.48	0.622

### Drift rates (delta)

**Fig 3:**
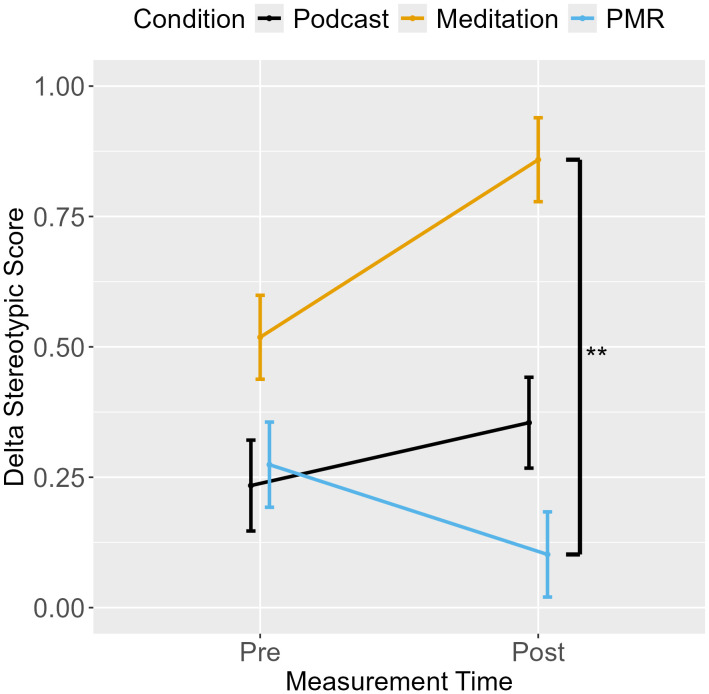
Shooter Task: Stereotypic Drift Rate from Pre- to Post-Measurement.

An ANOVA with stereotypic drift rate as the dependent variable indicated an interaction between measurement point and condition (see Table 3). Planned comparisons (*EMM*_t1_- *EMM*_t0_) showed that breathing meditation yielded an increase in the effect of stereotype bias on evidence accumulation compared to PMR, which exhibited a decrease. No other group comparisons reached significance(Table 4, Fig 3).

**Table 3:** Shooter Task: Mixed ANOVAs for the DDM Parameters Delta and Alpha in the Shooter Task.

	Sum of Squares	df	Mean Square	*F*	*p*
**Delta Stereotypic**					
Measurement point	0.44	1, 93	0.44	2.01	0.159
Condition	9.28	2, 93	4.64	21.07	**<.001**
Measurement point x Condition	2.21	2, 93	1.11	5.02	**0.008**
**Delta White unarmed**					
Measurement point	29.36	1, 93	29.36	182.42	**<.001**
Condition	0.10	2, 93	0.05	0.31	0.731
Measurement point x Condition	4.04	2, 93	2.02	12.55	**<.001**
**Delta Black unarmed**					
Measurement point	26.85	1, 93	26.85	187.93	**<.001**
Condition	0.75	2, 93	0.37	2.61	0.079
Measurement point x Condition	4.58	2, 93	2.29	16.02	**<.001**
**Delta White armed**					
Measurement point	4.48	1, 93	4.48	58.16	**<.001**
Condition	0.20	2, 93	0.10	1.31	0.274
Measurement point x Condition	3.59	2, 93	1.80	23.33	**<.001**
**Delta Black armed**					
Measurement point	6.48	1, 93	6.48	75.67	**<.001**
Condition	0.86	2, 93	0.43	5.05	**0.008**
Measurement point x Condition	0.76	2, 93	0.38	4.42	**0.015**
**Alpha Difference Score**					
Measurement point	0.14	1, 93	0.14	11.73	**<.001**
Condition	0.03	2, 93	0.01	1.13	0.328
Measurement point x Condition	0.04	2, 93	0.02	1.88	0.159
**Alpha White**					
Measurement point	0.72	1, 93	0.72	139.29	**<.001**
Condition	0.27	2, 93	0.14	26.24	**<.001**
Measurement point x Condition	0.05	2, 93	0.02	4.43	**0.015**
**Alpha Black**					
Measurement point	1.49	1, 93	1.49	144.23	**<.001**
Condition	0.26	2, 93	0.13	12.79	**<.001**
Measurement point x Condition	0.03	2, 93	0.01	1.23	0.297

**Table 4:** Experiment 1: Planned Comparisons for Evidence Accumulation (Delta) and Threshold Separation (Alpha).

	Podcast - Meditation			Podcast - PMR			Meditation - PMR		
	EMM (*SE*)	95% CI		EMM (*SE*)	95% CI		EMM (*SE*)	95% CI	
		*LL*	*UL*		*LL*	*UL*		*LL*	*UL*
Delta Stereotypic	-0.22 (0.17)	-0.629	0.189	0.29 (0.17)	-0.119	0.704	0.51 (0.16)**	0.117	0.908
Delta White unarmed	0.70 (0.14)***	0.352	1.051	0.25 (0.14)	-0.106	0.598	-0.46 (0.14)**	-0.794	-0.117
Delta White armed	0.48 (0.10)***	0.235	0.719	-0.15 (0.10)	-0.395	0.092	-0.63 (0.10)***	-0.862	-0.395
Delta Black unarmed	0.66 (0.14)***	0.335	0.994	0.04 (0.14)	-0.295	0.369	-0.63 (0.13)***	-0.95	-0.31
Delta Black armed	0.22 (0.11)	-0.035	0.476	-0.07 (0.11)	-0.324	0.189	-0.29 (0.10)	-0.535	-0.042
Alpha Difference^a^	0.04 (0.04)	-0.050	0.138	-0.03 (0.04)	-0.123	0.067	-0.07 (0.04)	-0.163	0.019
Alpha White	-0.08 (0.03)**	-0.138	-0.013	-0.03 (0.03)	-0.093	0.034	0.05 (0.02)	-0.015	0.107
Alpha Black^a^	-0.03 (0.04)	-0.120	0.057	-0.06 (0.04)	-0.146	0.032	-0.03 (0.04)	-0.111	0.060

Note. Estimated Marginal Mean Contrasts (t1 - t0) were used to test difference in change from pre- to post-measurement between conditions. Degrees of freedom method: Kenward-Roger. P-value adjustment: Tukey method for comparing a family of 3 estimates. ^a^Computed from non-significant interactions. *p ≤ .0.5. **p ≤ 0.01. ***p ≤ 0.001.

**Fig 4:**
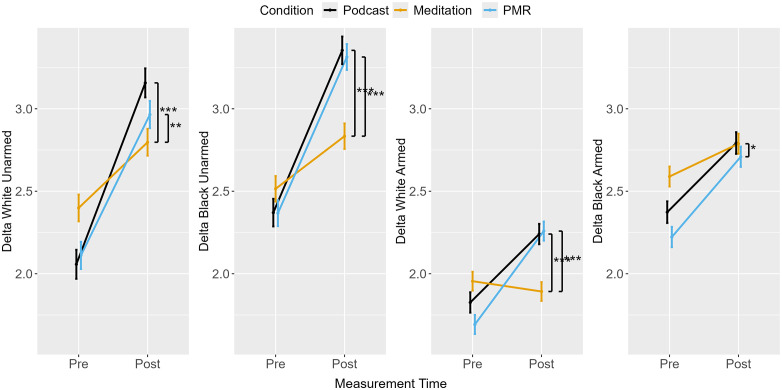
Shooter Task: Drift Rates from Pre- to Post-Measurement.

The separate ANOVAs for all combinations of race and object revealed a main effect for measurement point and an interaction between measurement point and condition (see Table 3, Fig 4). Across conditions, evidence accumulation generally improved from pre- to post-measurement, with larger improvements following podcast listening and PMR than after breathing meditation. Importantly, the increase in stereotypic drift rate following breathing meditation was driven by a lack of improvement in stereotype-incongruent trials rather than an increase in stereotype-congruent processing. In contrast, PMR produced relatively larger improvements in stereotype-incongruent trials, resulting in a reduction in stereotypic drift rate. However, planned comparisons between breathing meditation and podcast listening and between PMR and podcast listening were not significant (Table 4).

### Threshold separation (alpha)

**Fig 5:**
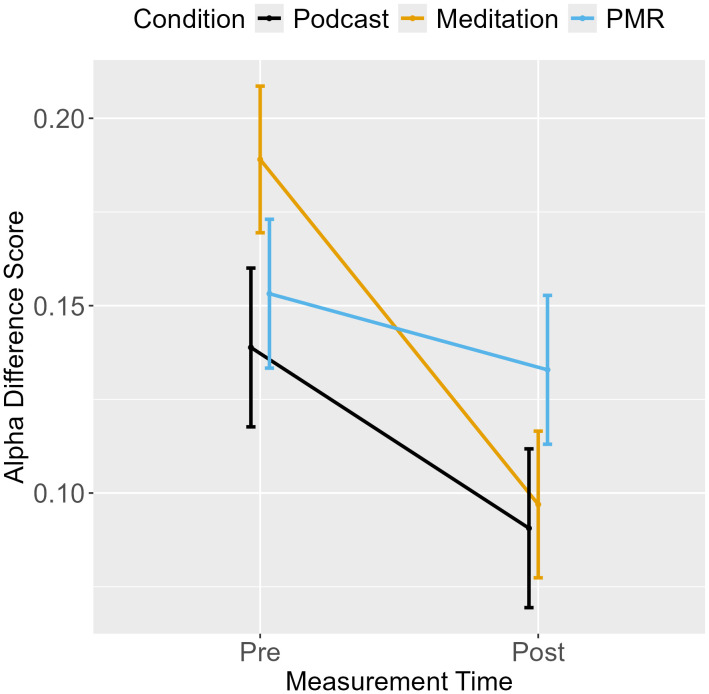
Shooter Task: Difference Score for Threshold Separation from Pre- to Post-Measurement.

An ANOVA for the difference score (Alpha_Black_ – Alpha_White_) revealed a main effect of measurement point but no interaction between measurement point and condition (see Table 3), indicating that as participants decreased controlled responding from pre- to post-measurement, the effect of target race on threshold separation also decreased (see Fig 5). As the interaction was not significant, planned comparisons between conditions were not interpreted.

**Fig 6:**
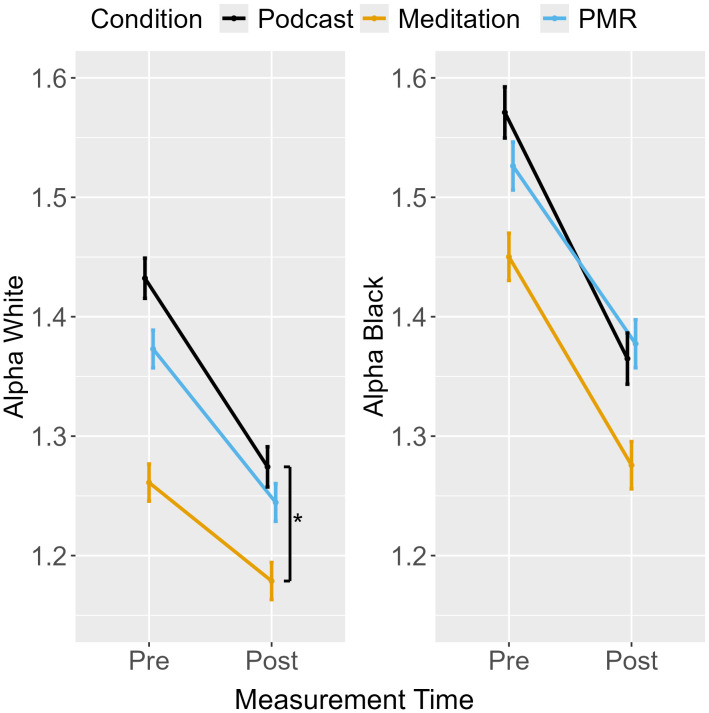
Shooter Task: Threshold Separation from Pre- to Post-Measurement.

Separate ANOVAs were performed for alpha in White and Black trials. For White targets, a main effect for measurement point and an interaction between measurement point and condition was found. Planned comparisons show that podcast listening decreased threshold separation to a greater extent than breathing meditation, but no further comparisons reached significance (see Table 4). The ANOVA for Black targets revealed a main effect for measurement point only, indicating reduced threshold separation from pre- to post-measurement across conditions. EMMs are displayed in Fig 6. Overall, the decrease in alpha indicates faster but more error-prone responding over time.

## Results Avoidance Task

Three participants needed to be removed from the analysis of the Avoidance Task due to failure to record their data properly. Prior to modeling, reaction times smaller than 100 ms were removed, resulting in 0.91% data loss. Again, the results will focus on effects of time and interactions between time and condition for the parameters delta (drift rate) and alpha (threshold separation). Results for beta and tau can be found in S2 Appendix; in short, analyses of the beta (initial bias) and tau (non-decision time) results indicated no effect of target ethnicity on initial bias or non-decision time in the Avoidance Task.

### Positive and negative affect

As in Experiment 1, the interaction between measurement point and condition did not reach significance for PANAS scores, see Table 5. Accordingly, analyses did not include covariates controlling for affective states.

**Table 5:** Avoidance Task: Significance Testing for Fixed Effects for the PANAS Scales utilizing Analysis of Variance with Kenward-Rogers correction.

	Sum of Squares	df	Mean Square	*F*	*p*
**PANAS Positive Affect**					
Measurement Point	0.05	1, 66	0.05	0.16	0.693
Condition	0.20	2, 66	0.10	0.33	0.723
Measurement Point x Condition	0.12	2, 66	0.06	0.20	0.821
**PANAS Negative Affect**					
Measurement Point	0.41	1, 66	0.41	3.31	0.073
Condition	0.24	2, 66	0.12	0.97	0.386
Measurement Point x Condition	0.12	2, 66	0.06	0.48	0.622

### Motivation to control prejudice

As results of Experiment 1 indicated a reduction in controlled responding for all conditions from pre- to post-measurement and previous studies have indicated a relationship between motivation to control prejudice and exertion of cognitive control over stereotype-biased responses [e.g., 23], participants’ motivation to control prejudiced behavior was assessed at pre-measurement. A Spearman rank correlation was performed between the motivation to control prejudice score and threshold separations, drift rates and their respective difference scores at pre-measurement. However, no significant relationship was found, all p’s > 0.05, suggesting that motivation to control prejudice did not affect threshold separation or evidence accumulation during the Avoidance Task. Accordingly, analyses reported below did not include covariates controlling for motivation to control prejudice.

### Drift rate (delta)

**Fig 7:**
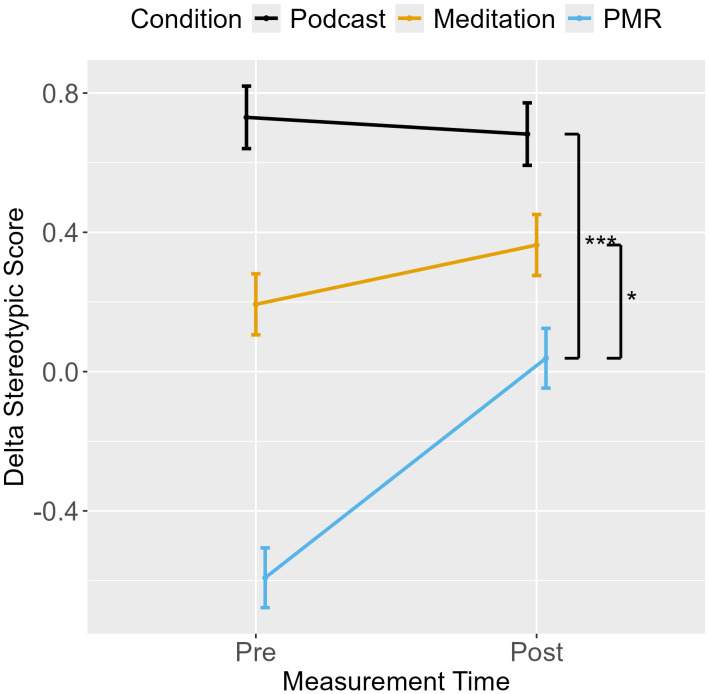
Avoidance Task: Stereotypic Drift Rate from Pre- to Post-Measurement.

An ANOVA for stereotypic drift rate revealed a main effect for measurement point and an interaction between measurement point and condition (see Table 6). Planned comparisons are reported in Table 7. Results revealed that PMR showed a counter-stereotypic bias (i.e., more efficient evidence accumulation for incongruent than congruent trials) at pre-measurement and exhibited no bias at post-measurement (see Fig 7). In comparison, breathing meditation exhibited an increase in stereotype bias, and podcast listening exhibited the highest effect of stereotype congruency on evidence accumulation but no pre-post change.

**Table 6:** Experiment 2: Mixed ANOVAs for the DDM Parameters Delta and Alpha in the Avoidance Task.

	Sum of Squares	df	Mean Square	*F*	*p*
**Delta Stereotypic**					
Measurement point	2.07	1, 63	2.07	13.66	**<.001**
Condition	17.43	2, 63	8.72	57.43	**<.001**
Measurement point x Condition	2.66	2, 63	1.33	8.77	**<.001**
**Delta German unarmed**					
Measurement point	6.04	1, 63	6.04	75.52	**<.001**
Condition	1.82	2, 63	0.91	11.40	**<.001**
Measurement point x Condition	2.90	2, 63	1.45	18.11	**<.001**
**Delta Turkish unarmed**					
Measurement point	2.08	1, 63	2.08	24.37	**<.001**
Condition	0.29	2, 63	0.15	1.73	0.186
Measurement point x Condition	10.99	2, 63	5.50	64.46	**<.001**
**Delta German armed**					
Measurement point	1.36	1, 63	1.36	19.80	**<.001**
Condition	0.82	2, 63	0.41	5.98	**0.004**
Measurement point x Condition	0.30	2, 63	0.15	2.16	0.123
**Delta Turkish armed**					
Measurement point	2.53	1, 63	2.53	25.14	**<.001**
Condition	0.28	2, 63	0.14	1.38	0.260
Measurement point x Condition	1.42	2, 63	0.71	7.08	**0.002**
**Alpha Difference Score**					
Measurement point	0.01	1, 63	0.01	1.08	0.304
Condition	0.02	2, 63	0.01	1.14	0.326
Measurement point x Condition	0.09	2, 63	0.04	5.20	**0.008**
**Alpha German**					
Measurement point	0.00	1, 63	0.00	0.48	0.490
Condition	0.01	2, 63	0.00	0.43	0.655
Measurement point x Condition	0.08	2, 63	0.04	4.85	**0.011**
**Alpha Turkish**					
Measurement point	0.00	1, 63	0.00	0.31	0.582
Condition	0.01	2, 63	0.01	1.96	0.150
Measurement point x Condition	0.00	2, 63	0.00	0.03	0.971

**Table 7:** Experiment 2: Planned Comparisons for Evidence Accumulation (Delta) and Threshold Separation (Alpha).

	Podcast - Meditation			Podcast - PMR			Meditation - PMR		
	EMM (*SE*)	95% CI		EMM (*SE*)	95% CI		EMM (*SE*)	95% CI	
		*LL*	*UL*		*LL*	*UL*		*LL*	*UL*
Delta Stereotypic	-0.22 (0.17)	-0.632	0.195	-0.68 (0.17)***	-1.088	-0.270	-0.46 (0.16)*	-0.864	-0.056
Delta German unarmed	-0.58 (0.12)***	-0.883	-0.283	0.08 (0.12)	-0.217	0.377	0.66 (0.12)***	0.370	0.957
Delta German armed^a^	0.16 (0.11)	-0.120	0.437	-0.07 (0.11)	-0.341	0.209	-0.22 (0.11)	-0.497	0.047
Delta Turkish unarmed	-0.98 (0.13)***	-1.286	-0.666	0.39 (0.13)	0.079	0.693	1.36 (0.12)***	1.059	1.665
Delta Turkish armed	-0.45 (0.14)**	-0.790	-0.117	-0.44 (0.14)**	-0.772	-0.106	0.01 (0.13)	-0.315	0.343
Alpha Difference	0.10 (0.04)*	-0.001	0.194	0.12 (0.04)**	0.024	0.217	0.02 (0.04)	-0.071	0.120
Alpha German	-0.09 (0.04)*	-0.189	0.004	-0.11 (0.04)**	-0.210	-0.019	-0.02 (0.04)	-0.116	0.072
Alpha Turkish^a^	0.00 (0.03)	-0.059	0.066	0.01 (0.03)	-0.056	0.068	0.00 (0.02)	-0.058	0.063

Note. Estimated Marginal Mean Contrasts (t1 - t0) were used to test difference in change from pre- to post-measurement between conditions. Degrees of freedom method: Kenward-Roger. P-value adjustment: Tukey method for comparing a family of 3 estimates. ^a^Computed from non-significant interactions. *p ≤ .0.5. **p ≤ 0.01. ***p ≤ 0.001

**Fig 8:**
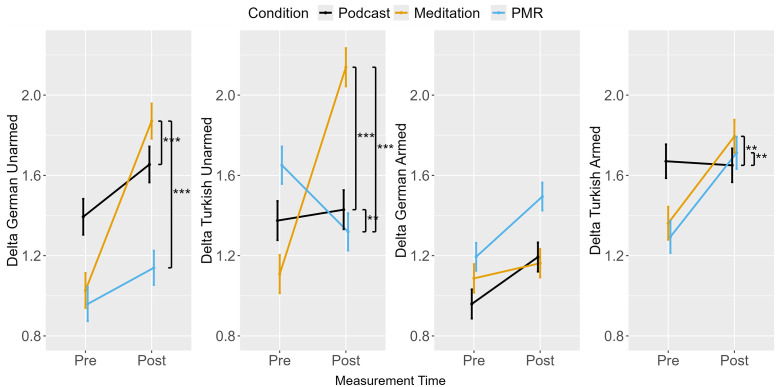
Avoidance Task: Drift Rates from Pre- to Post-Measurement.

**Fig 9:**
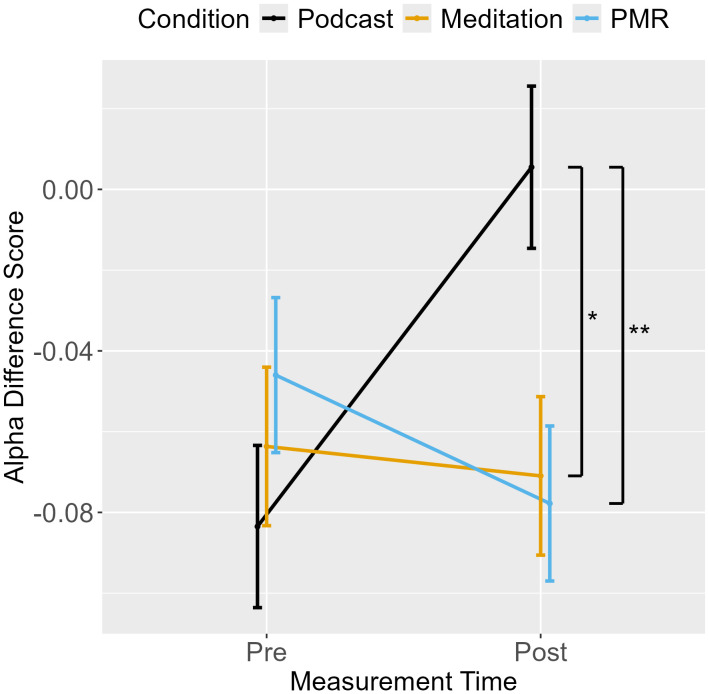
Avoidance Task: Difference Score for Threshold Separation from Pre- to Post-Measurement.

Separate ANOVAs for German and Turkish unarmed as well as armed targets were performed. Evidence accumulation generally increased from pre- to post-measurement across conditions, although the pattern differed by condition (see Fig 8). Similar to Experiment 1, breathing meditation produced the smallest improvement in stereotype-incongruent trials (German armed), which contributed to the observed increase in stereotypic drift rate (Fig 9). Furthermore, reduced evidence accumulation in stereotype-incongruent unarmed trials following PMR led to a reduction in the effect of ethnicity on evidence accumulation.

### Threshold separation (alpha)

An ANOVA for the alpha difference score revealed an interaction between measurement point and condition (see Table 6). Planned contrasts between conditions showed that podcast listening decreased the difference in threshold separation for German and Turkish targets compared to breathing meditation and PMR, which were followed by similar increases (Table 7, Fig 9). However, the difference in threshold separation between German and Turkish targets was minor for all conditions, indicating that, compared to the Shooter Task in Experiment 1, target ethnicity did not affect response caution substantially (i.e., EMMs for the alpha difference score for all conditions at pre- and post-measurement ranged between −0.08 and 0.01, SE = 0.02; see Fig 9).

**Fig 10:**
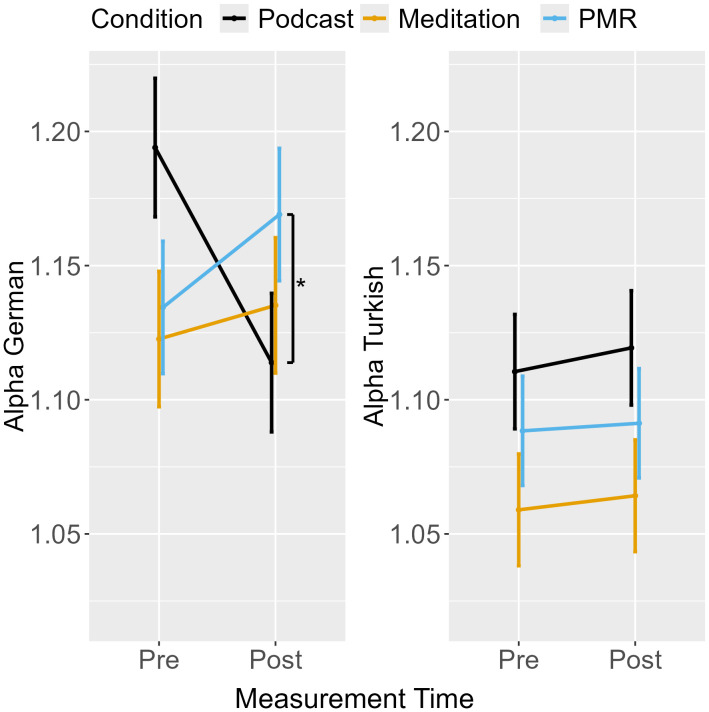
Avoidance Task: Threshold Separation from Pre- to Post-Measurement.

Separate analyses were performed for alpha in German and Turkish trials (Table 6, Fig 10). For German targets, the ANOVA revealed an interaction between measurement point and condition. Planned contrasts indicated that podcast listening decreased threshold separation from pre- to post-measurement, whereas breathing meditation and PMR were associated with increases in threshold separation (Table 7). For Turkish targets, no main effects or interactions were observed, indicating stable response caution across measurement points.

## Discussion

Social-cognitive models of stereotyping have proposed that while stereotypes and related motor responses are activated automatically and unintentionally, initiating non-prejudiced behavior requires the detection of a conflict between the activated stereotype, situational cues and the current goal set, as well as the subsequent activation of cognitive control to inhibit biased behavioral tendencies and to initiate alternative, unbiased responses [7–9,17]. Correspondingly, studies have shown the involvement of conflict monitoring and detection and executive functioning in successful behavioral suppression of activated stereotypes [e.g., 21, 22–24]. The investigation of decision-making processes underlying stereotype expression in the Shooter Task has shown that stereotype bias can affect information processing in the following ways [44–46]: evidence for a correct response accumulates more efficiently in stereotype-congruent compared to incongruent trials, leading to accelerated response speed and heightened accuracy. Also, greater controlled responding has been reported for Black compared to White targets, characterized by more accurate but slower responses, which has been interpreted as reflecting a motivation to control stereotype-biased responding. Therefore, the present paper investigated whether training cognitive control, more specifically improving conflict monitoring and resolution, through mindful breathing meditation would lead to less stereotype-biased information processing and more controlled responding in the Shooter and Avoidance Tasks. Previous findings have indicated that even short inductions of a mindful state effectively decrease racial stereotype activation and bias expression [33,34]. However, studies utilizing longer training durations have reported no effects of mindfulness on racial stereotype expression [35].

In Experiment 1, the effects of three practice sessions of mindful breathing meditation (10, 15 and 20 minutes) on the decision process during the Shooter Task were contrasted with the effects of a short relaxation training and a passive podcast listening condition. In Experiment 2, we increased the training length and the number of training sessions (4 x 20 minutes) and utilized the Avoidance Task to address the generalizability of the effects reported for the Shooter Task [e.g., 46] as well as to increase the ecological validity of the scenario for a German sample. Decision behavior was modeled with drift diffusion models, which jointly take into account response times and responses. Results concerning effects on the expression of stereotype bias and decision-making processes will be discussed for both experiments.

We postulated that enhanced conflict monitoring and resolution following mindful breathing meditation would improve the processing of task-relevant information (i.e., object type) while disregarding task-irrelevant information (i.e., target race or ethnicity). Such an improvement would reduce the effect of stereotype bias on evidence accumulation (drift rate; delta) during the Shooter and the Avoidance Task, meaning that evidence accumulation for correct decisions would not be facilitated in stereotype-congruent compared to incongruent trials. For the Shooter Task in Experiment 1, stereotypic drift rates (Delta_stereotypic_) at pre-measurement indicated the presence of a stereotype bias for all conditions, indicating that evidence for a correct decision accumulated more efficiently for stereotype-congruent compared to incongruent trials and thus replicating previous findings that stereotype bias affects the process of information accumulation [43–46]. Contrary to our hypothesis, the effect of stereotype bias on evidence accumulation increased following breathing meditation training, while a decrease was found following relaxation training. However, pre-post changes in both training groups did not differ from those in the passive control condition. For the Avoidance task in Experiment 2, the passive control group and breathing meditation group exhibited stereotype-biased evidence accumulation at pre-measurement. At the same time, the relaxation group displayed a counter-stereotypic bias (i.e., evidence for stereotype-incongruent trials accrued more efficiently compared to congruent trials). Although such effects are rare, similar counter-stereotype bias patterns have been reported and replicated across various domains, including police use-of-force simulations as well as general population studies [99,100], social judgement in academic evaluations [101,102], and immersive virtual simulations [103]. These effects have been attributed to shifting evaluative standards or to overcorrection in decision-making with or without awareness of racial bias. Correlation analyzes of possible effects of motivation to control prejudice at pre-measurement in Experiment 2 did not indicate an influence of motivation on evidence accumulation, suggesting that the observed counter-stereotypic bias may reflect implicit or automatic processes rather than explicit, consciously motivated overcorrection. Future research is needed to better understand the mechanisms underlying such counter-stereotypic bias patterns and to determine the conditions under which they emerge. While for the relaxation group, the effect of target ethnicity on decision-making was thus not in the expected stereotype-conforming direction, this finding nevertheless reflects an interaction between target ethnicity and object type, indicating that the irrelevant information on target ethnicity affected response behavior – albeit in a counter-stereotype direction. Overall, our findings replicate an effect of stereotype bias on decision-making and response behavior during the Avoidance Task [49]. Similar to the Shooter Task in Experiment 1, breathing meditation training was followed by an increased stereotypic drift rate in the Avoidance Task compared to both control conditions. The passive control condition exhibited no pre-post changes in stereotypic drift rate, while relaxation training reduced the counter-stereotypic drift rate (i.e., reduced an effect of target ethnicity on evidence accumulation) from pre- to post-measurement. In summary, the results of both experiments suggest that short trainings in relaxation, but not mindful breathing, were beneficial for reducing the effect of a (counter-) stereotype bias on response behavior in the Shooter and the Avoidance Task.

Interestingly, mindful breathing meditation increased the effect of stereotype bias on evidence accumulation in both tasks due to lesser improvement in stereotype-incongruent (White or German) armed trials. This indicates that breathing meditation was followed by comparably higher ambiguity in evidence accumulation (which would be reflected in slower and more error-prone responses) in trials with heightened response conflict (i.e., target race or ethnicity was stereotypically incongruent with the presence of a harmful object). For PMR, the results of the Shooter Task indicated that a decrease in the effect of stereotype bias on evidence accumulation was based on greater improvements in stereotype incongruent (White) armed targets (reflected in faster and more accurate responding). For the Avoidance Task, PMR displayed a counter-stereotypic bias at pre-measurement and the reduction of this bias was based on a reduction in evidence accumulation in stereotype-incongruent (Turkish) unarmed trials. While a counter-stereotype bias was unexpected, these results still imply that task performance at post-measurement following PMR was less dependent on associations between target ethnicity and object type. In summary, all reported changes in the effect of stereotype bias were explained by respective changes in incongruent but not congruent trials, suggesting that mindful breathing meditation and PMR differentially affected information processing under conditions that induced response conflict between a pre-potent stereotype-biased response and unbiased response in adherence with task-goals. However, why these effects only emerged in either armed or unarmed incongruent trials is unclear.

For threshold separation (i.e., the amount of evidence required for decision-making; alpha), we proposed that improved cognitive control through breathing meditation should lead to increased controlled responding (i.e., longer reaction times and less error-prone responding) for Black or Turkish targets. In line with previous studies [44,46,47], results for the Shooter Task indicated that all conditions exhibited higher controlled responding for Black compared to White targets at pre-measurement. Differences in threshold separation for Black compared to White targets decreased for all conditions from pre- to post-measurement, indicating that repeated testing reduced the effect of target race on response style across conditions. For the Avoidance Task, effects of target ethnicity on threshold separation were non-substantial. Assessment of possible effects of motivation to control prejudice on decision-making revealed that there was no correlation between threshold separation and self-reported motivation to control prejudice during the Avoidance Task. This finding, as well as the fact that target ethnicity did not affect response style substantially, contrasts with previous interpretations of heightened controlled responding as indicating an effort to control stereotype-biased behavior for Black targets in the Shooter Task [e.g., 46]. In summary, contrary to our hypothesis, we did not find any effect that breathing meditation improves controlled responding in both experiments.

As mentioned in the results section, results for initial bias and non-decision time did not indicate an effect of target race or ethnicity in the Shooter and the Avoidance Task (see S2 Appendix). The results for initial bias showed that conditions overall exhibited a gun detection or avoid bias respectively, indicating response adaptation to the reward manipulations by the pay-off matrices in both tasks. These findings are in line with previous studies [43–46], as well as with theoretical considerations regarding the underlying cognitive processes [46].

The finding of enhanced stereotype bias in information processing following mindful breathing meditation may be explained by that the meditation trainings in both experiments were sufficient in length and dose to increase participants’ conflict monitoring, thereby increasing the salience of race or ethnicity, but were not sufficient to increase the cognitive control necessary to resolve response conflict when confronted with stereotype-incongruent trials. The distinction between conflict monitoring and successful control exertion has been described in current theoretical frameworks and has been linked to different neural systems, with the anterior cingulate cortex (ACC) monitoring response conflict elicited by stimuli and the prefrontal cortex (PFC) regions implementing top-down control [104,105]. Thus, in our study, meditation may have amplified sensitivity to monitoring stimulus-elicited conflict without adequately engaging top-down control processes needed to resolve it, resulting in degraded evidence accumulation specifically in stereotype-incongruent trials. Consistent with this interpretation, results from cognitive tasks utilized in the present experiments to investigate potential benefits of brief meditation trainings on executive functions (reported in full length elsewhere [58]) showed no meditation specific improvements in cognitive inhibition, updating or task switching [for a discussion of dose-response relations for mindfulness meditation training and executive functioning, see [56, 58]. Rather, experimental conditions improved equally from pre- to post-measurement, which indicates effects of repeated measurements but no training-specific benefits. The finding of enhanced response conflict following a short-term meditation training without enhancement of cognitive control is furthermore consistent with the proposition that conflict monitoring in particular develops in early-phase meditation training, as the detection of distractions that are in conflict with the current task-goals is a prerequisite for improving cognitive control necessary for sustaining attention during the meditative practice [e.g., 106]. Further support comes from research demonstrating that even brief meditation interventions can enhance conflict monitoring processes [for a review, see 31]. Neurophysiological studies show increased error-related negativity (ERN), a marker of conflict monitoring, after short mindfulness practice [107,108], and among experienced meditators [109]. Additionally, Larson and colleagues [110] reported reduced amplitude of error positivity (Pe) activity, a marker of error awareness as well as motivational salience, in a Flanker Task following a brief mindfulness meditation compared to a passive control condition, while performance did not improve for the meditation condition compared to the passive control condition [111]. Although we did not include neurophysiological measures, the decreased efficiency in information processing observed in trials with high stereotype conflict may reflect heightened evaluative monitoring or attentional engagement under cognitive uncertainty. We therefore interpret these findings as suggestive, albeit not conclusive, evidence of enhanced conflict monitoring, with the caveat that future studies should include direct neural or behavioral indices of this mechanism. More specifically, our findings showed that meditation training increased response conflict in stereotype-incongruent trials featuring targets from participants’ ethnic in-group (i.e., White or German), suggesting a potential effect of increased conflict monitoring that is particularly pronounced in response to one’s in-group. Related to this, research demonstrates that mindfulness meditation enhances self-awareness of bodily and emotional states [112–114], which may make one’s social identity, and associated group norms, more salient. This heightened self-centered awareness could have intensified the cognitive conflict when stereotype-incongruent behavior was observed within one’s social group, as violations of expected in-group behavior tend to evoke stronger responses [115,116]. While previous research has explored how increased self-awareness from mindfulness may mitigate out-group biases [for a review, see 117], its potential impact on in-group norm awareness and stereotype conflict remains an open area for further study.

Considering the possibility that meditation can be rather effortful for novices in that it requires to actively sustain attention on the task over a prolonged period of time [118], it could be argued that cognitive depletion following meditation training enhanced the effect of stereotype bias on decision-making, while relaxation, with its focus on a guided experience of physical sensations of relaxation, might have freed up cognitive resources necessary for reducing this effect. This idea is theoretically supported by the attentional control theory [119], which states that stress impairs cognitive resources critical for executive control, thereby increasing reliance on automatic responses such as stereotype biases. By reducing stress, relaxation could have restored these cognitive resources, enhancing the ability to regulate automatic stereotype responses. Accordingly, research has shown that relaxation can enhance cognitive performance [120,121], and that lower stress levels can reduce automatic stereotype responses compared to high stress [122]. With regard to the finding of enhanced stereotype expression following meditation training, heightened cognitive load has been found to increase bias expression in a Shooter Task and even diminish practice effects [123]. However, further research from Govorun and Payne [12] showed that depleting cognitive resources leads to reduced controlled responding but not increased automatic bias in a Weapon Identification Task. As both the breathing meditation training as well as the relaxation training in the present experiments did not affect response caution specifically (i.e., all conditions exhibited similar decreases in controlled responding from pre- to post-measurement), this interpretation cannot account for the results presented. Furthermore, cognitive tasks measuring executive functioning in the present experiments did indicate neither an enhancement nor depletion of cognitive control following meditation or relaxation training. Thus, given previous research and our own results, while theoretically plausible, a depletion-based explanation cannot account for the present findings.

As previous research has suggested that meditation may reduce reward processing and reward-seeking tendencies [124–126] and that findings of reduced pro-social behavior following mindfulness meditation training could be attributed to such reductions in reward salience [127,128], one might interpret the effects observed in the present experiments to changes in reward sensitivity. However, our findings showed that participants adapted to the reward contingencies of the Shooter and Avoidance tasks across all conditions, as indicated by the results of initial bias (beta; see S2 Appendix). This adaptation was consistent across both meditation and control groups, suggesting that the breathing meditation training did not uniquely affect reward salience or reward-seeking behavior. Importantly, meditation effects emerged in the drift rate parameter, which reflects the efficiency of evidence accumulation during decision-making. Unlike initial bias (beta), which is sensitive to changes in reward structure and captures pre-decisional response biases, drift rate is thought to reflect the quality of stimulus-based information processing and is generally less influenced by reward contingencies [42]. Therefore, the observed drift-rate differences are unlikely to be driven by reward-seeking behavior and instead suggest changes in how stereotype-relevant information was cognitively processed following meditation training.

Our results of enhanced stereotype bias following a short mindful breathing meditation in both experiments differ from Lueke and Gibson’s [33] findings of reduced automatic stereotype associations on race and age IATs following 10 minutes of breathing meditation and also of their finding of less biased treatment of Black compared to White players in a trust game following meditation [34]. These differential findings may be related to differences in the paradigms utilized. The IAT requires that participants shift between a concept (e.g., ethnicity) and an attribute (e.g., valence) categorization task, whereas the Shooter and Avoidance Tasks do not require such shifts between task sets (i.e., in every trial, the task is to identify the target object). Relatedly, Ito, Friedman [24] showed that for the Shooter Task, greater control over bias expression was predicted by common executive functioning ability, while for the IAT, bias control was predicted specifically by shifting ability [for a discussion of the involvement of task switching in IAT performance, see for example 129]. Furthermore, while both tasks in the present paper imply that a physical threat needs to be avoided (by identifying or avoiding an armed target), the trust game utilized by Lueke and Gibson [34] implies that a monetary gain can be optimized based on the trustworthiness of a counterpart, which does not induce a situation of danger or threat. Thus, with regards to the cognitive processes involved in these distinct paradigms, the Shooter and Avoidance Tasks primarily tax executive control under time pressure, requiring participants to suppress prepotent responses (e.g., “shoot” or “approach”) when faced with stereotype-incongruent stimuli. In contrast, the IAT emphasizes task switching and set-shifting, as participants must frequently re-map category-response associations (e.g., race with good vs. bad) across blocks, engaging cognitive flexibility. Trust games, meanwhile, are less reliant on immediate executive control and instead draw on valuation, expectancy updating, and risk assessment, often in low-arousal contexts [130]. These divergent cognitive demands may account for why mindfulness-based interventions differentially affect bias expression across paradigms, enhancing flexible categorization in some contexts (e.g., IAT), but not consistently reducing automatic responses in high-conflict, fast-response tasks like the Shooter paradigm. Future research may examine the impact of mindful meditation trainings on stereotype bias across diverse task paradigms, such as trust games, IATs, and threat-based tasks and specify how effects of the practice depend on cognitive engagement required by different task demands.

## Limitations and future research

Implementing the experiments in an online research environment required careful checks regarding the fidelity of training delivery, testing and participants’ compliance. As described above, we assured fidelity by providing thorough instructions about necessary equipment and an adequate environment for participation, sound checks prior to audio delivery of trainings and podcast listening and requested that participants agree in writing to adhere to the instructions. Moreover, participants reported the frequency and severity of disturbances. A more detailed description of the results of these checks is reported in Vieth and von Stockhausen [58]. Overall, most participants reported no disturbances during testing and audio delivery, and the disturbances reported were rated to be of low impact on study participation. Therefore, online testing and audio delivery implementation seemed to have been of sufficient quality. We acknowledge that online implementation may have introduced uncontrolled variability in participants’ settings (e.g., environmental surroundings, hardware differences). Replicating the findings under controlled laboratory conditions would help to rule out potential environmental confounds and strengthen internal validity.

To our knowledge, this study is the first to apply drift diffusion modeling to the Avoidance Task. Based on theoretical considerations and for direct comparability of the results of both experiments, we utilized the same HDDMs for the Shooter and the Avoidance Task. The results generally suggested that decision-making processes in the Avoidance Task were well reflected by the parametrization (i.e., an avoidance bias could be replicated; effects on bias expression following breathing meditation and PMR emerged similarly across tasks). Still, assessing how well the models’ parametrization accounts for the data (e.g., posterior predictive checks) in future studies would be advisable. Furthermore, estimating models in which parameters vary differently by trial conditions (e.g., allowing threshold separation to vary as a function of object type) and comparing model fits could be done to further assess the adequacy of the model for the Avoidance Task [for a discussion of variations in parameter estimation for the Shooter Task, see 46].

For the Shooter Task, we decided to instruct participants to indicate the presence or absence of a gun instead of asking participants to make a “shooting decision” as in the original task by Correll, Park [36]. While we thus adapted instructions to improve the external validity of the task in a German sample, this may have affected the comparability of our results to studies utilizing the original instructions. However, since effects of stereotype activation on response behavior can also be found in the Weapon Identification Task [e.g., 131], which similarly instructs participants to identify objects as a tool or a weapon, we would argue that the internal validity of the task was not threatened. In addition, we were able to replicate known task effects despite adapting the instructions. In line with previous research, an effect of stereotype bias was found in enhanced information processing for stereotype-congruent compared to incongruent trials [43–46]. Furthermore, reduced response caution in light of the restricted response window surfaced from pre- to post-measurement [46], and threshold separation was larger for Black compared to White targets [44,46,47]. Supplementary analyses (see S2 Appendix) of initial bias showed that participants exhibited a “gun” bias at pre-measurement [44, 46; but see 43], which indicates that response behavior was manipulated by the pay-off matrix. Additionally, we found shorter non-decision time for gun compared to non-gun trials, which has also been reported in previous studies [43,44,46]. Nevertheless, we acknowledge that our adaptation may have reduced perceived threat or emotional salience, potentially altering motivational or attentional engagement. Future studies may therefore consider directly comparing both task versions to assess differences in cognitive processing or affective load introduced by such adaptations.

The present experiments were parts of two studies that also assessed the executive functions of inhibition, updating/working memory and task switching. The analyses of the effects of short mindful breathing meditation trainings on separate executive functions did not reveal significant improvements [58]. However, there is evidence that while mindfulness meditation trainings may not lead to domain-specific improvements in executive functions, overall executive control abilities across separate domains are improved [132,133], and common executive ability has been shown to reduce stereotype bias expression [24]. Future research may implement designs suitable for latent variable or mediation analysis to assess whether short mindfulness meditation trainings may lead to domain-general improvements in EFs or, relatedly, whether changes in specific executive components serve as a mediating mechanism in reducing stereotype bias in response behavior..

While the present experiments utilized pre-post designs to investigate the effect of short mindful meditation training on stereotype expression, future studies may employ designs suitable for investigating the dose-response relationship of meditation on stereotype biases in depth (e.g., longitudinal designs with repeated testing). Such investigations would enhance our understanding on when effects of meditation surface reliably. Additionally, while even short durations of mindfulness meditation have been shown to reduce stereotype activation and bias expression [33,34], longer training durations might be necessary to observe reliable improvements in cognitive control and a subsequently decreased effect of stereotype bias on decision making [56,57].

Although participants with recent meditation experience were excluded from the study, prior experience beyond this timeframe was recorded. As reported elsewhere [58], such prior experience did not influence performance on cognitive tasks. Nonetheless, the potential moderating role of long-term meditation practice remains an open topic for future research. Studies comparing novice and experienced practitioners may help clarify whether intervention effects on stereotype expression vary as a function of prior exposure or accumulated practice.

Participants were primarily recruited from a university setting via internal message boards and social media, resulting in a predominantly young, female, and German sample, which may limit the generalizability of the findings to broader populations. However, participants were randomly assigned to conditions using a double-blind protocol and were unaware of the study’s goals, reducing the risk of expectancy or self-selection effects. While this sampling approach is common in experimental research, future studies should aim to replicate these findings in more demographically and culturally diverse populations.

In Experiment 2, a small number of participants (approximately 1–2%) identified as members of the stereotyped target group (e.g., of Turkish or Kurdish descent). Prior research has shown that stereotype expression can occur even within in-groups, due to the internalization of culturally shared biases [7]. All participants were university students living in Germany and are thus likely to have been exposed to, and influenced by, the same dominant cultural stereotypes, regardless of their ethnic background. We therefore consider it unlikely that participants’ ethnic origin significantly confounded the observed effects, especially since only a small number of participants identified as members of the stereotyped target group. Nonetheless, future studies could seek to systematically counterbalance participant ethnicity across conditions or include ethnic background as a covariate or moderator in modeling approaches, to explore whether in-group status influences the processing of stereotype-congruent or incongruent information.

## Conclusion

We found an increase in the effect of stereotype bias on evidence accumulation following short trainings in mindful breathing meditation in the Shooter Task (Experiment 1) and the Avoidance Task (Experiment 2). Unexpectedly, short trainings in relaxation resulted in reduced effects of stereotype bias on information processing in both experiments. Based on the involvement of cognitive control in the suppression of stereotype-biased behavior [e.g., 17, 20, 23], beneficial effects of mindfulness meditation on conflict detection and resolution [31,32], and Lueke and Gibson’s findings [33,34], we would have expected to find improvements in unbiased decision-making and responding following meditation training. The increase in stereotype bias following breathing meditation was based on heightened response conflict in stereotype-incongruent trials but did not reflect facilitated information processing in stereotype-congruent trials. While the present study cannot offer a definitive explanation for these findings, we have proposed that improved conflict monitoring combined with heightened self-awareness following breathing meditation training may have amplified the salience of participants’ own social identity. A heightened self-focus may evoke stronger conflict when confronted with stereotype-incongruent behavior within the in-group, potentially explaining the observed increase in response conflict [112–114]. This is further supported by theoretical accounts proposing that conflict monitoring develops in early phases of meditation training, while reliable effects on cognitive control require longer training regimes [106]. Simultaneously, the dosage of practice may have been too small to increase the executive functioning necessary to suppress the effect of task-irrelevant information in stereotype-incongruent trials and to resolve the resulting response conflict. This is supported by findings from additional tasks measuring executive functioning, conducted in both experiments, and reported in detail elsewhere [58]. Still, we would not have expected to find an increased effect of stereotype bias in the absence of beneficial effects after meditation (or improved suppression of stereotype-biased behavior following induced relaxation). These findings suggest that short-term interventions of mindfulness meditation may temporarily amplify stereotype bias through heightened self-focus and response conflict, while relaxation training unexpectedly mitigated such biases. This highlights the need for future research to disentangle the temporal development of such meditation-related effects. Further training or additional interventions may be required to translate this enhanced monitoring into improved control over biased responses. Studies that directly measure conflict sensitivity, self-related processing, and cognitive control across different phases of meditation training will be critical to understanding under which conditions mindfulness meditation can reduce stereotype expression.

## Supporting information

S1 Appendix Instructions for the Breathing Meditation and Progressive Muscle Relaxation. (PDF)

S2 Appendix Results for Beta and Tau. (PDF)

S3 Appendix Information regarding the usage of generative AI. (PDF)

S4 Appendix Model Diagnostics for Drift Diffusion Models. (PDF)
