# Supplementary material for: Effects of mindful breathing meditation on stereotype expression in two randomized controlled double-blinded trials
Source: PLoS One. 2026 Apr 30;21(4):e0347871. doi: 10.1371/journal.pone.0347871 (PMC13132222; doi:10.1371/journal.pone.0347871)
Supplement: S2 Appendix — (PDF) [file pone.0347871.s002.pdf]

S2 Appendix: Results for Beta and Tau

Following previous findings, no effects of target race or stereotype bias on beta or tau were postulated. However, few studies have investigated the effects of repeated testing or interventions on decision-making during the Shooter or Avoidance Task. Therefore, we decided to investigate possible changes related to stereotypes in the respective parameters in a supplementary analysis.

To investigate the effect of target race on initial bias, a difference score ($Beta_{Black}$ – $Beta_{White}$) was calculated to assess differences in the processes by target race/ethnicity, with positive values reflecting greater shotgun detection/avoid bias for Black/Turkish compared to White/German targets. For the assessment of stereotype bias in non-decision time ($Tau_{stereotypic}$), we utilized the calculation of difference scores from [1] (e.g., for the Shooter Task, $Tau_{stereotypic}$ = [$Tau_{Black\ armed}$ – $Tau_{White\ armed}$] – [$Tau_{Black\ unarmed}$ – $Tau_{White\ unarmed}$]). Positive values indicate prolonged tau for stereotype congruent compared to incongruent trials. Furthermore, separate analyses for all target conditions will be provided.

1 Results for the Shooter Task

Analysis of significant pre-post changes (i.e., effects of measurement point) and interaction effects (measurement point x condition) were assessed with ANOVAs (see Table 1). Planned contrasts are portrait in Table 2.

1.1 Initial Bias

In line with previous studies [e.g., 2], there was no substantial effect of race. Generally, conditions increased “gun” biases from pre- to post-measurement, and thus showed overall adaptation to response rewards by the pay-off matrix [2, 3, but see 1], except for the breathing meditation condition exhibiting a decrease in initial bias for Black targets.

1.2 Non-Decision Time

Again, and in line with previous studies, there was no substantial effect of stereotype bias from pre- to post-measurement [1-3]. Furthermore, non-decision time increased from pre- to post-measurement in all trial conditions, albeit to different degrees. Because non-decision time reflects several processes (e.g., stimulus encoding and motor-response preparation), and stereotype bias was of marginal influence, a clear-cut interpretation of these results is not possible based on the current data.

Table 1. Experiment 1: Mixed ANOVAs for the DDM Parameters Beta and Tau in the Shooter Task.

	Sum of Squares	df	Mean Sqare	*F*	*p*
**Beta Difference Score**					
Time of Measurement	0.02	1,93	0.02	7.50	**0.007**
Condition	0.00	2,93	0.00	0.74	0.482
Measurement Point x Condition	0.09	2,93	0.04	16.32	**<.001**
**Beta White**					
Time of Measurement	0.03	1,93	0.03	20.68	**0.016**
Condition	0.03	2,93	0.01	8.80	**<.001**
Measurement Point x Condition	0.02	2,93	0.01	5.44	**0.006**
**Beta Black**					
Time of Measurement	0.00	1,93	0.00	0.89	0.347
Condition	0.01	2,93	0.01	4.08	**0.020**
Measurement Point x Condition	0.06	2,93	0.03	24.93	**<.001**
**Tau Stereotypic**					
Time of Measurement	0.00	1,93	0.00	0.65	0.421
Condition	0.12	2,93	0.06	11.39	**0.038**
Measurement Point x Condition	0.12	2,93	0.06	11.78	**0.028**
**Tau White unarmed**					
Time of Measurement	0.25	1,93	0.25	86.39	**<.001**
Condition	0.09	2,93	0.05	16.23	**<.001**
Measurement Point x Condition	0.09	2,93	0.04	14.76	**0.003**
**Tau Black unarmed**					
Time of Measurement	0.32	1,93	0.32	99.86	**<.001**
Condition	0.02	2,93	0.01	3.73	**0.028**
Measurement Point x Condition	0.03	2,93	0.02	5.47	**0.006**
**Tau White armed**					
Time of Measurement	0.16	1,93	0.16	65.69	**<.001**
Condition	0.01	2,93	0.01	2.60	0.080
Measurement Point x Condition	0.06	2,93	0.03	12.44	**0.016**
**Tau Black armed**					
Time of Measurement	0.17	1,93	0.17	80.83	**<.001**
Condition	0.03	2,93	0.01	6.56	**0.002**
Measurement Point x Condition	0.08	2,93	0.04	19.81	**<.001**

Table 2. Experiment 1: Planned Comparisons for Initial Bias (Beta) and Non-decision time (Tau).

	Podcast - Meditation					Podcast - PMR					Meditation - PMR				
	Estimate	*SE*	*p* value	95% CI		Estimate	*SE*	*p* value	95% CI		Estimate	*SE*	*p* value	95% CI	
				LL	*UL*				*LL*	*UL*				*LL*	*UL*
Beta Difference Score	0.03	0.02	0.148	-0.010	0.080	-0.07	0.02	**0.002**	-0.111	-0.021	-0.10	0.02	**<.001**	-0.144	-0.057
Beta White	0.03	0.01	0.118	-0.006	0.061	0.05	0.01	**0.004**	0.012	0.079	0.02	0.01	0.371	-0.014	0.050
Beta Black	0.06	0.01	**<.001**	0.032	0.093	-0.02	0.01	0.247	-0.051	0.011	-0.08	0.01	**<.001**	-0.112	-0.053
Tau Difference Score	0.05	0.03	0.150	-0.014	0.111	-0.07	0.03	**0.018**	-0.135	-0.009	-0.12	0.02	**<.001**	-0.181	-0.060
Tau White unarmed	0.10	0.02	**<.001**	0.055	0.149	0.04	0.02	0.133	-0.010	0.085	-0.06	0.02	**0.002**	-0.110	-0.019
Tau Black unarmed	0.06	0.02	**0.008**	0.012	0.111	0.06	0.02	**0.021**	0.006	0.105	-0.01	0.02	0.947	-0.054	0.042
Tau White armed	0.09	0.02	**<.001**	0.042	0.129	0.07	0.02	**0.001**	0.023	0.111	-0.02	0.02	0.534	-0.061	0.024
Tau Black armed	0.09	0.02	**<.001**	0.053	0.133	0.01	0.02	0.708	-0.027	0.053	-0.08	0.02	**<.001**	-0.119	-0.042

Note. Estimated Marginal Mean Contrasts (t1 - t0) were used to test difference in change from pre- to post-measurement between conditions. P-value adjustment: Tukey method for comparing a family of 3 estimates.

2 Results for the Avoidance Task

Table 3 depicts the results of ANOVAs for the assessment of pre-post changes (i.e., effects of measurement point) and interaction effects (measurement point x condition). Planned contrasts can be found in Table 4.

2.1 Initial Bias

Similar to the Shooter Task and previous studies, there was no substantial effect of target ethnicity. Generally, all conditions exhibited “avoid” biases for German and Turkish targets at pre- and post-measurement (except for an unbiased response behavior for podcast listening for Turkish targets), indicating overall adaptation to reward manipulation by the pay-off matrix.

2.2 Non-decision Time

Similar to the Shooter Task, effects of stereotype bias on non-decision time were not substantial. However, while in the Shooter Task there were general increases in non-decision time across trial as well as experimental conditions, results for the Avoidance Task were less clear-cut without an interpretable pattern within as well as between experimental or trial conditions. Furthermore, non-decision times were overall longer in the Avoidance than in the Shooter Task. This may be due to the more complex response instructions for the Avoidance Task (i.e., a button press does not just indicate the presence/absence of a weapon but also a spatial avoid or approach response).

Table 3. Experiment 2: Mixed ANOVAs for the DDM Parameters Beta and Tau in the Avoidance Task.

	Sum of Squares	df	Mean Sqare	*F*	*p*
**Beta Difference Score**					
Time of Measurement	0.00	1,63	0.00	1.19	0.279
Condition	0.13	2,63	0.06	67.11	**<.001**
Measurement Point x Condition	0.10	2,63	0.05	52.54	**<.001**
**Beta German**					
Time of Measurement	0.00	1,63	0.00	5.31	**0.025**
Condition	0.01	2,63	0.01	11.70	**<.001**
Measurement Point x Condition	0.01	2,63	0.01	11.29	**<.001**
**Beta Turkish**					
Time of Measurement	0.01	1,63	0.01	13.88	**<.001**
Condition	0.17	2,63	0.09	141.86	**<.001**
Measurement Point x Condition	0.13	2,63	0.07	111.44	**<.001**
**Tau Stereotypic**					
Time of Measurement	0.16	1,63	0.16	36.13	**<.001**
Condition	0.01	2,63	0.00	0.65	0.525
Measurement Point x Condition	0.08	2,63	0.04	9.28	**<.001**
**Tau German unarmed**					
Time of Measurement	0.05	1,63	0.05	11.13	**0.001**
Condition	0.02	2,63	0.01	1.58	0.215
Measurement Point x Condition	0.06	2,63	0.03	6.28	**0.003**
**Tau Turkish unarmed**					
Time of Measurement	0.01	1,63	0.01	7.06	**0.010**
Condition	0.00	2,63	0.00	0.31	0.735
Measurement Point x Condition	0.03	2,63	0.02	11.93	**<.001**
**Tau German armed**					
Time of Measurement	0.01	1,63	0.01	2.36	0.130
Condition	0.01	2,63	0.01	1.70	0.190
Measurement Point x Condition	0.00	2,63	0.00	0.01	0.992
**Tau Turkish armed**					
Time of Measurement	0.00	1,63	0.00	0.16	0.690
Condition	0.02	2,63	0.01	3.24	**0.046**
Measurement Point x Condition	0.04	2,63	0.02	7.72	**0.001**

Table 4. Experiment 2: Planned Comparisons for Initial Bias (Beta) and Non-decision time (Tau).

	Podcast - Meditation					Podcast - PMR					Meditation - PMR				
	Estimate	*SE*	*p* value	95% CI		Estimate	*SE*	*p* value	95% CI		Estimate	*SE*	*p* value	95% CI	
				LL	*UL*				*LL*	*UL*				*LL*	*UL*
Beta Difference	-0.02	0.01	0.364	-0.051	0.014	0.10	0.01	**<.001**	0.072	0.137	0.12	0.01	**<.001**	0.091	0.154
Beta German	-0.05	0.01	**<.001**	-0.078	-0.024	-0.02	0.01	0.181	-0.046	0.007	0.03	0.01	**0.011**	0.006	0.058
Beta Turkish	-0.07	0.01	**<.001**	-0.095	-0.043	0.09	0.01	**<.001**	0.059	0.111	0.15	0.01	**<.001**	0.129	0.180
Tau Difference	0.00	0.03	0.997	-0.069	0.073	0.11	0.03	**0.001**	0.036	0.177	0.10	0.03	**0.001**	0.035	0.174
Tau German unarmed	0.01	0.03	0.922	-0.063	0.086	0.10	0.03	**0.006**	0.023	0.170	0.08	0.03	**0.016**	0.012	0.158
Tau Turkish unarmed	-0.07	0.02	**0.000**	-0.109	-0.033	-0.01	0.02	0.678	-0.051	0.025	0.06	0.02	**0.001**	0.021	0.096
Tau German armed[a]	0.00	0.03	0.991	-0.061	0.067	0.00	0.03	0.997	-0.062	0.065	0.00	0.03	0.998	-0.064	0.061
Tau Turkish armed	-0.08	0.02	**0.003**	-0.133	-0.021	0.00	0.02	0.999	-0.057	0.054	0.08	0.02	**0.003**	0.021	0.131

Note. Estimated Marginal Mean Contrasts (t1 - t0) were used to test difference in change from pre- to post-measurement between conditions. P-value adjustment: Tukey method for comparing a family of 3 estimates. [a]Computed from non-significant interactions.

3 References

1. Correll J, Wittenbrink B, Crawford MT, Sadler MS. Stereotypic vision: how stereotypes disambiguate visual stimuli. *Journal of Personality and Social Psychology*. 2015;108(2):219-33.
2. Pleskac TJ, Cesario J, Johnson DJ. How race affects evidence accumulation during the decision to shoot. Psychonomic Bulletin & Review. 2018;25(4):1301-30.
3. Johnson DJ, Cesario J, Pleskac TJ. How prior information and police experience impact decisions to shoot. *Journal of Personality and Social Psychology*. 2018;115(4):601-23.
