# Supplementary material for: Effects of mindful breathing meditation on stereotype expression in two randomized controlled double-blinded trials
Source: PLoS One. 2026 Apr 30;21(4):e0347871. doi: 10.1371/journal.pone.0347871 (PMC13132222; doi:10.1371/journal.pone.0347871)
Supplement: S3 Appendix — (PDF) [file pone.0347871.s003.pdf]

S3 Appendix: Information regarding the usage of generative AI

To illustrate the stimuli of the Shooter Task [1] and Avoidance Task [2], we used generative AI to visualize the depicted persons in Figure 1 (A,B,C) as well as background images for Figure 1B. The latent text-to-image diffusion model Stable Diffusion [3] was used, which is open-source and licensed under a CreativeML Openrail-M license. Stable Diffusion was accessed via getimg.ai (https://getimg.ai/).

1 AI prompts for Figure 1A

Background images for Figure 1A were kindly provided by Joshua Correll and are part of the original stimulus set [1]. The persons depicted were created using generative AI and subsequently imposed onto the background images. Quality was assessed based on how closely the generated images resembled the original stimuli.

1.1 Prompt for depicted target on the upper panel of Figure 1A

"A young black man standing in front of a black background. He is wearing a white long-sleeved shirt, blue jeans, and white sneakers. He has a serious expression on his face and is holding a gun in his right hand, which is placed across his left shoulder. The scene is realistically full-body portrait image, showing the man standing in a relaxed pose."

1.2 Prompt for depicted target on the lower panel of Figure 1A

"A White Man kneeling on the ground, holding a black phone in his hand. He is wearing a black long-sleeved shirt, blue jeans, and brown shoes. The man is wearing a black sweater, blue jeans, and black shoes. he has a serious expression on his face and is looking directly at the camera."

2 AI prompts for Figure 1B

For the background images in the upper and lower panel of Figure 1B, the same prompt was used. The quality of the results was assessed based on how closely the generated images resembled the original background images of the Avoidance Task, as well as on whether targets could be conveniently placed at the desired positions (e.g., whether the background would obscure the visibility of the target and if the positioning appeared natural).

"Street view of a residential area in Germany. On both sides are walkways for pedestrians, on the left side of the street, there are trees and bushes. The street is empty, with no cars or people visible. The sky is blue and there are a few clouds in the sky. The houses on the right side are white and have a sloping roof. There are no visible powerlines."

2.1 Prompt for depicted target on the upper panel of Figure 1B

"A full-body image from head to toe of a young man with distinct German features and tousled blonde hair, standing on a residential sidewalk. He wears a green t-shirt and well-fitted, slightly distressed jeans on his legs and brown shoes on his feet. His smile radiates friendliness as he gazes directly at the viewer. In his right hand, he holds a small simple black knife that gleams in the sunlight and is placed in front of his body. The scene is realistically full-body portrait image, showing the man standing in a relaxed pose."

2.2 Prompt for depicted target on the lower panel of Figure 1B

"There is an average looking man with Turkish facial features walking on the right site of a street, his whole body is visible. He is wearing a white t-shirt, black pants and white sneakers, and appears to be in motion, and is holding a black smartphone in his left hand, which is placed next to his hip, extended from the body. The man is looking into the camera."

3 AI prompts for Figure 1C

The quality of the generated image for Figure 1C was assessed based on how closely it resembled the original stimuli, specifically regarding the placement and size of the target object in relation to the person's body.

"A sharp full-body image of a Black Man standing in front of a black background. He is wearing a beige sweatshirt, matching sweatpants and black sneakers, 90s style. He is presenting a can of soda in his right hand. The man is standing with his left hand visibly resting on his hip and his right arm resting on his chest. He has a wide stand. He appears to be relaxed and confident."

4 References

1. Correll J, Park B, Judd CM, Wittenbrink B. The police officer's dilemma: Using ethnicity to disambiguate potentially threatening individuals. *Journal of Personality and Social Psychology*. 2002;83(6):1314-29.
2. Essien I, Stelter M, Kalbe F, Koehler A, Mangels J, Meliß S. The shooter bias: Replicating the classic effect and introducing a novel paradigm. *Journal of Experimental Social Psychology*. 2017;70:41-7.
3. Rombach R, Blattmann A, Lorenz D, Esser P, Ommer B, editors. High-resolution image synthesis with latent diffusion models. Proceedings of the IEEE/CVF conference on computer vision and pattern recognition; 2022.
