# Supplementary material for: Effects of mindful breathing meditation on stereotype expression in two randomized controlled double-blinded trials
Source: PLoS One. 2026 Apr 30;21(4):e0347871. doi: 10.1371/journal.pone.0347871 (PMC13132222; doi:10.1371/journal.pone.0347871)
Supplement: S4 Appendix — (PDF) [file pone.0347871.s004.pdf]

S4 Appendix: Model Diagnostics for Drift Diffusion Models

MCMC chain convergence was assessed with the potential scale reduction factor (PSRF). All point estimates of the PSRF for condition and individual level parameters = 1, the multivariate PSRF for each model was < 1.2, indicating acceptable convergence for all parameters [1]. MCMC autocorrelation was assessed by effective sample sizes (ESSs). Most ESS values were ≥ 10.000, indicating accurate and stable estimates of the posterior distribution [2], but a few ESS were < 10.000. Since we extracted posterior means for further frequentist analyses and ESS ≥ 10.000 are especially recommended when working with 95% highest density intervals [3], this was not a concern. Relatedly, Monte Carlo standard errors (MCSE) were ≤ 0.01, indicating stable posterior mean estimations for all parameters, which is considered to be especially important for downstream frequentist analyses with posterior means [3, 4].

Condition level parameters will be presented for all models estimated. See Table 1 for a legend of parameter names and trial conditions.

Table 1
Parameter Estimates and Respective Trial Conditions

Parameter		Shooter Task	Avoidance Task
muAlpha[1]	muBeta[1]	White targets	German targets
muAlpha[2]	muBeta[2]	Black targets	Turkish targets
muDelta[1]	muTau[1]	White unarmed targets	German unarmed targets
muDelta[2]	muTau[2]	Black unarmed targets	Turkish unarmed targets
muDelta[3]	muTau[3]	White armed targets	German armed targets
muDelta[4]	muTau[4]	Black armed targets	Turkish armed targets

Figure 1

Experiment 1: Model Diagnostics for the Drift Diffusion Model for the Mindfulness Condition at Pre-Measurement (Shooter Task)

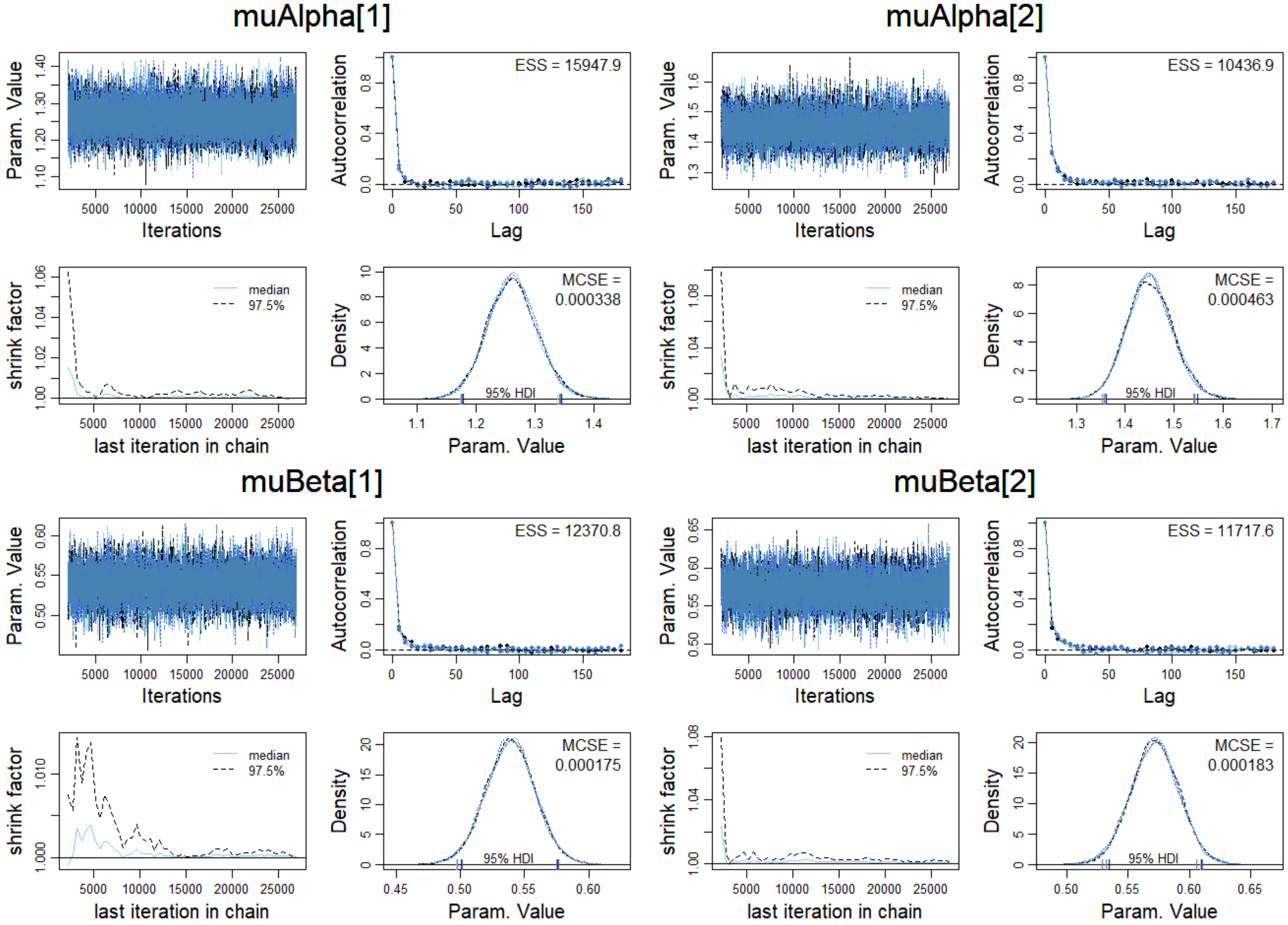

Figure 1
(continued)

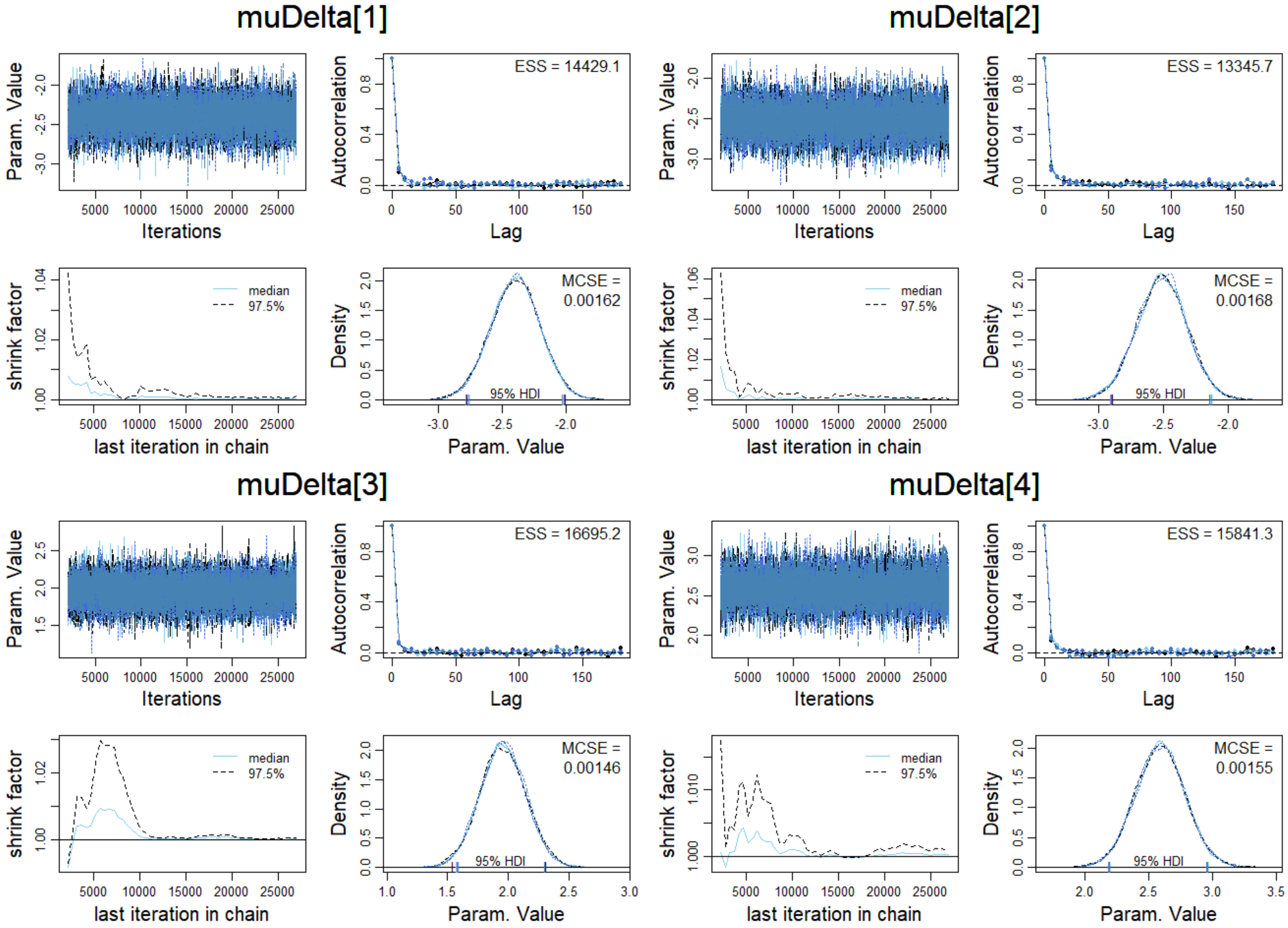

Figure 1
(continued)

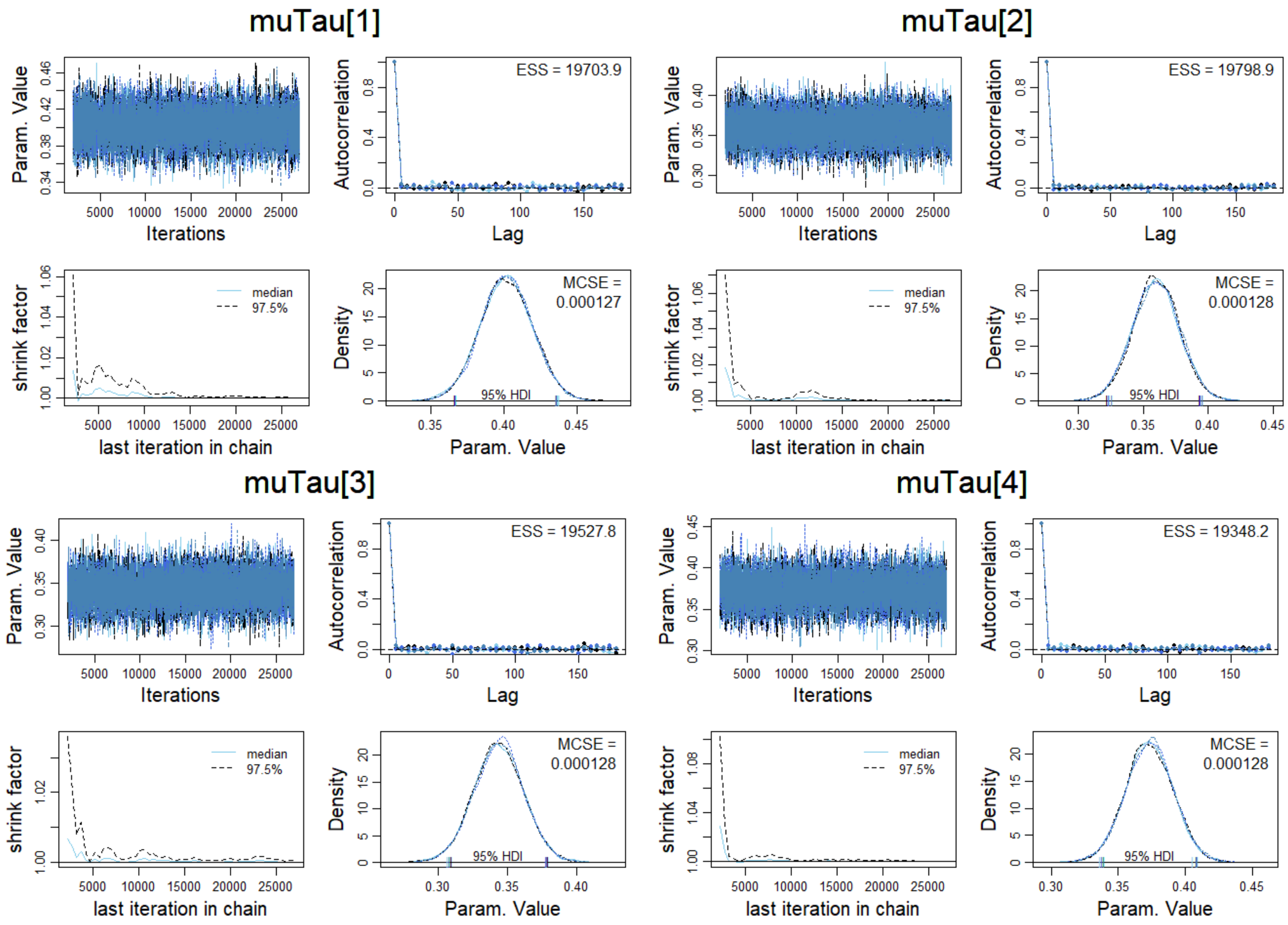

Figure 2

Experiment 1: Model Diagnostics for the Drift Diffusion Model for the Mindfulness Condition at Post-Measurement (Shooter Task)

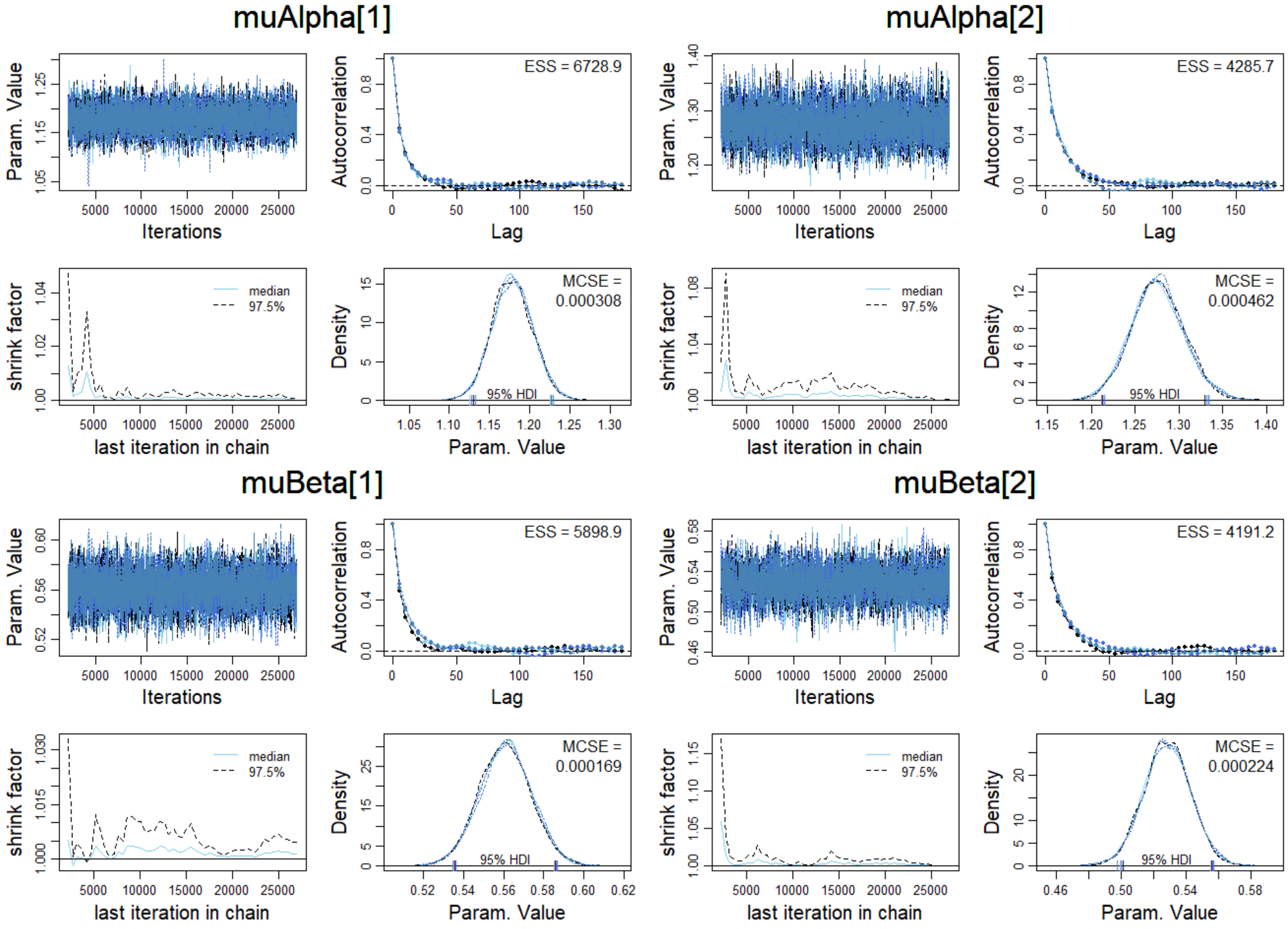

Figure 2
(continued)

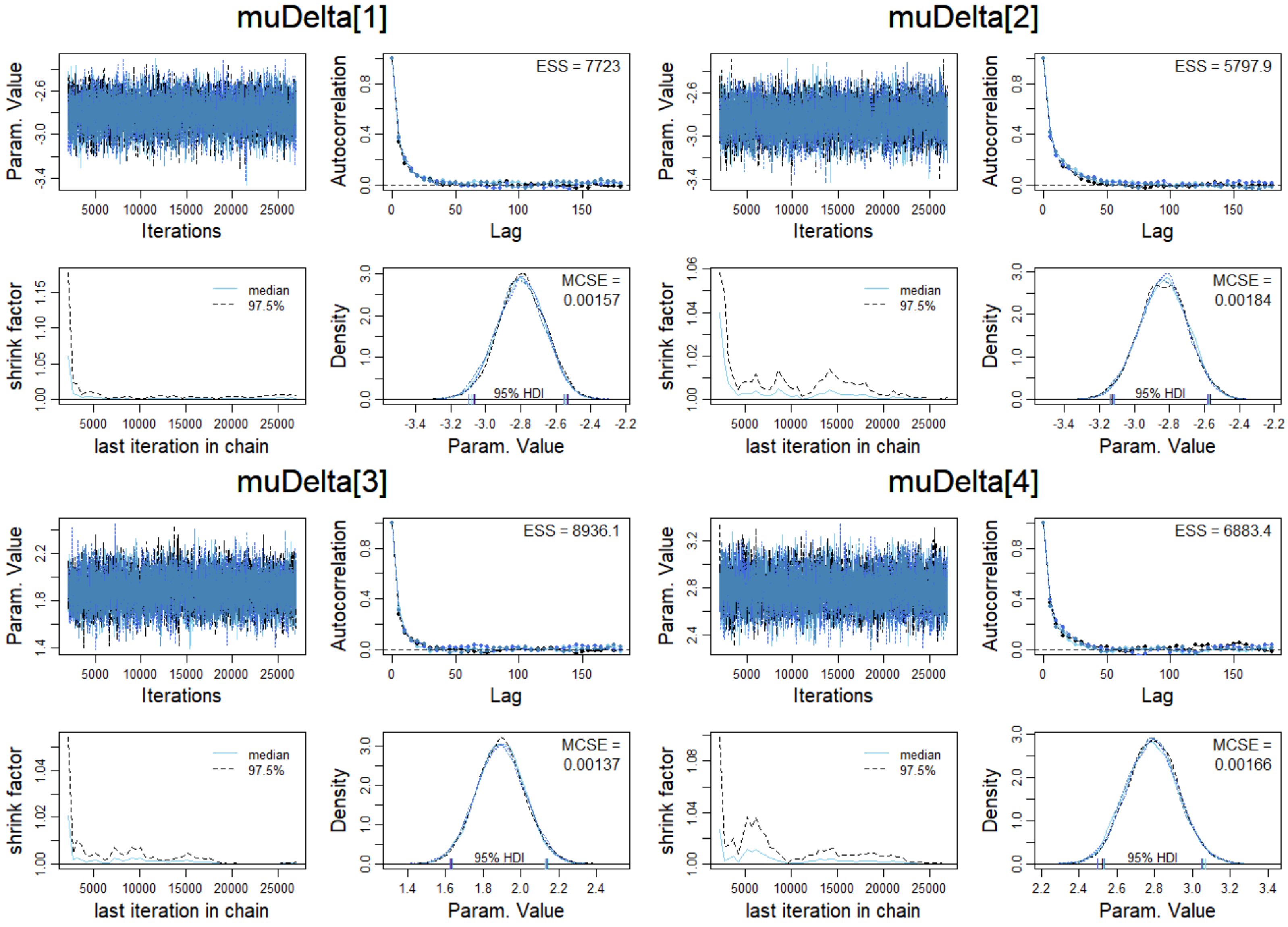

Figure 2
(continued)

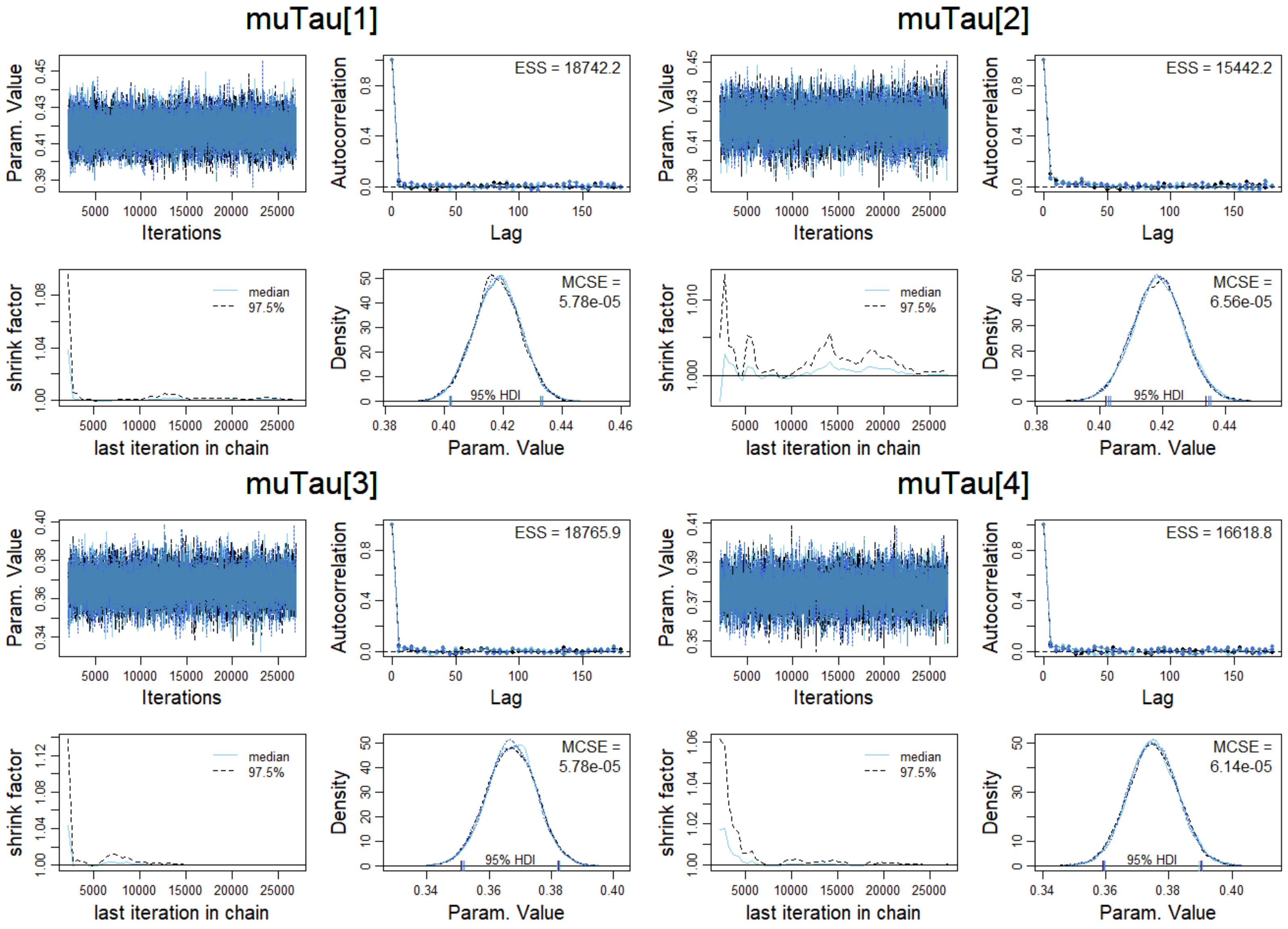

Figure 3

Experiment 1: Model Diagnostics for the Drift Diffusion Model for the PMR Condition at Pre-Measurement (Shooter Task)

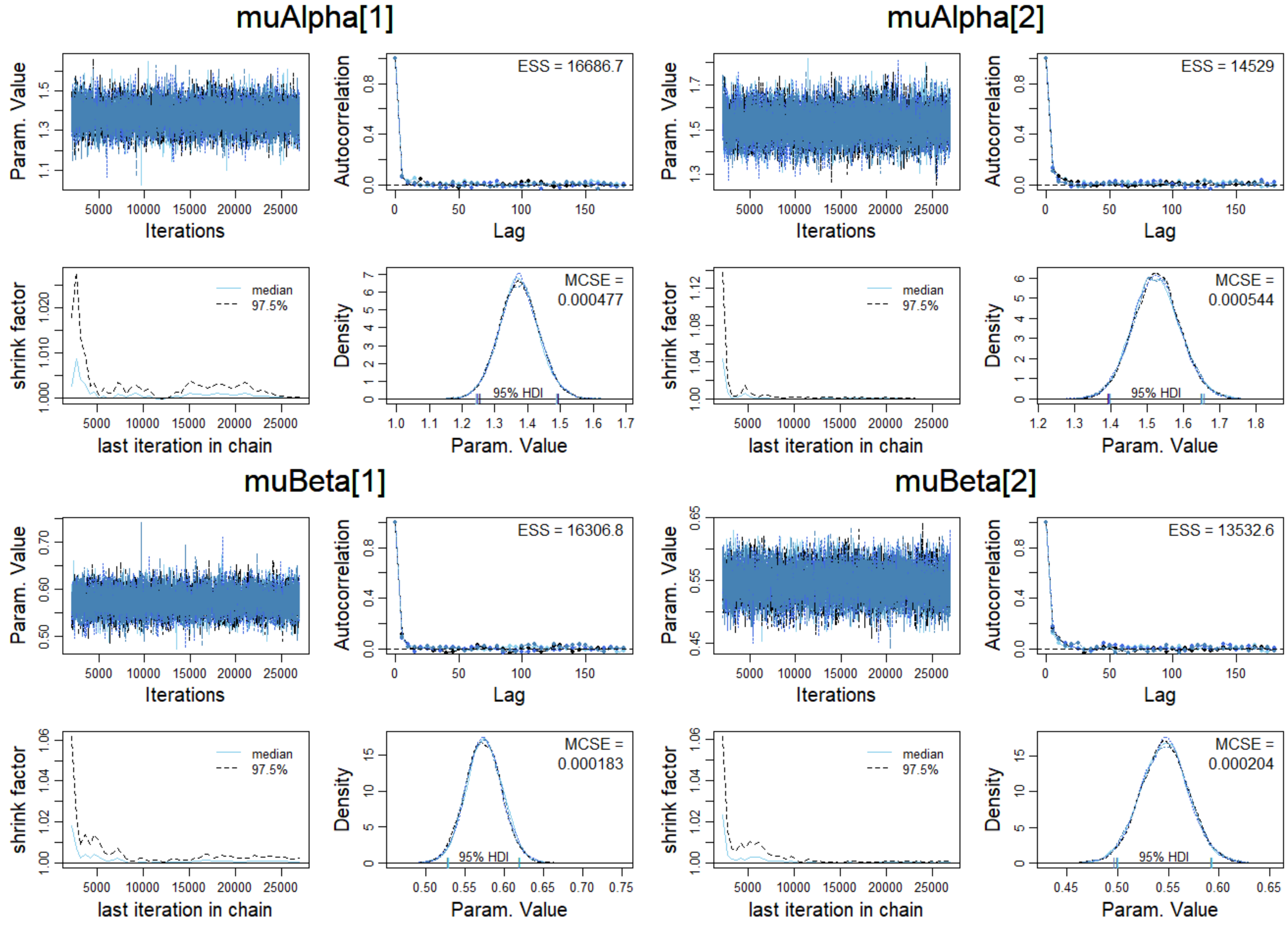

Figure 3

(continued)

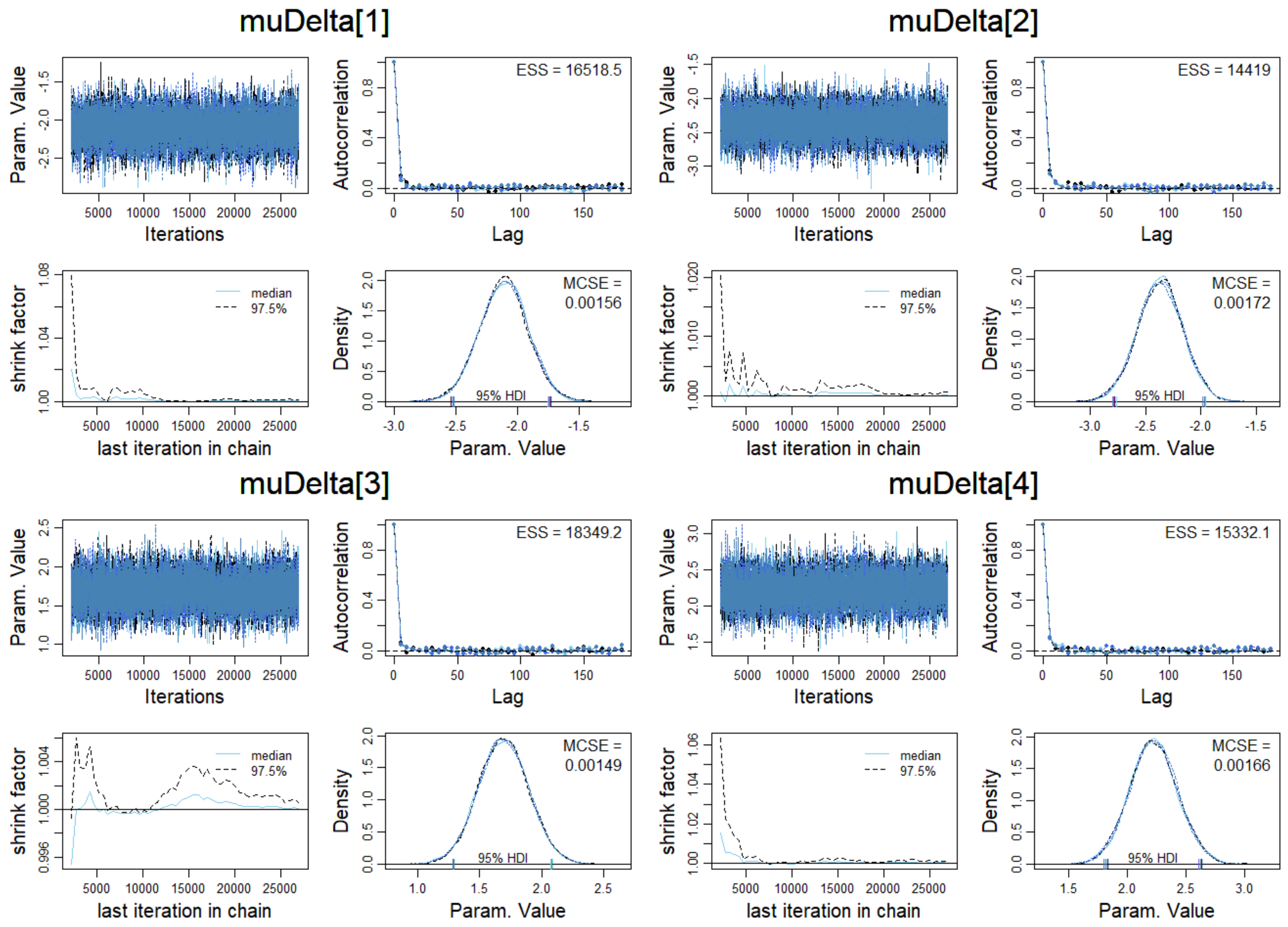

Figure 3
(continued)

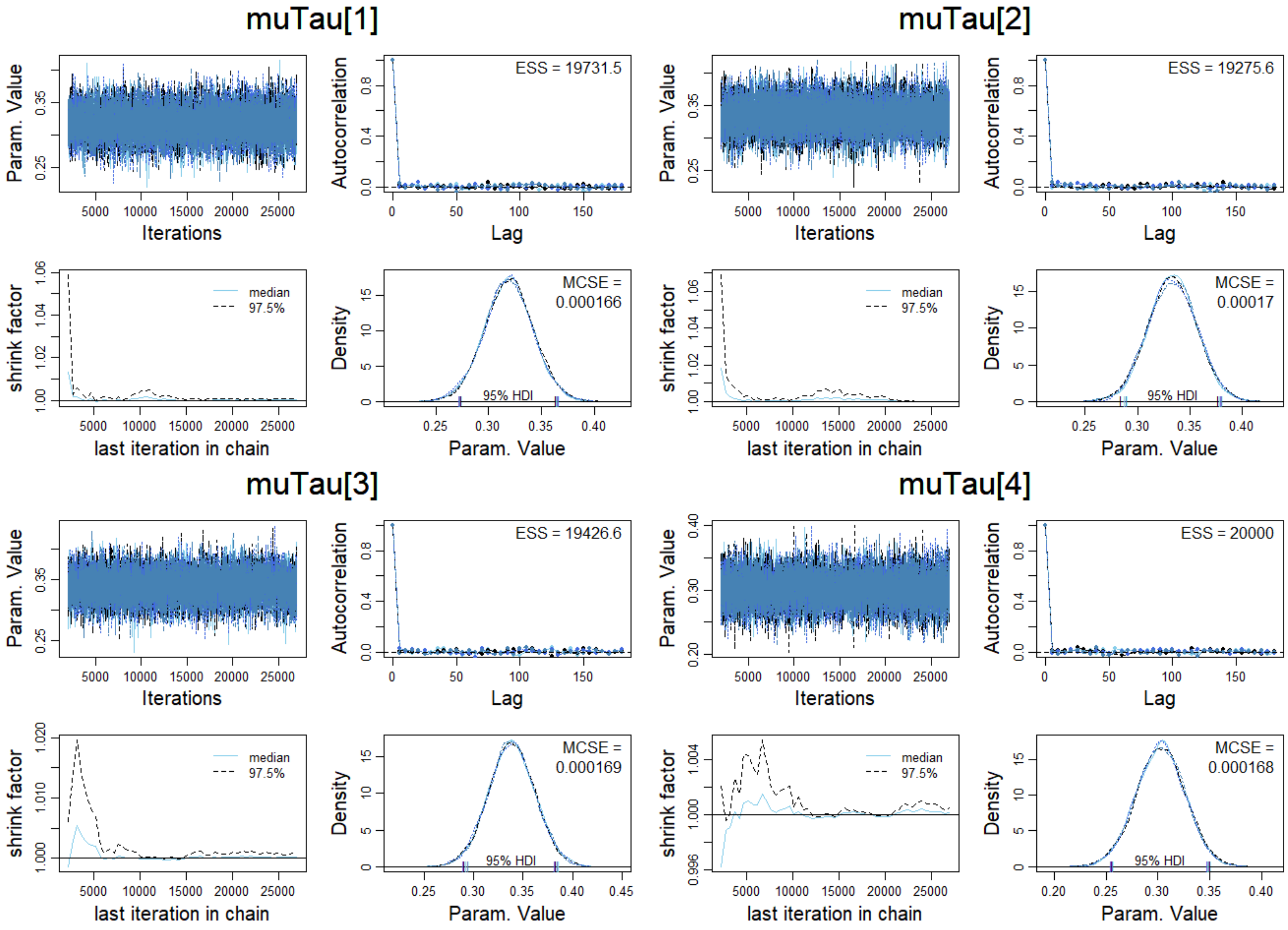

Figure 4

Experiment 1: Model Diagnostics for the Drift Diffusion Model for the PMR Condition at Post-Measurement (Shooter Task)

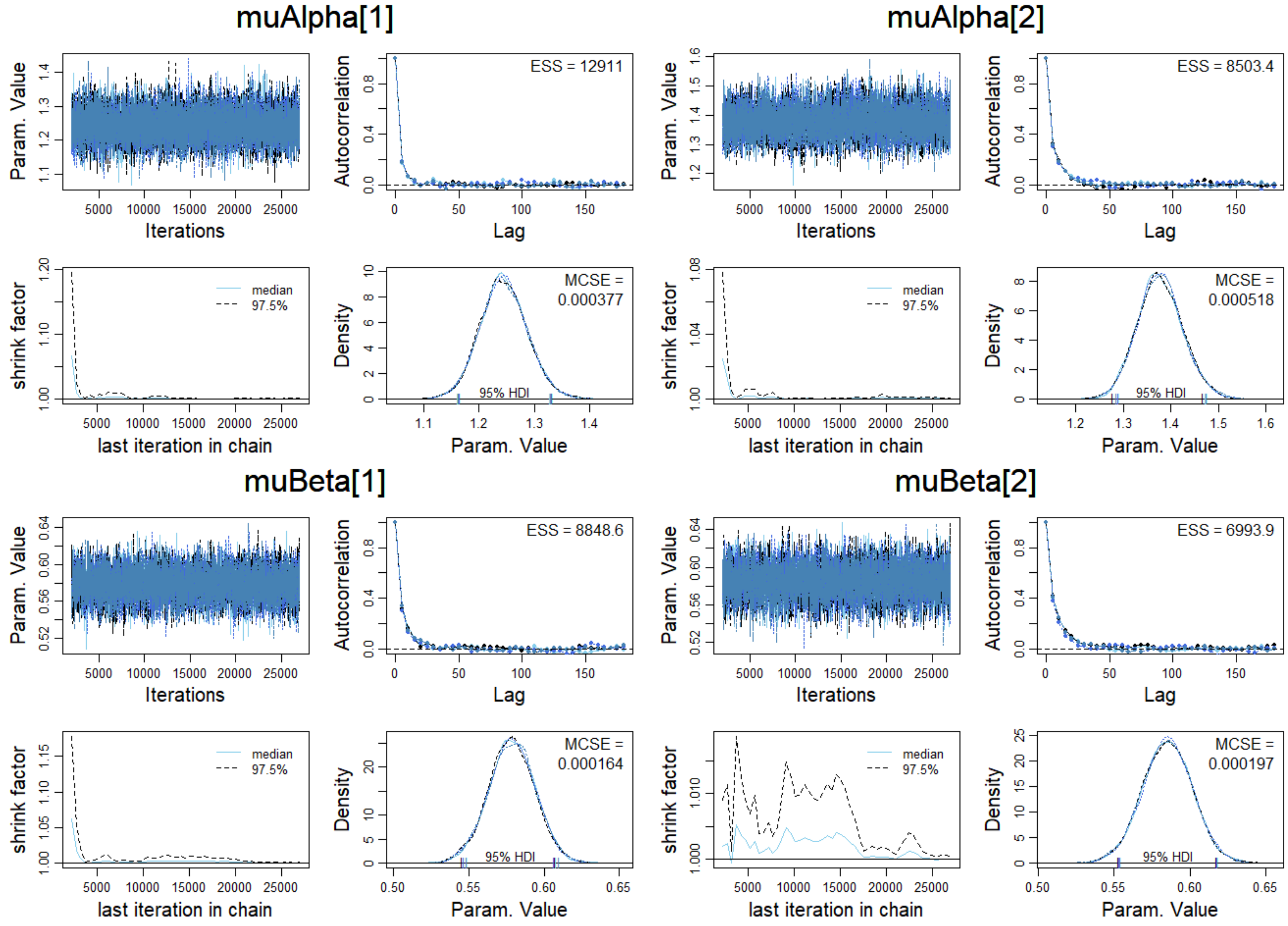

Figure 4
(continued)

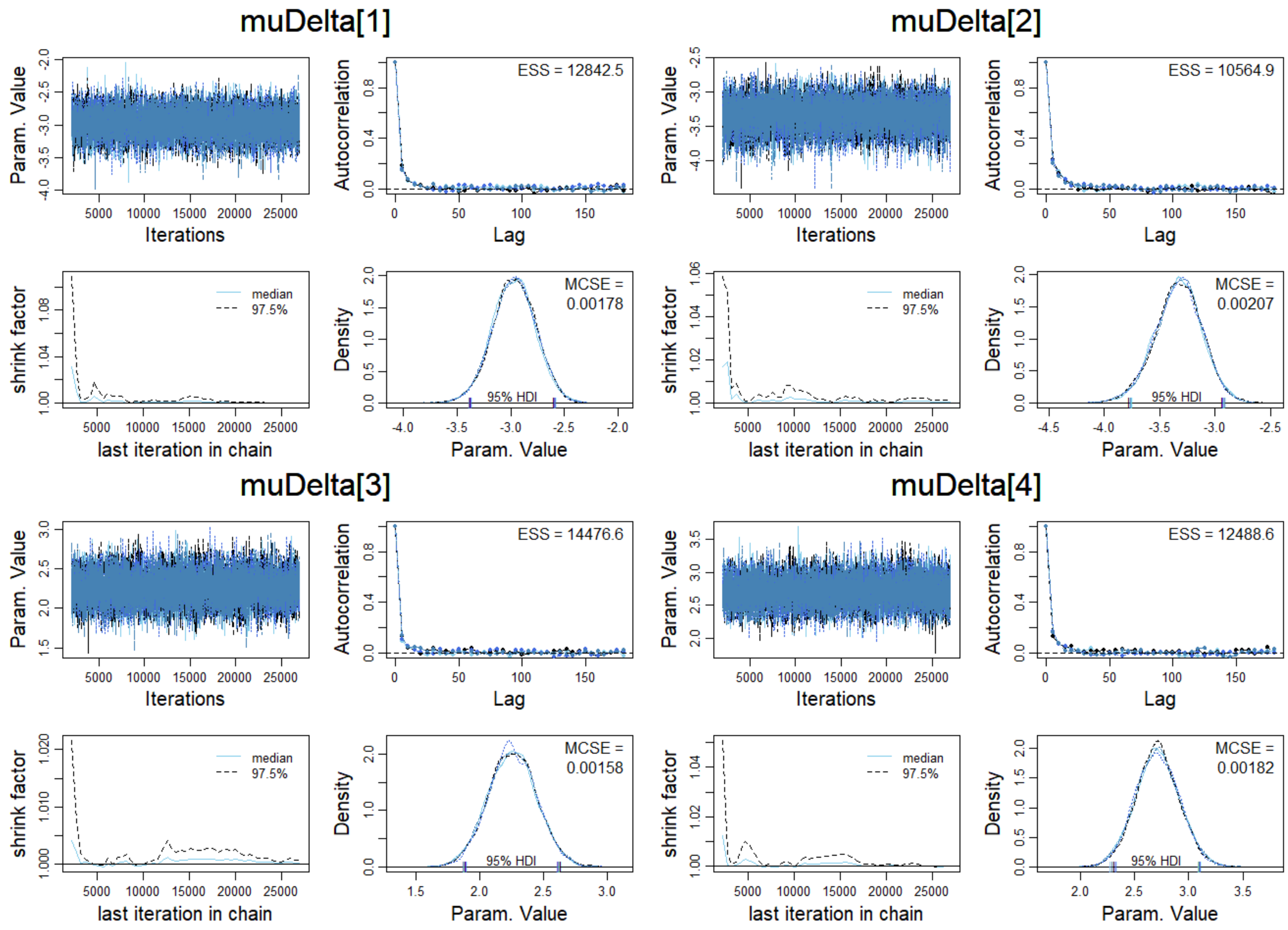

Figure 4
(continued)

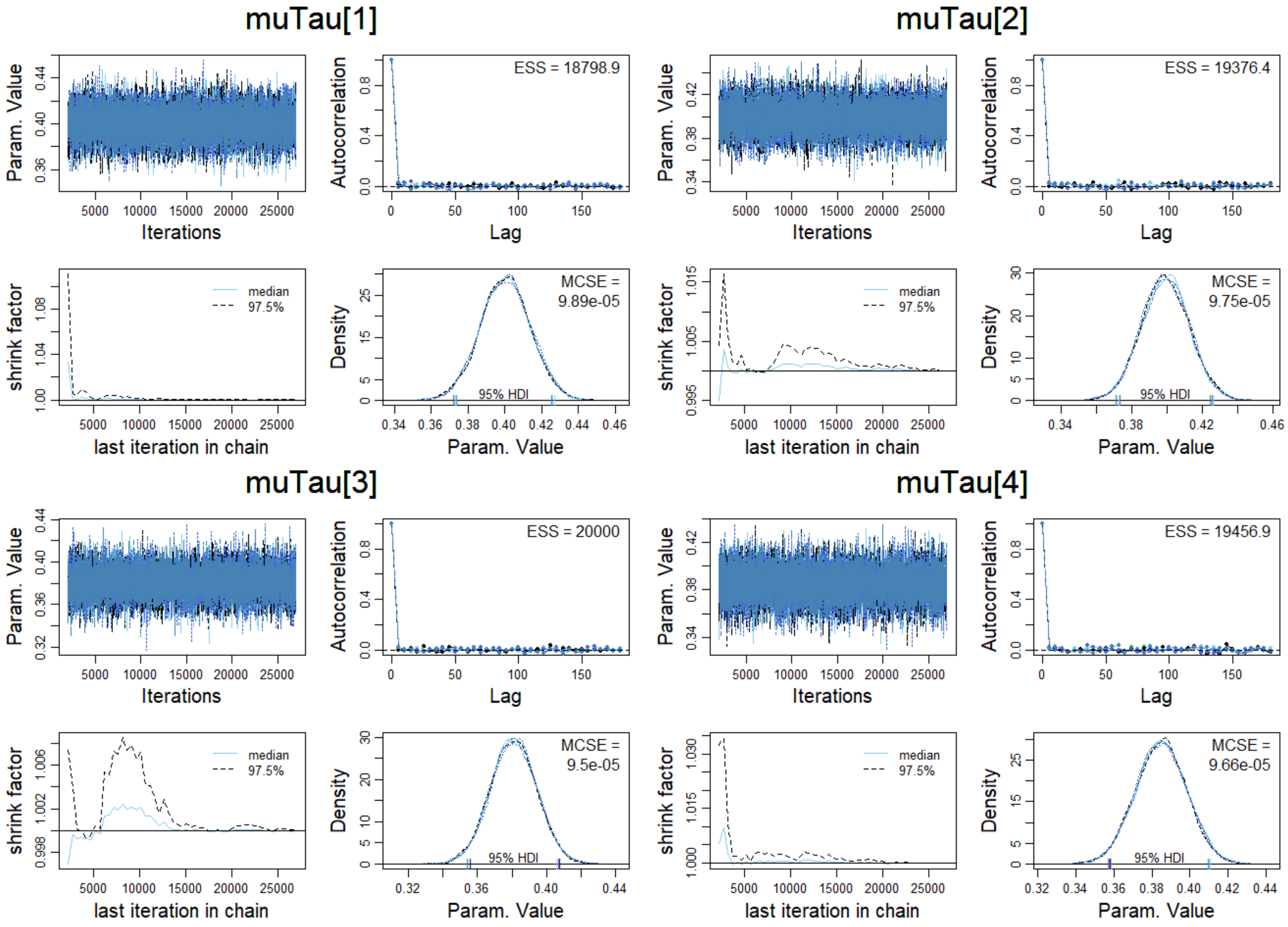

Figure 5

Experiment 1: Model Diagnostics for the Drift Diffusion Model for the Podcast Listening Condition at Pre-Measurement (Shooter Task)

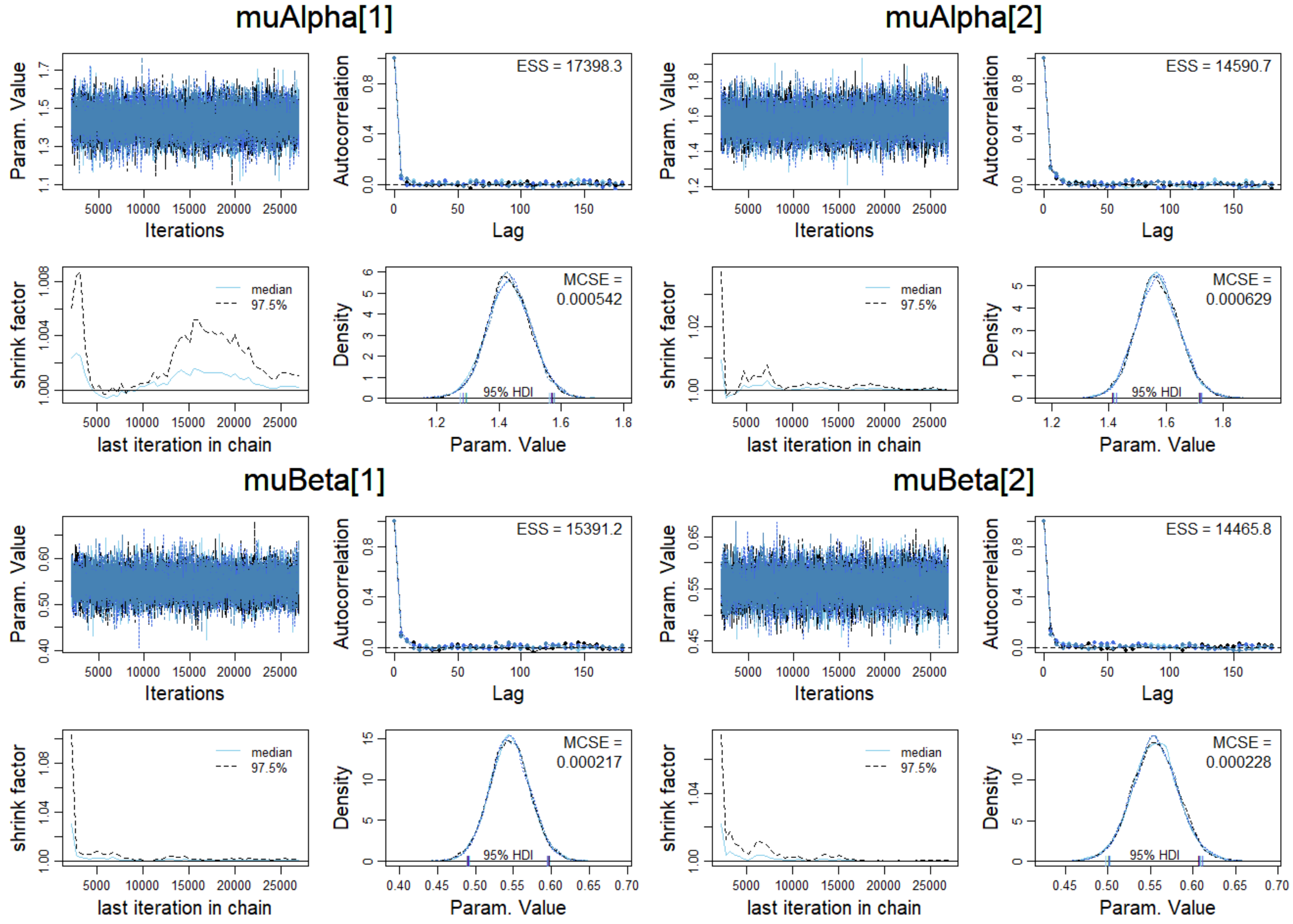

Figure 5
(continued)

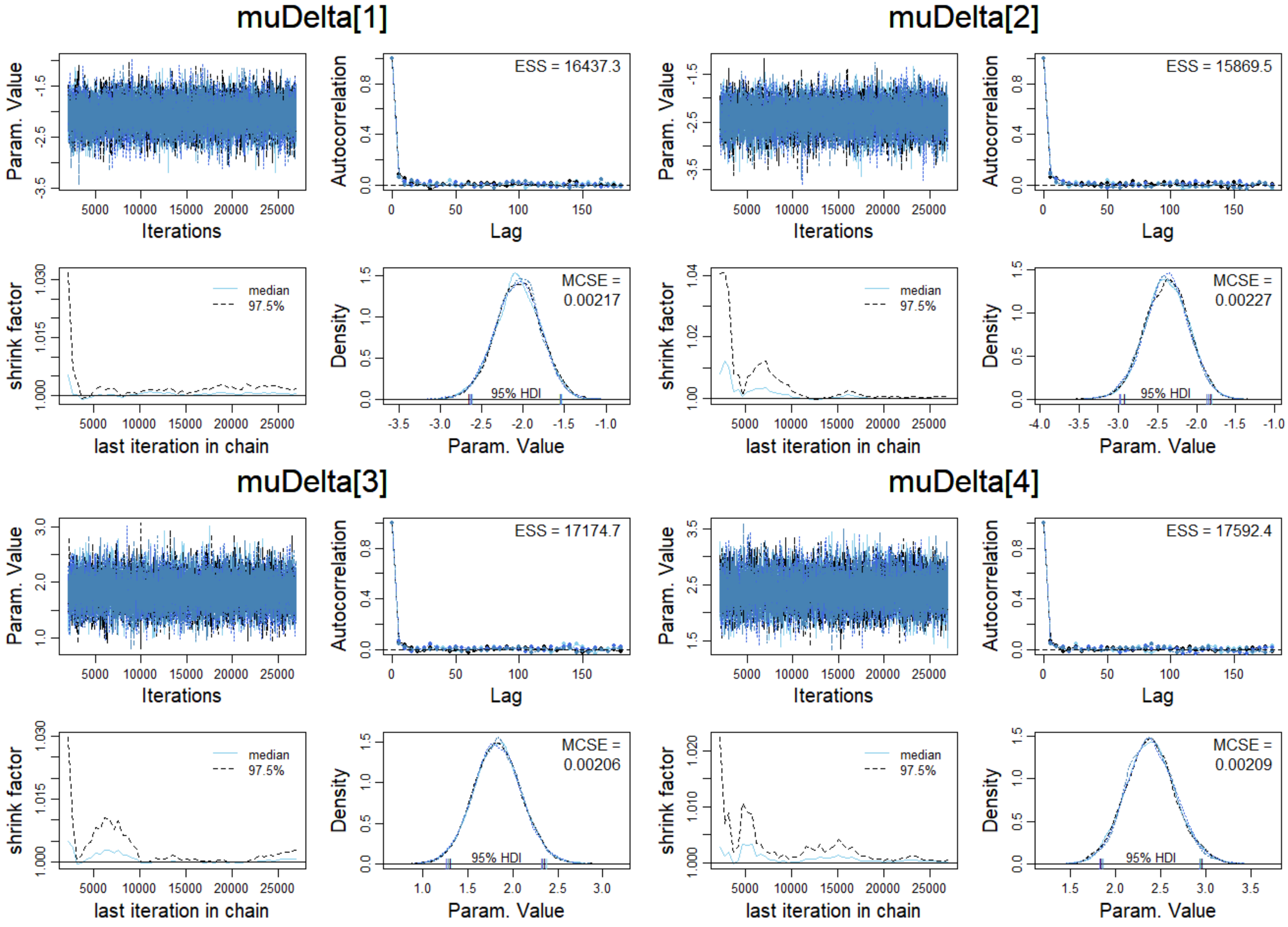

Figure 5
(continued)

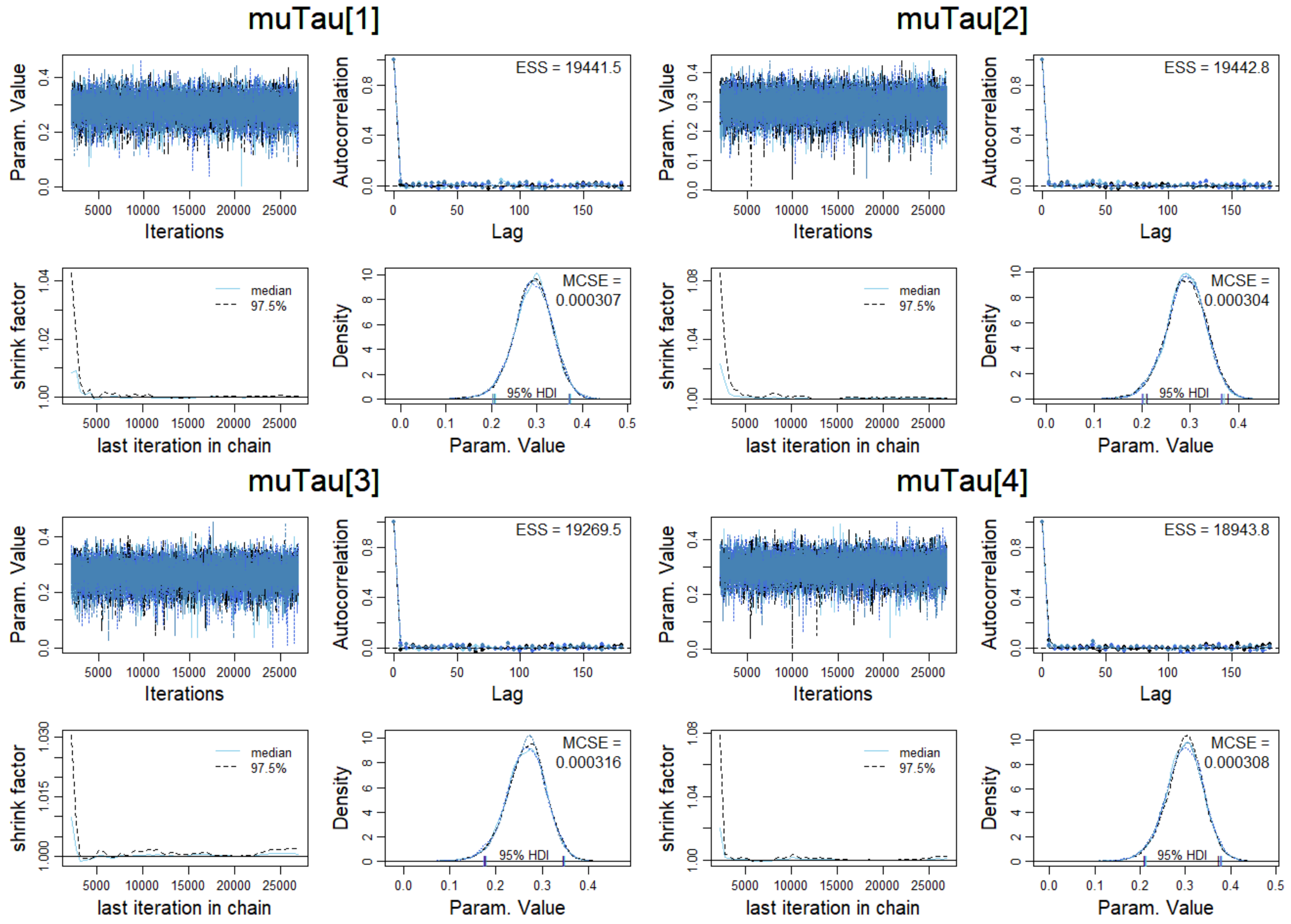

Figure 6

Experiment 1: Model Diagnostics for the Drift Diffusion Model for the Podcast Listening Condition at Post-Measurement (Shooter Task)

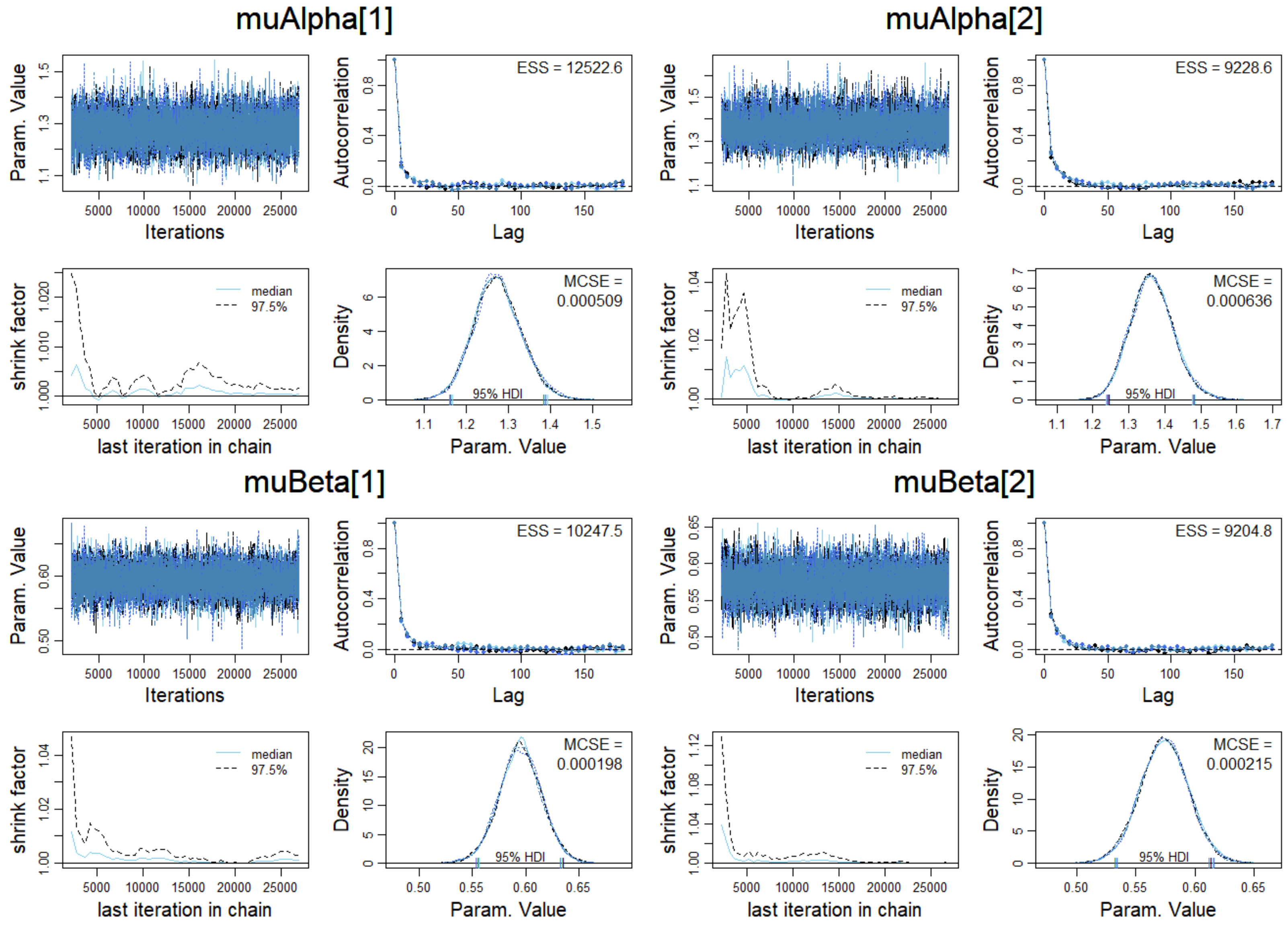

Figure 6

(continued)

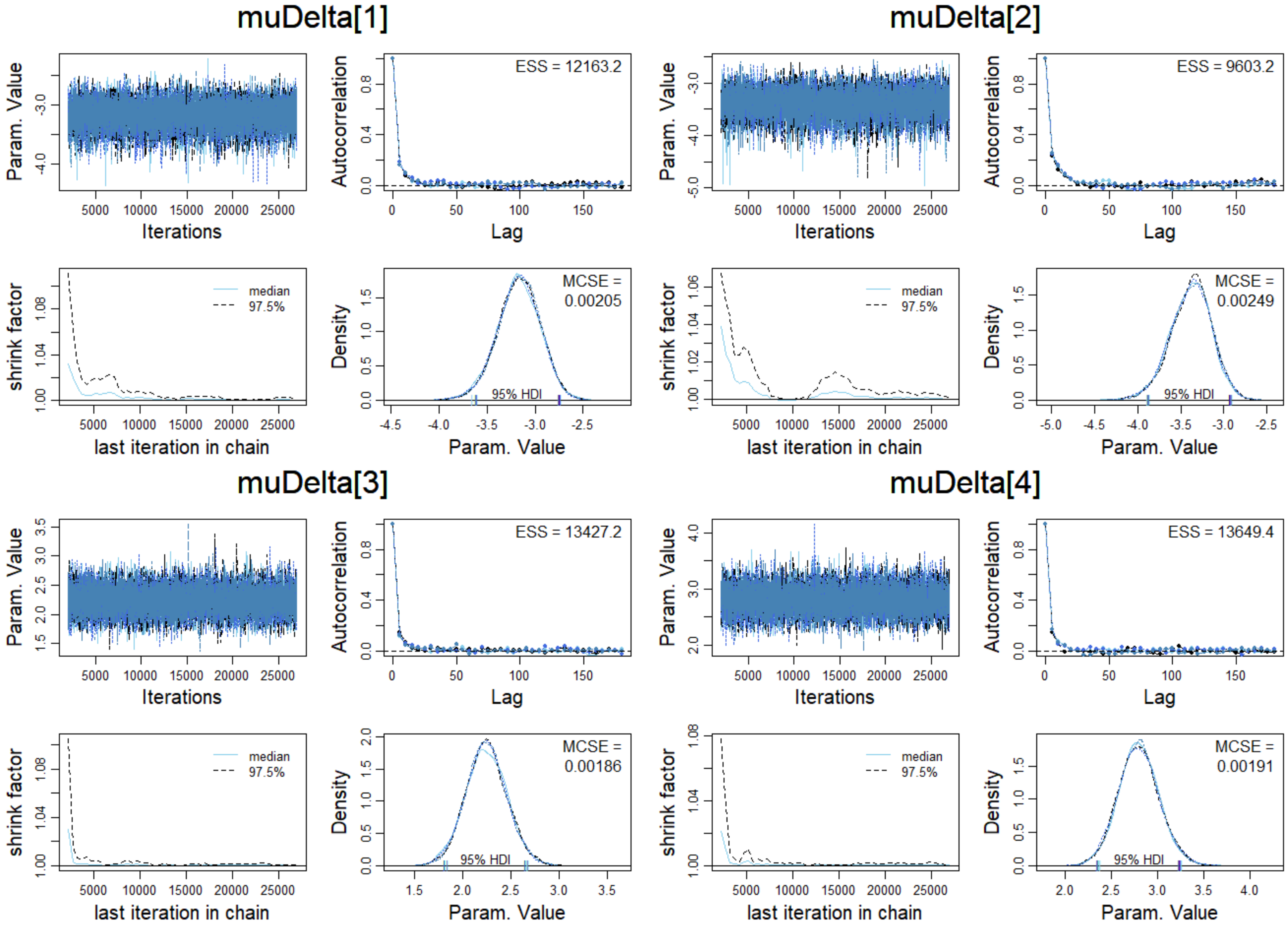

Figure 6
(continued)

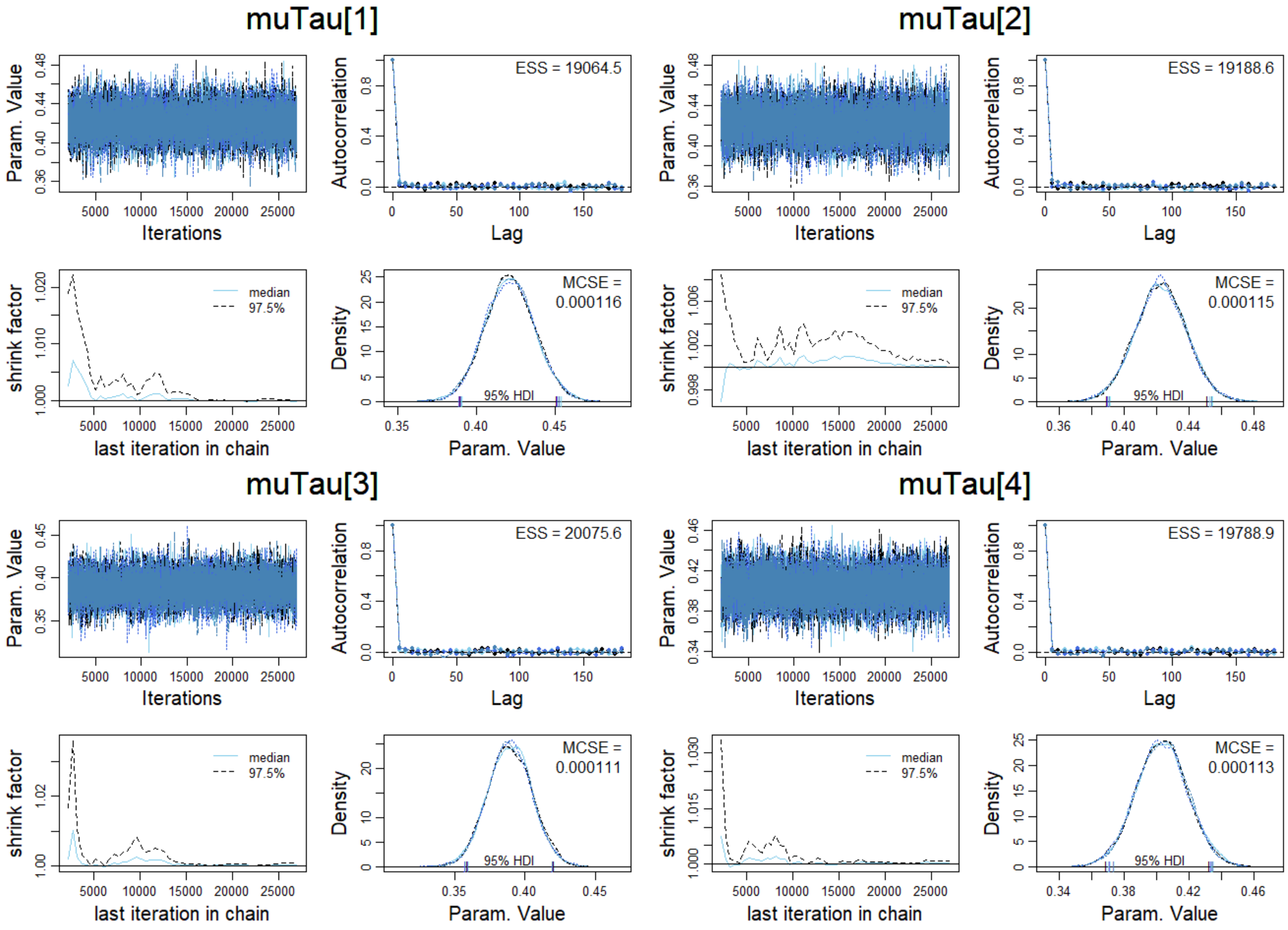

Figure 7

Experiment 2: Model Diagnostics for the Drift Diffusion Model for the Mindfulness Condition at Pre-Measurement (Avoidance Task)

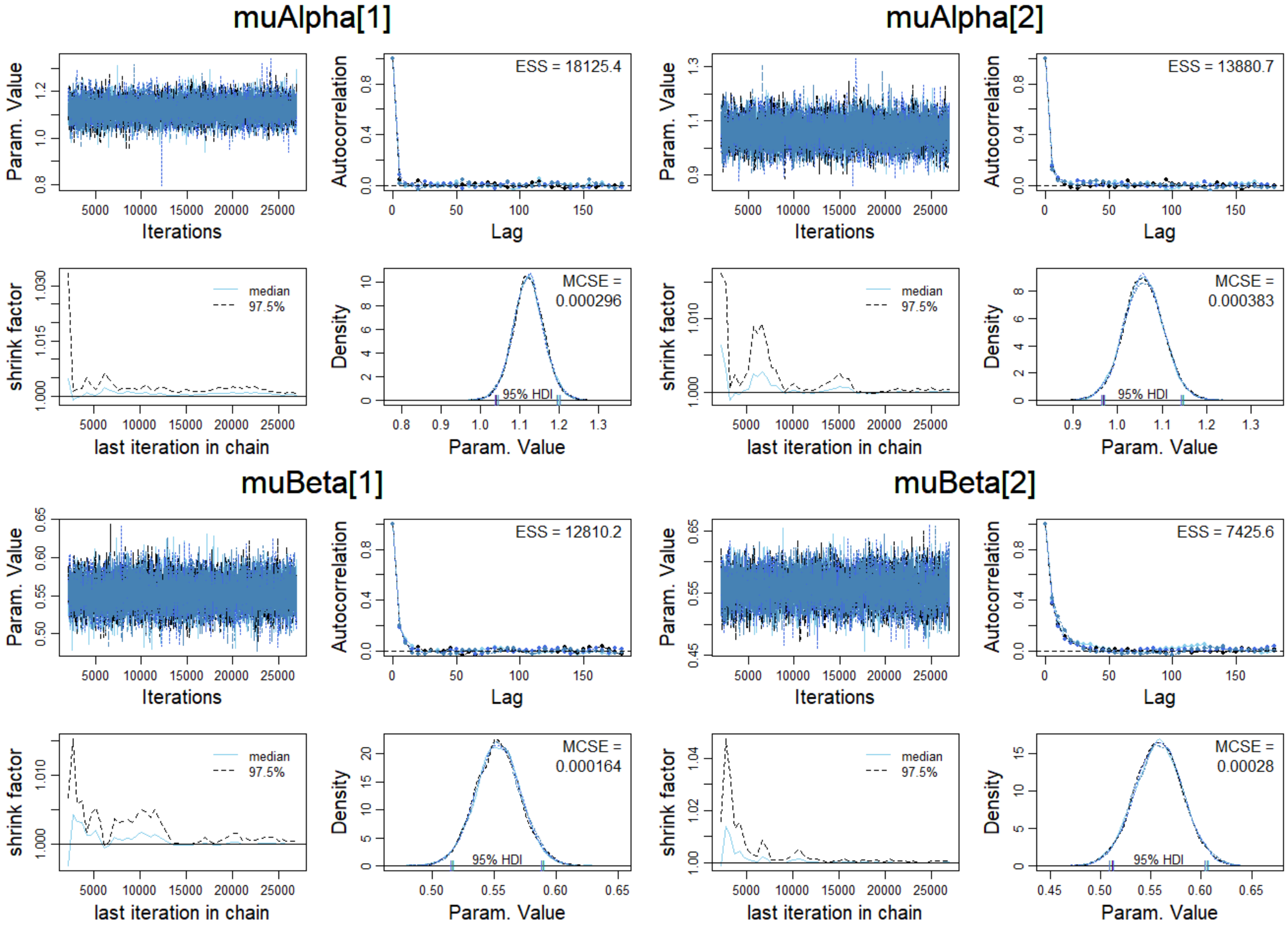

Figure 7
(continued)

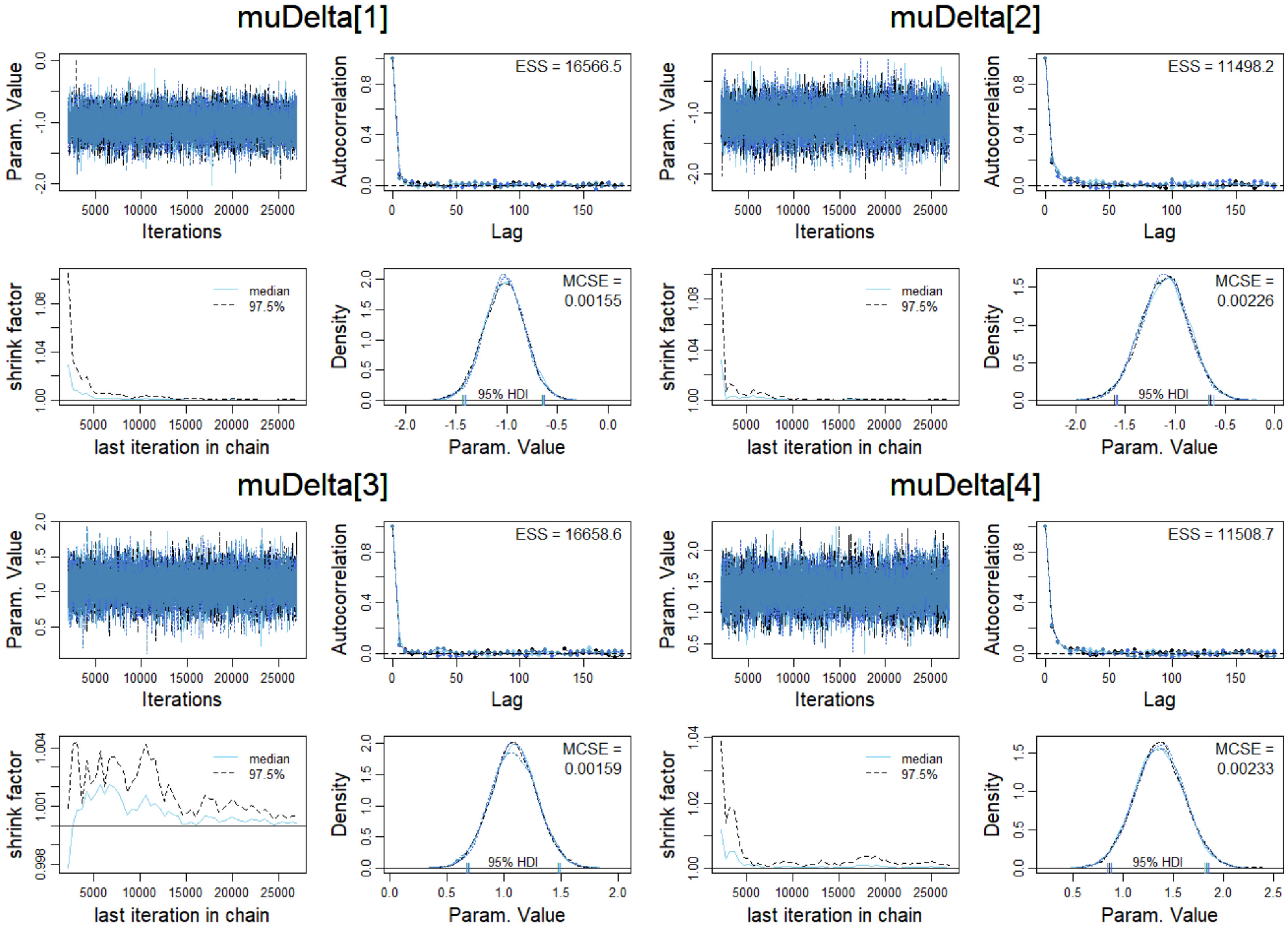

Figure 7
(continued)

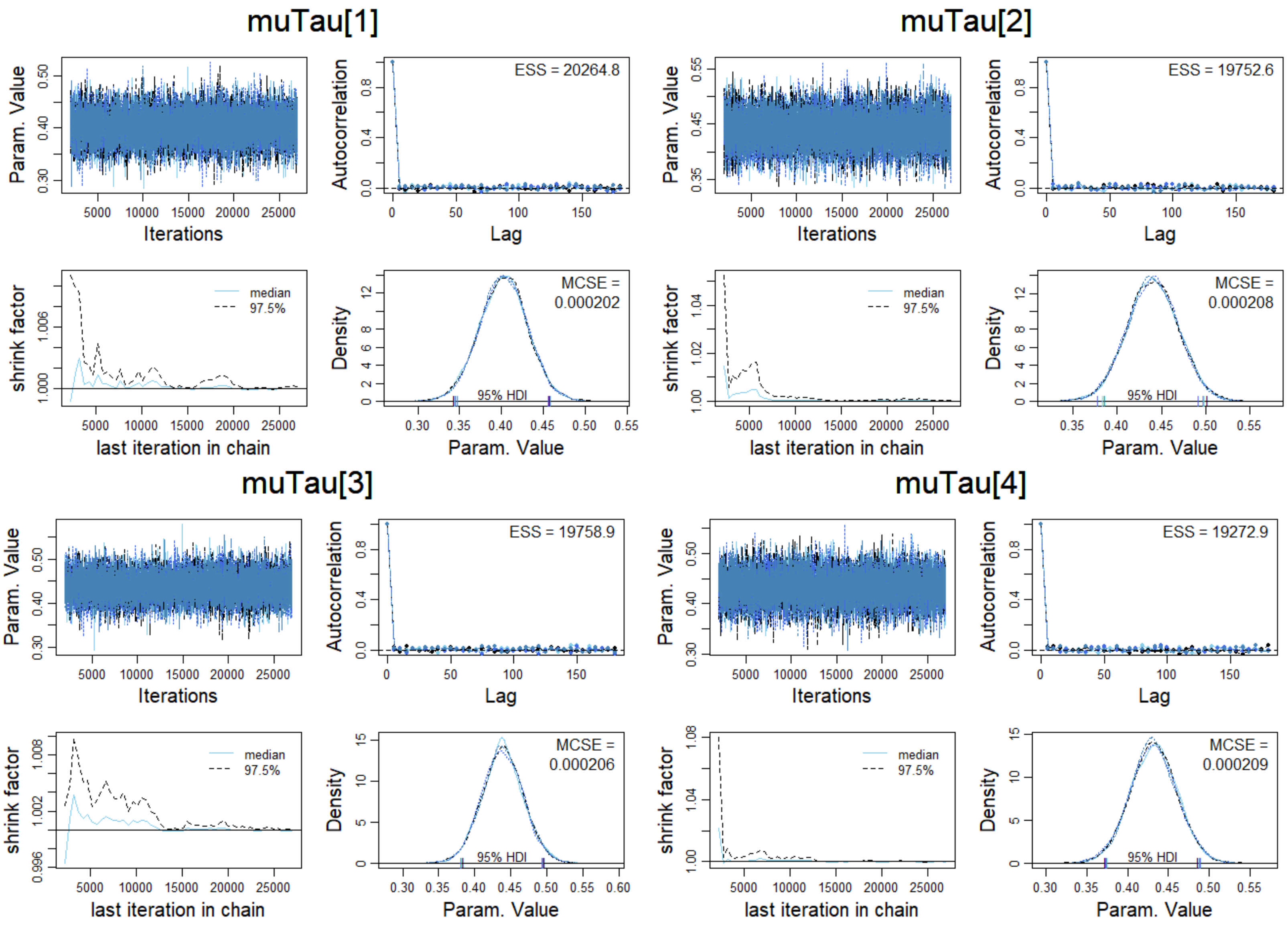

Figure 8

Experiment 2: Model Diagnostics for the Drift Diffusion Model for the Mindfulness Condition at Post-Measurement (Avoidance Task)

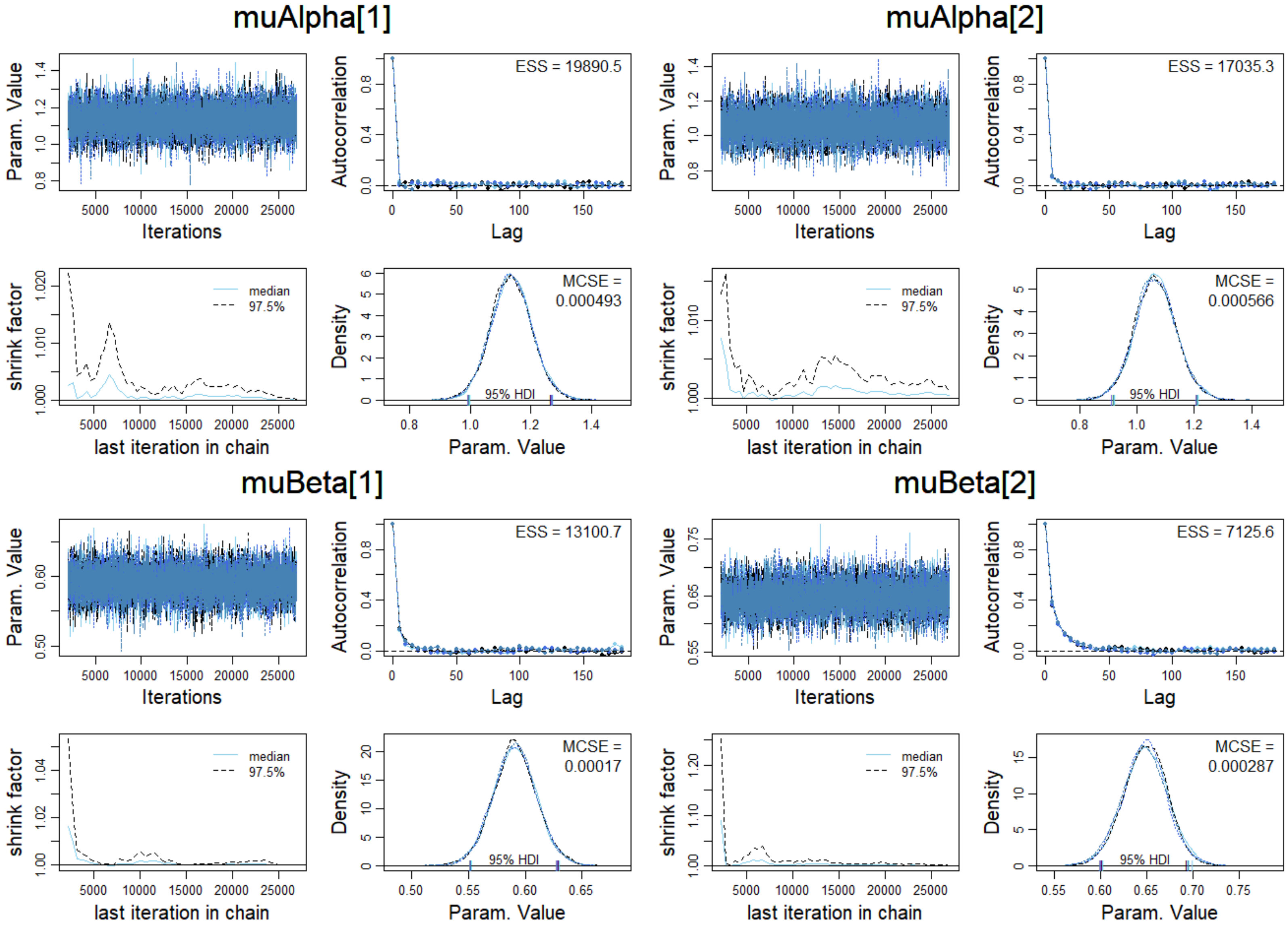

Figure 8
(continued)

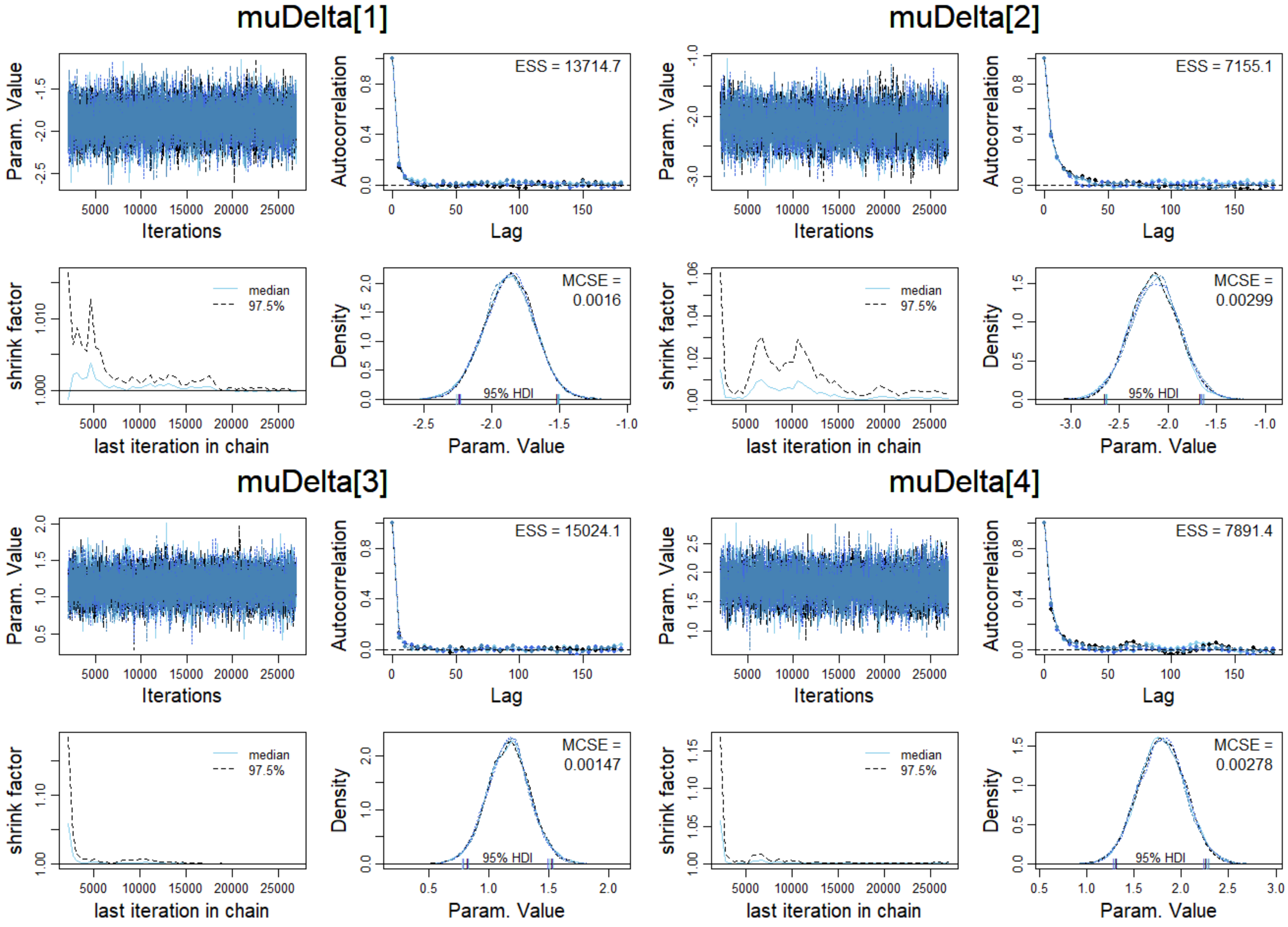

Figure 8
(continued)

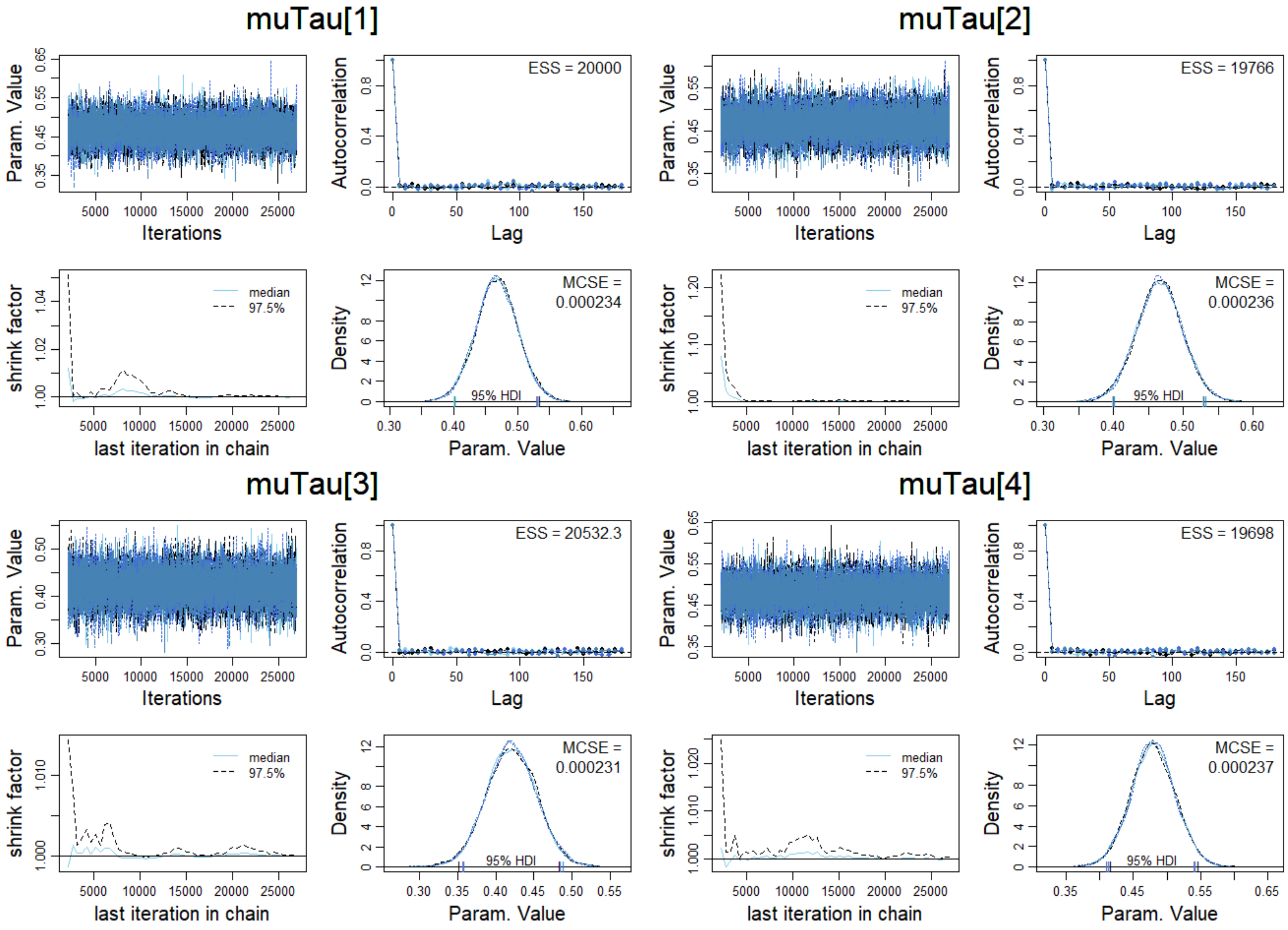

Figure 9

Experiment 2: Model Diagnostics for the Drift Diffusion Model for the PMR Condition at Pre-Measurement (Avoidance Task)

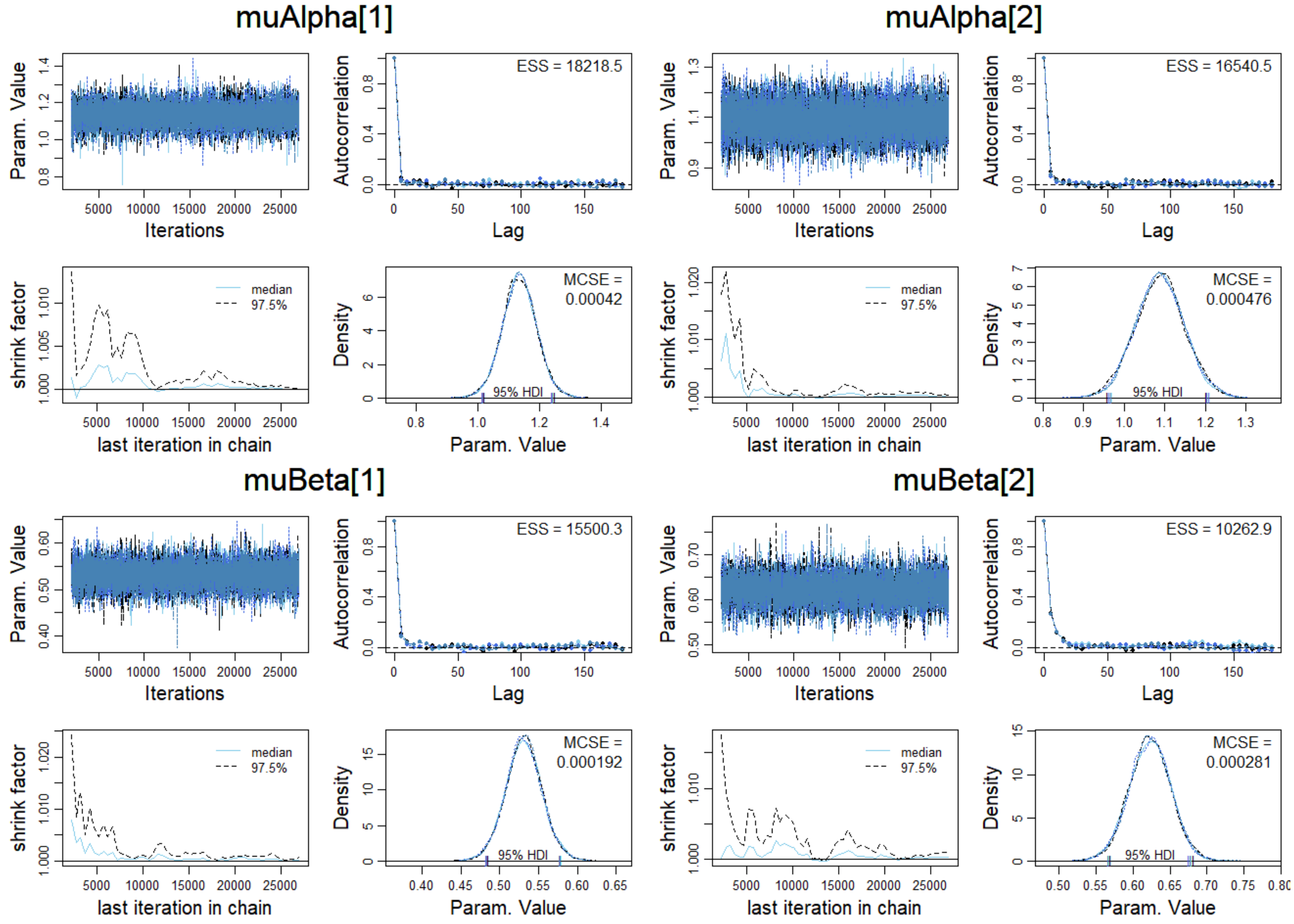

Figure 9
(continued)

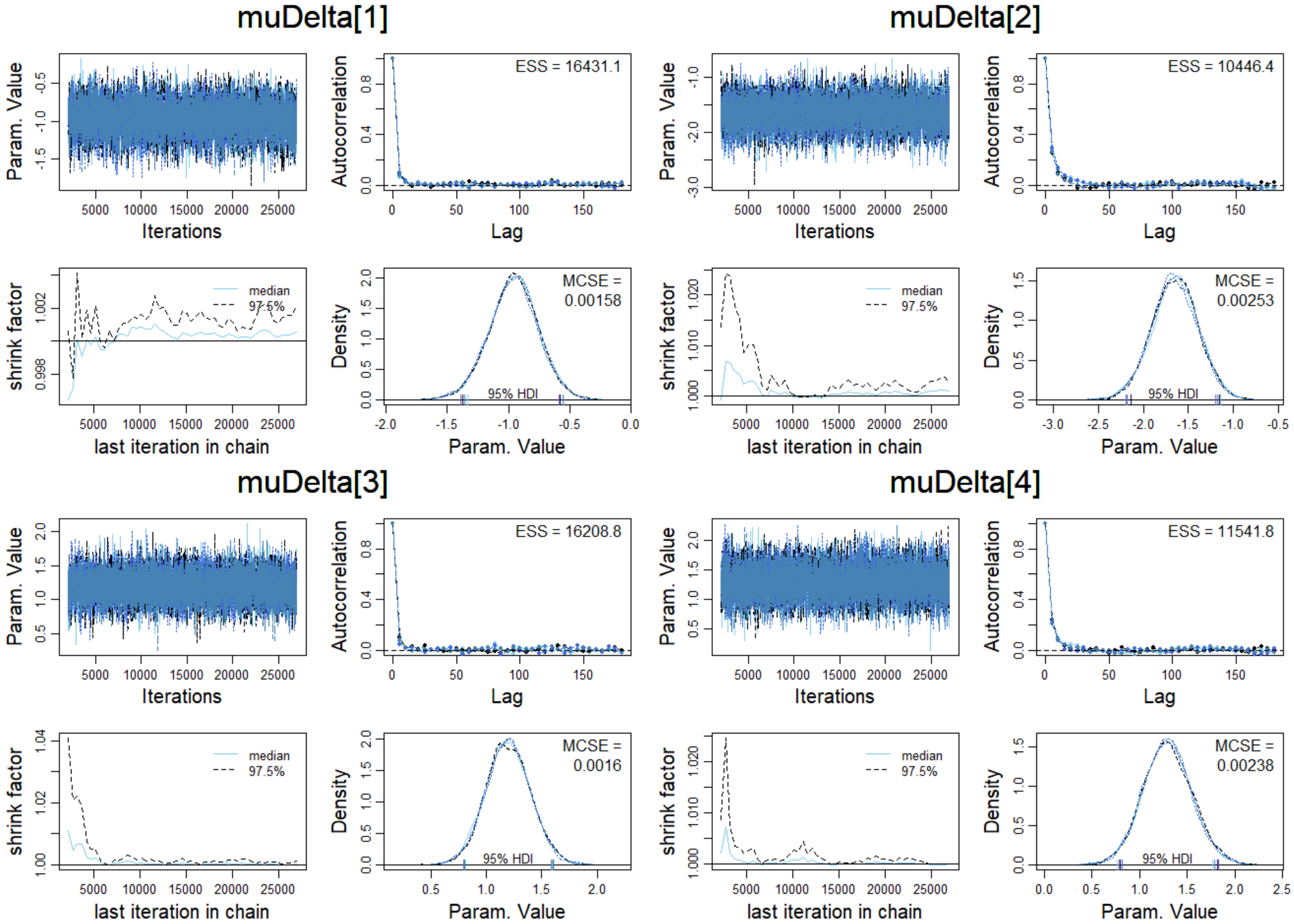

Figure 9
(continued)

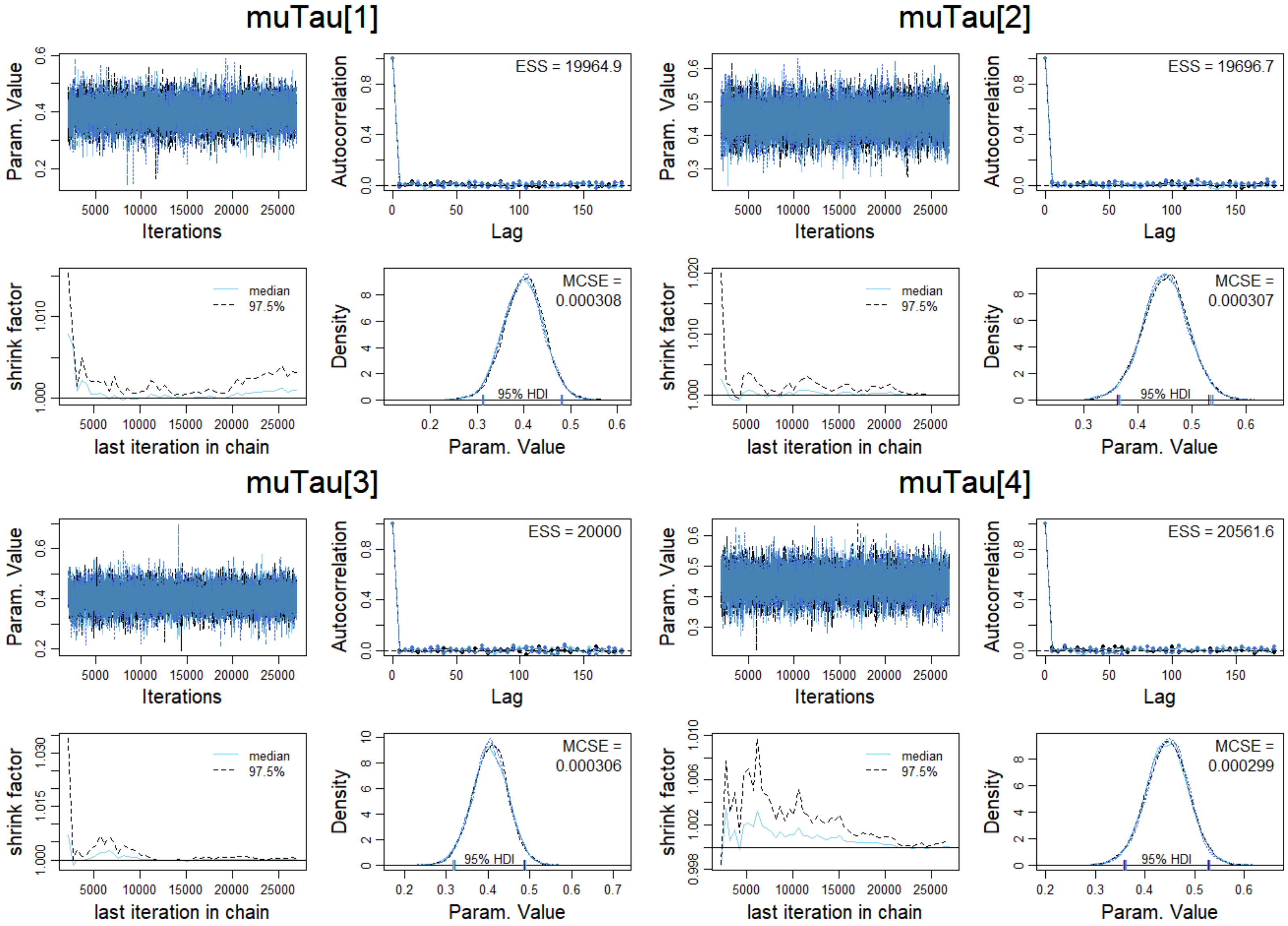

Figure 10

Experiment 2: Model Diagnostics for the Drift Diffusion Model for the PMR Condition at Post-Measurement (Avoidance Task)

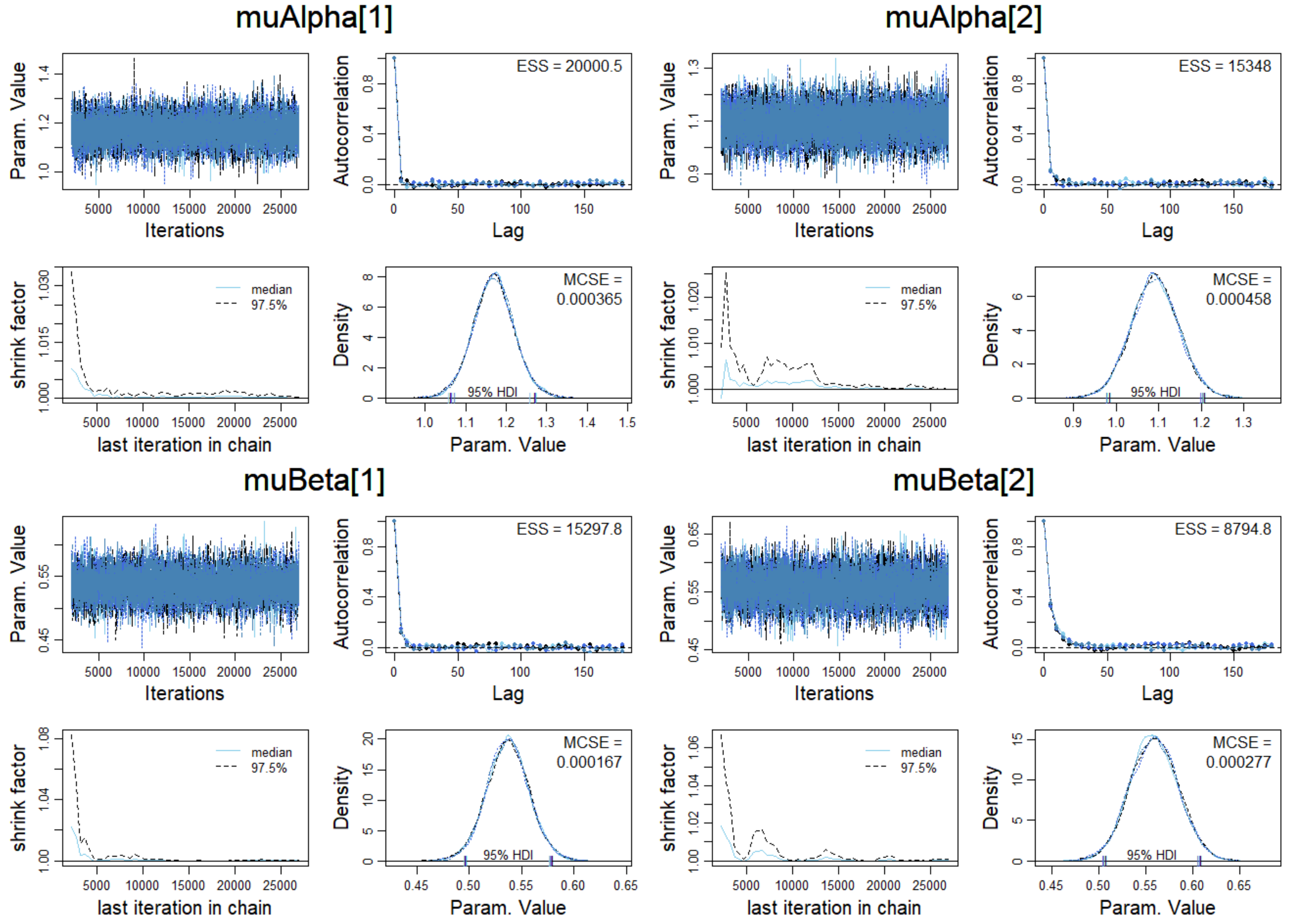

Figure 10
(continued)

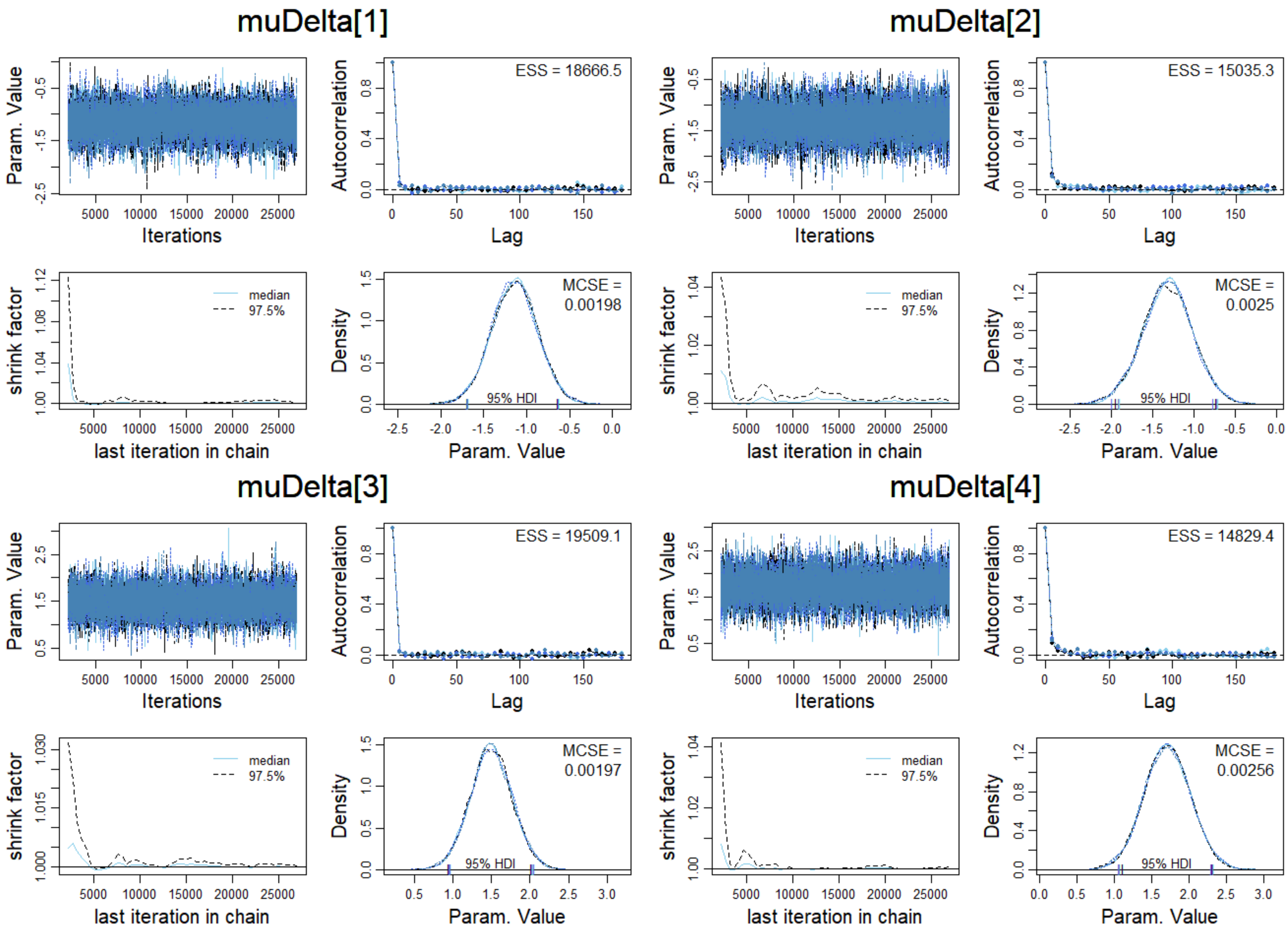

Figure 10
(continued)

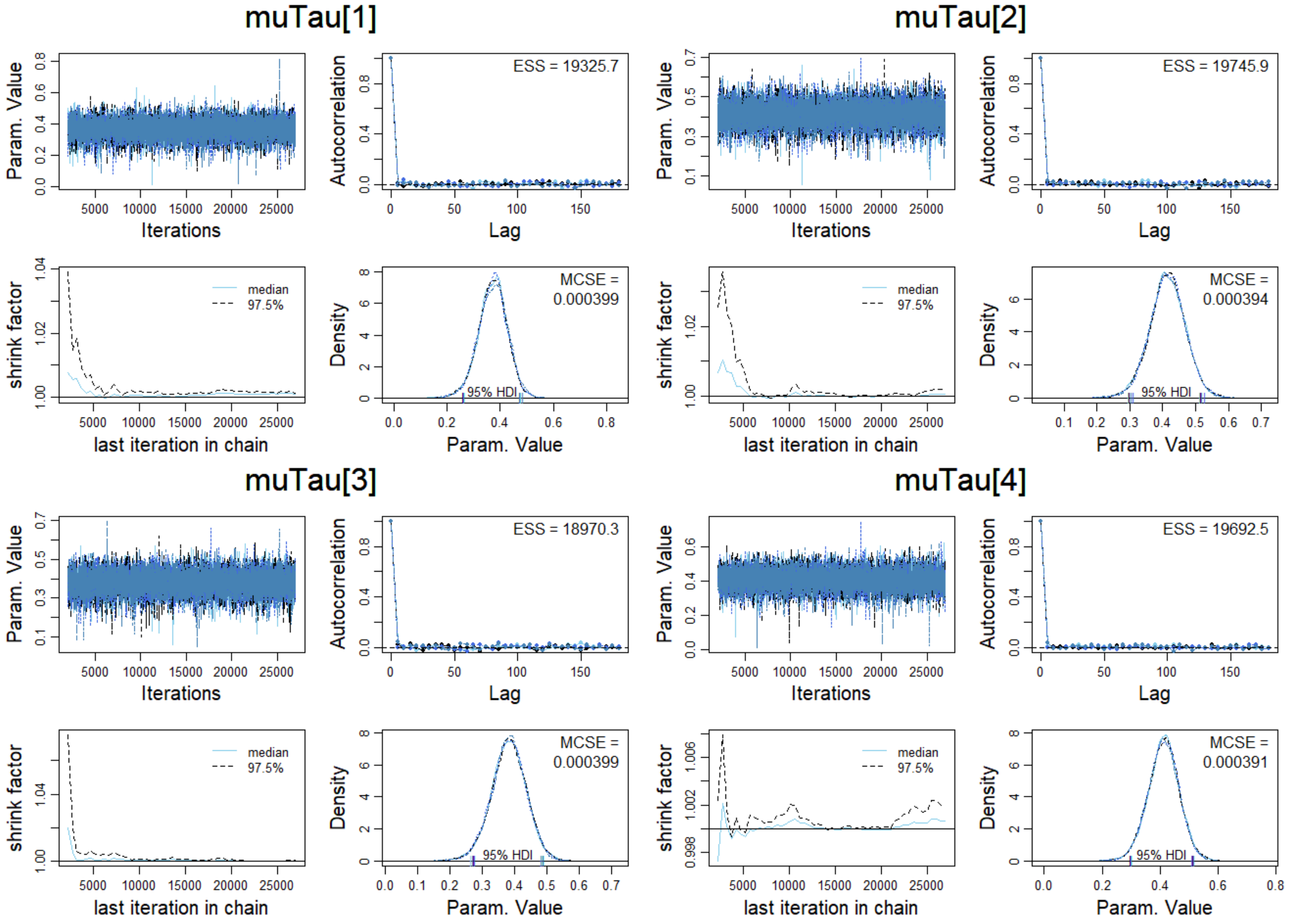

Figure 11

Experiment 2: Model Diagnostics for the Drift Diffusion Model for the Podcast Listening Condition at Pre-Measurement (Avoidance Task)

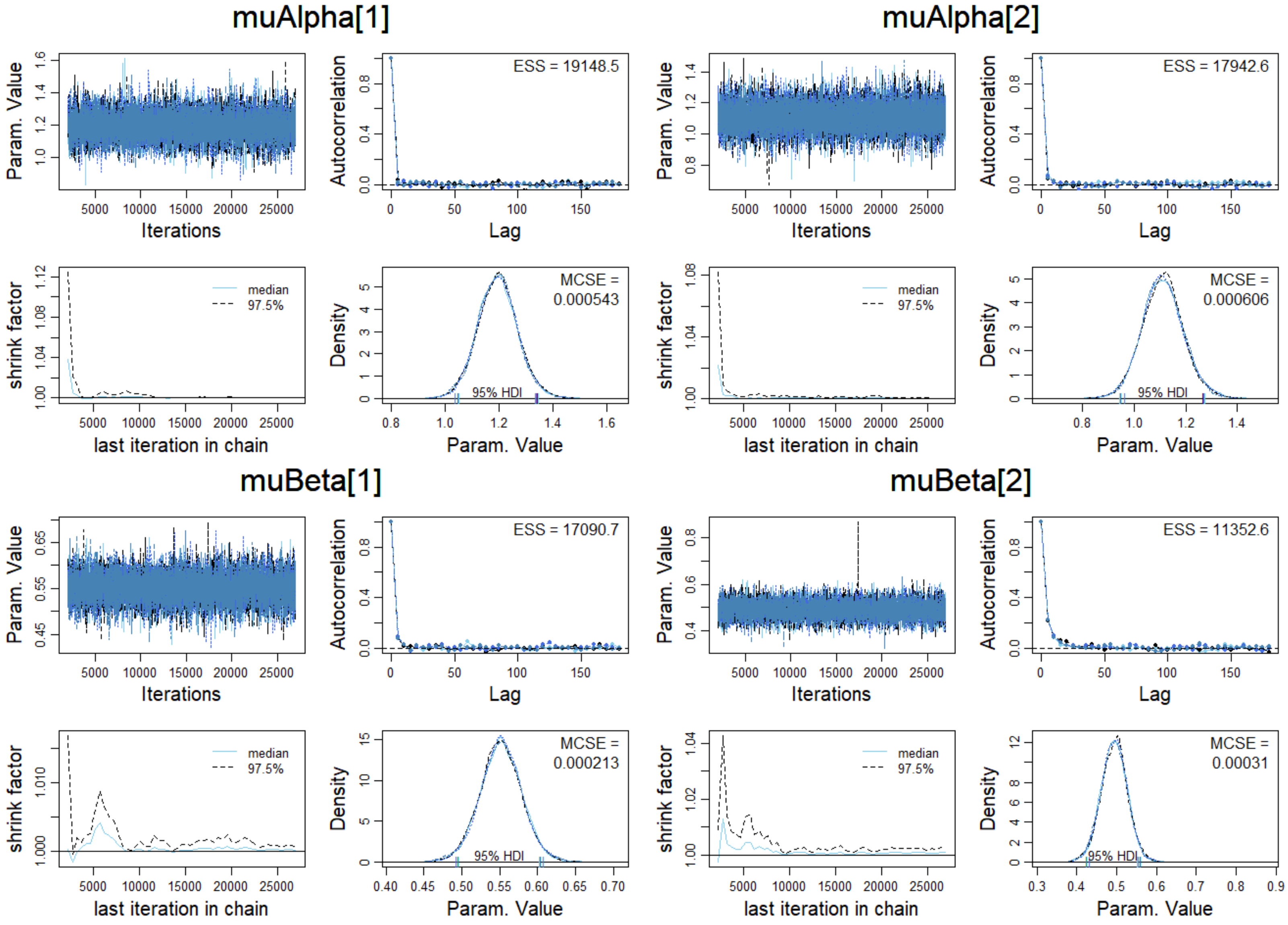

Figure 11
(continued)

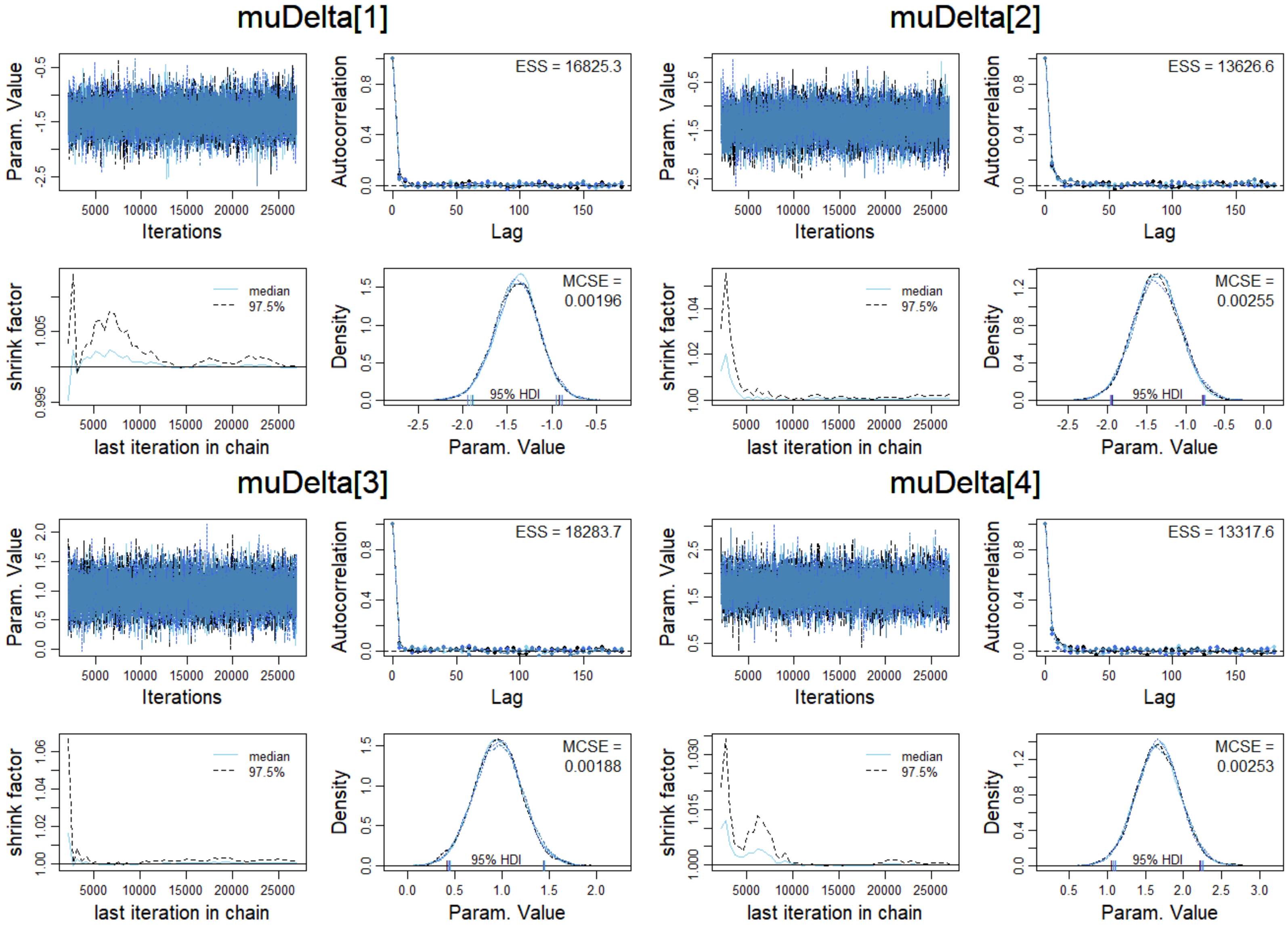

Figure 11
(continued)

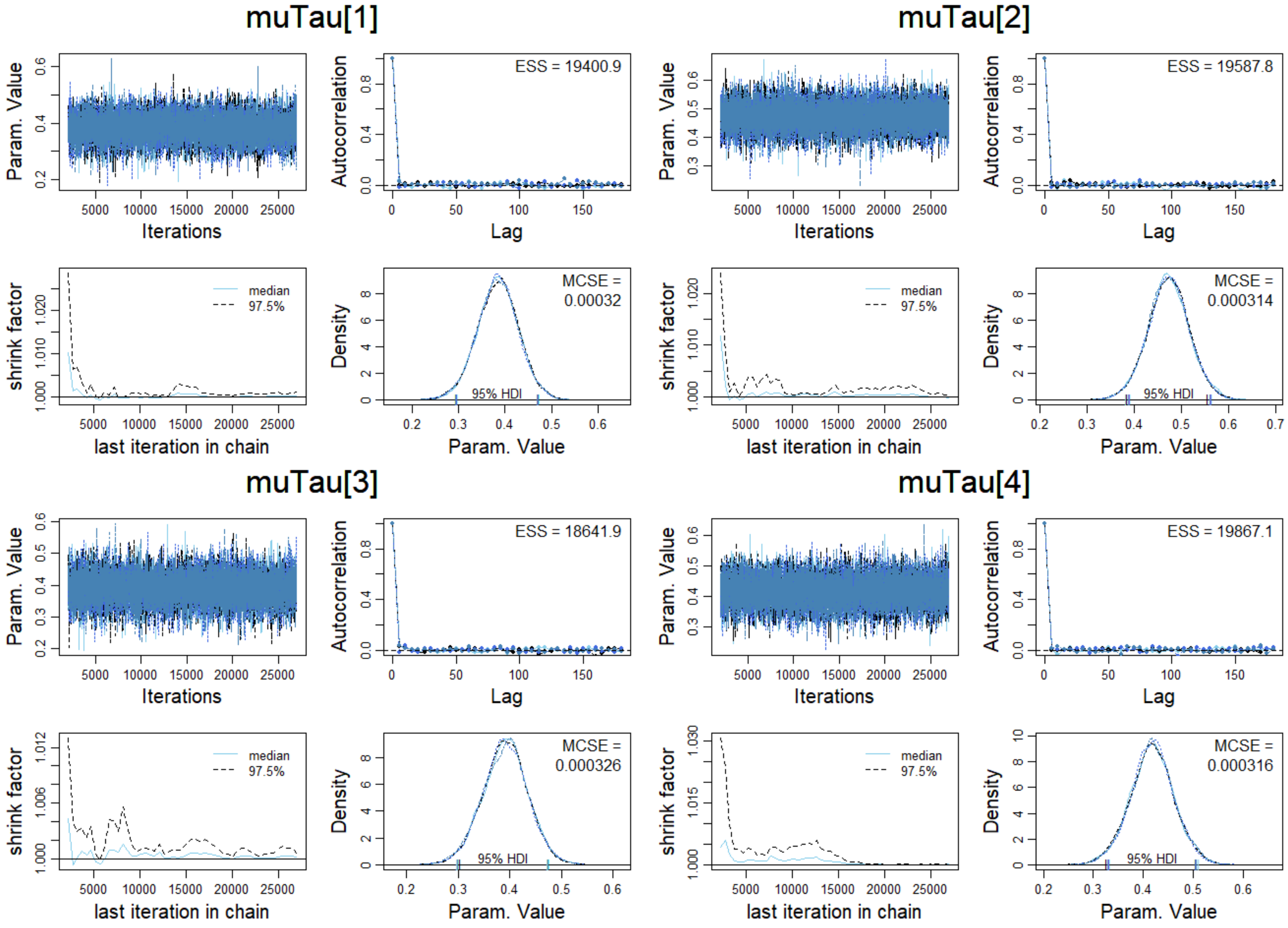

Figure 12

Experiment 2: Model Diagnostics for the Drift Diffusion Model for the Podcast Listening Condition at Post-Measurement (Avoidance Task)

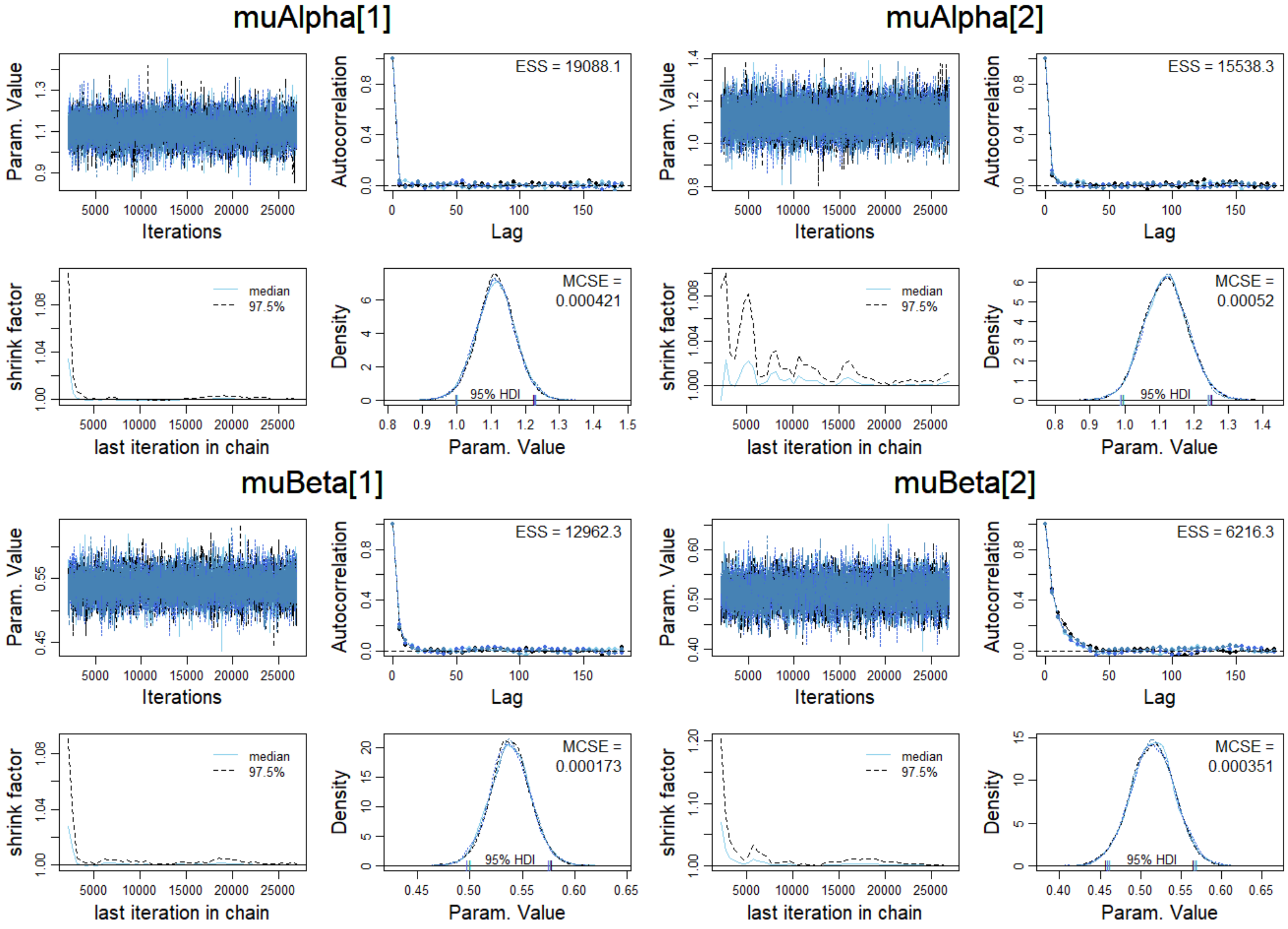

Figure 12
(continued)

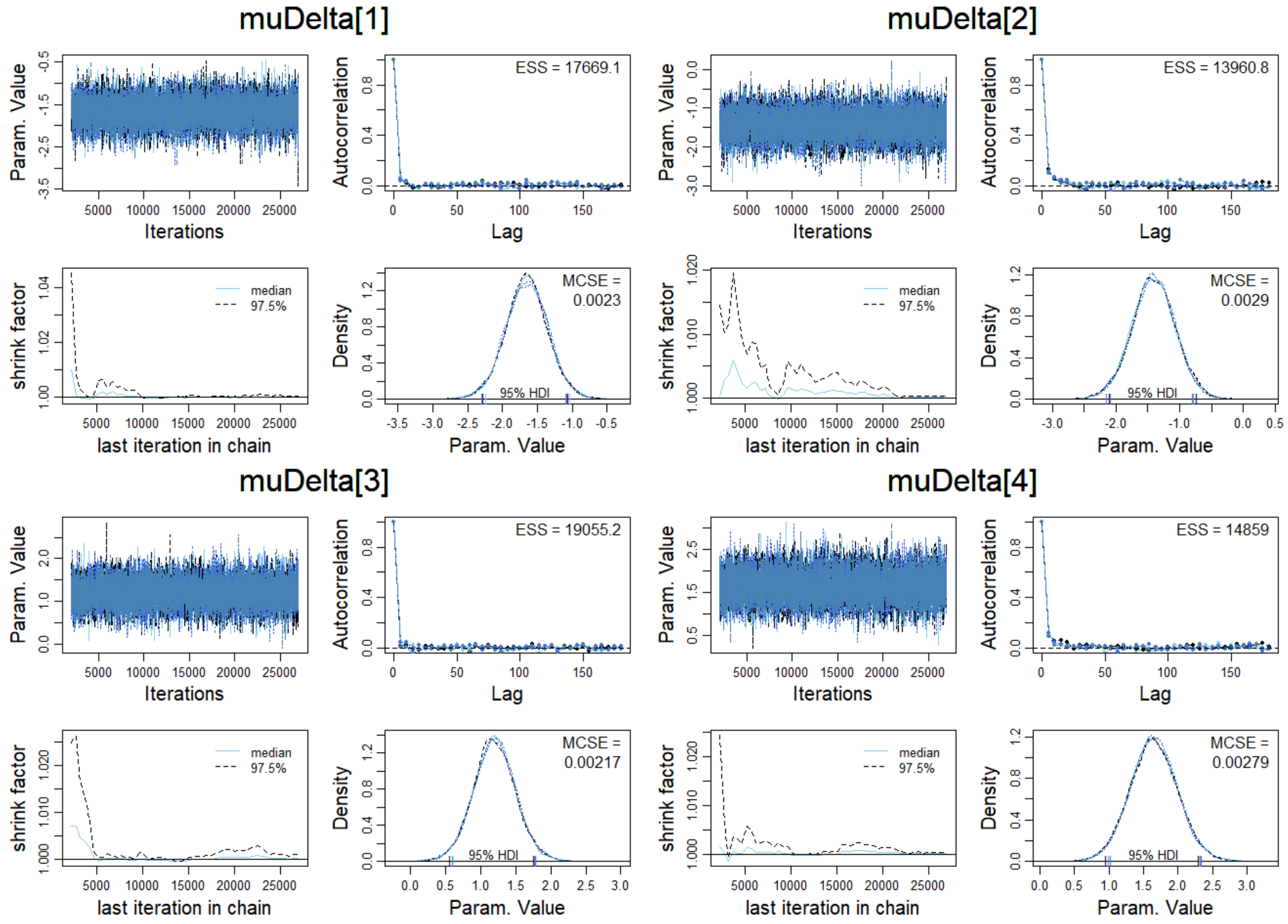

Figure 12
(continued)

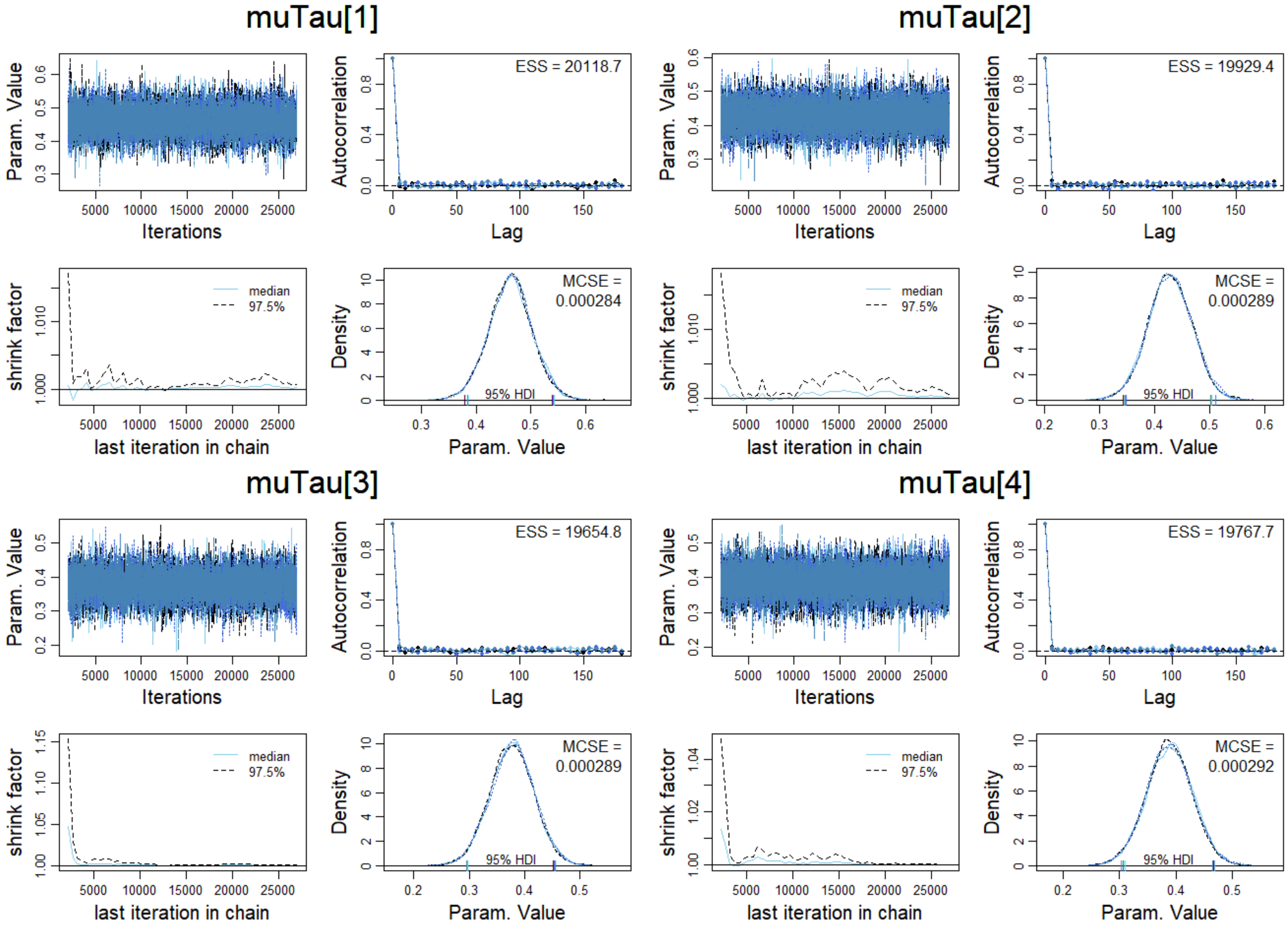

References

1. Gelman A, Rubin DB. Inference from iterative simulation using multiple sequences. Statistical science. 1992;7(4):457-72.
2. Kruschke JK. Rejecting or accepting parameter values in Bayesian estimation. Advances in methods and practices in psychological science. 2018;1(2):270-80.
3. Kruschke J. Doing Bayesian data analysis: A tutorial with R, JAGS, and Stan. 2014.
4. Gelman A, Carlin JB, Stern HS, Rubin DB. Bayesian data analysis: Chapman and Hall/CRC; 1995.
